# Universal crystallization kinetics of solution-processed semiconductors

**DOI:** 10.1038/s41467-026-78354-7

**Published:** 2026-10-14

**Authors:** Zhitian Ling, Mateusz Brzezinski, Shuanglong Wang, Konstantinos Bidinakis, Lei Gao, Chongyao Li, Xin Zhou, Hai I. Wang, Stefan A. L. Weber, Paul W. M. Blom, Wojciech Pisula, Tomasz Marszalek

**Affiliations:** 1 Max Planck Institute for Polymer Research, Ackermannweg 10, Mainz, Germany; 2 Institute of Physical Chemistry Polish Academy of Sciences, Kasprzaka 44/52, Warsaw, Poland; 3 Nanophotonics, Debye Institute for Nanomaterials Science, Utrecht University, Princetonplein 1, Utrecht, The Netherlands; 4 Department of Molecular Physics, Faculty of Chemistry, Lodz University of Technology, Zeromskiego 116, Lodz, Poland; 5 Present Address: Institute for Photovoltaics, University of Stuttgart, Pfaffenwaldring 47, Stuttgart, Germany

**Keywords:** Electronic devices, Molecular electronics

## Abstract

Heterogeneous crystallization kinetics critically govern the morphology and performance of solution-processed semiconductors, yet morphology control is often achieved through trial-and-error optimization. Quantitative understanding of crystallization is essential for precise morphology control but is limited by the simultaneous occurrence of heterogeneous nucleation and crystal growth. Here, a time-defined induced nucleation strategy has been developed to initiate crystallization at specific stages of solution processing. Systematic variation of nucleation timing reveals three distinct morphological regimes, whose emergence is explained by the concentration-dependent critical cluster radius in bulk, interfacial, and cusp environments. On this basis, a quantitative, dimensionless model is developed for heterogeneous crystallization kinetics across diverse processing conditions. The model demonstrates universality across perovskite and organic semiconductors. In situ studies reveal identical growth kinetics for induced and spontaneous nuclei, validating its generality. The resulting morphology control enables tuning of crystallinity and charge transport in solution-processed transistors.

## Abstract (web summary)

The work establishes a universal, experimentally validated model of heterogeneous nucleation and diffusion-controlled crystallization, predicting domain growth across diverse solution-processed semiconductors for efficient charge transport.

## Introduction

Solution-processed semiconductors have been widely exploited for scalable, low-cost, and flexible electronic devices, including solar cells, light-emitting diodes, and field-effect transistors (FETs)^1–3^. The morphology of the semiconductor film, particularly the size, orientation, and distribution of crystalline domains, plays a decisive role in determining charge transport and overall device performance^4,5^. While empirical strategies have enabled partial control over film morphology, the underlying crystallization kinetics remain not fully understood. A predictive and quantitative description of the collective impact of nucleation and growth on the microstructure is therefore critical for both advancing material functionality and deepening fundamental insight into thin-film formation.

Among the various physical processes that contribute to thin-film growth, heterogeneous nucleation plays an outsized role that governs the spatial and temporal onset of the crystallization, directly influencing size, density, and orientation of domains^6,7^. Unlike homogeneous nucleation, which occurs uniformly, heterogeneous nucleation is triggered by local perturbations or interfaces, turning it highly sensitive to processing conditions and consequently challenging to predict^8–10^. While many studies have attempted to control crystallization for improving the film quality and device performance through solvent, additive or processing engineering, the individual contributions of nucleation and growth are not taken into account^11–13^. Common models offer valuable insights into the overall crystallization, but fall short in resolving crystallization of individual domains to describe domain-specific growth kinetics under heterogeneous conditions. For instance, the Lamer model explains the homogeneous nucleation, but lacks the spatial and temporal resolution needed to accurately describe heterogeneous nucleation events^14–16^. The Avrami model captures the overall crystallization kinetics, but averages out domain-to-domain variability^17–19^. Models related to the late stage of the crystallization process like Ostwald ripening focus on grain coarsening and are not applicable to early-stage thin-film formation^20–22^. The main challenge in describing the crystallization of individual domains is the occurrence of parallel existing subprocesses: nucleation and growth of different domains occur in overlapping and unpredictable timeframes^23,24^. Consequently, systematically decoupling the nucleation time from the growth stage via experimental strategies is crucial for establishing a comprehensive model capable of quantitatively describing the heterogeneous crystallization, which is essential for both mechanistic insight and control over the film morphology^25–28^.

To address these challenges, we have established a time-defined induced nucleation (TDIN) strategy that enables deterministic control over the heterogeneous nucleation and allows a separate analysis of the nucleation and growth mechanisms. In this approach, artificial nucleation sites are mechanically induced into the film after defined spin-coating times, followed by thermal annealing to continue the subsequent crystal growth. The systematic variation of the coating time exposes three well-defined morphological regimes: large domains (LDs), unidirectional domains (UDs), and small domains (SDs), each emerging within a distinct spin-coating time. The transitions between these regimes occur as short as a few seconds, which is mechanistically understood based on the classical nucleation theory (CNT). To quantitatively describe the growth, we construct a piecewise model based on the mass transfer theory, capturing concentration changes during the coating and annealing stages. The model reliably describes the domain evolution for diverse materials (e.g., 2D/3D perovskites, organics, and salts), independent of specific processing conditions, and accurately predicts crystallization trajectories for both induced and spontaneous nucleation events. Based on the understanding of the crystallization mechanism, film growth controlled by induced nucleation exhibits nearly one order of magnitude higher charge carrier mobilities in field-effect transistors than the spontaneously crystallized ones. This result emphasizes the critical correlation between precise crystallization control and device functionality. While classical crystallization theories describe an ensemble-averaged behavior, the present work establishes a model to resolve and control the evolution of individual domains under heterogeneous conditions. The developed model quantitatively describing the crystallization process of solution processed semiconductors into different film morphologies provides a fundamental basis to control and optimize the film processing for specific electronic applications.

## Results and discussion

**Fig. 1:**
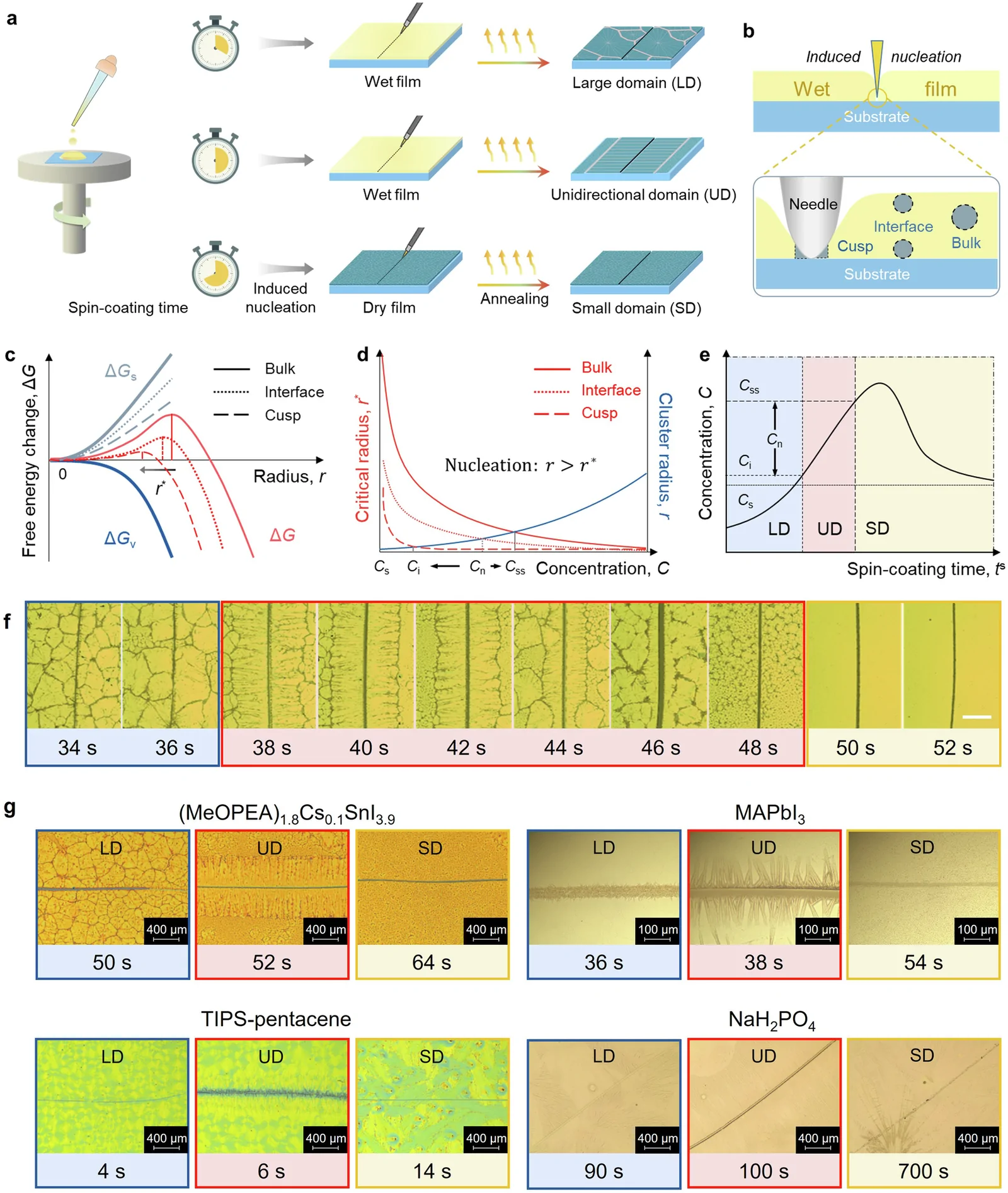
Time-defined mechanically induced nucleation enables deterministic control over crystallization and thin-film morphology. **a** Schematic illustration of the time-defined induced nucleation TDIN process during spin-coating, producing large domains LDs, unidirectional domains UDs, and small domains SDs. Nucleation is mechanically induced at different coating times to define nucleation onset, followed by thermal annealing to continue crystal growth. **b** Three types of heterogeneous nucleation sites in a wet film: bulk (within the liquid), interface (liquid/air or liquid/substrate boundary), and cusp (triple-phase solid-liquid-gas region at the contact line). **c** Change in Gibbs free energy (Δ*G*) for nucleation at different sites, schematically illustrating how nucleation barriers vary between bulk, interface, and cusp locations. Changes in surface energy (Δ*G*_s_) and volume energy (Δ*G*_v_) are also indicated. The interface curve represents heterogeneous interfacial nucleation in a general sense; the nucleation barriers at the liquid/air and liquid/substrate interfaces may differ depending on the material system and interfacial properties. **d** Red curves represent theoretical dependence of critical nucleus radius and cluster size on concentration for bulk (solid), interface (dotted), and cusp (dashed) regions. The blue curve describes the change in cluster radius with the concentration. *C*_s_ is the solubility; *C*_i_ denotes the critical concentration for mechanically induced nucleation; *C*_n_ represents the concentration for spontaneous nucleation at the interface, which is located between *C*_i_ and *C*_ss_ due to local variations in interfacial states; *C*_ss_ is the critical concentration for spontaneous bulk nucleation. **e** Schematic illustration of the relation between concentration and spin-coating time. Blue, red and yellow boxes correspond to LD, UD and SD morphologies, respectively. **f** Optical microscopy images of (PEA)_2_SnI_4_ films nucleated at coating times from 34–52 s, demonstrating the transition between LD (blue box), UD (red box), and SD (yellow box) morphologies. The scale bar is 300 µm. **g** Demonstration of the universality of the TDIN strategy across various material systems, yielding distinct morphologies (left: LD, middle: UD, right: SD) of 2D/3D perovskite (MeOPEA)_1.8_Cs_0.1_SnI_3.9_, 3D perovskite MAPbI_3_, organic molecule TIPS-pentacene and inorganic salt NaH_2_PO_4_. The scale bars are 400 µm for (MeOPEA)_1.8_Cs_0.1_SnI_3.9_, TIPS-pentacene and NaH_2_PO_4_, and 100 µm for MAPbI_3_.

In most solution-processed materials, nucleation remains a stochastic event, governed by complex and poorly defined interfacial and concentration conditions. To address this challenge, we have developed a TDIN strategy, which is based on induced heterogeneous nucleation at defined timepoints during solution processing, effectively decoupling nucleation from subsequent crystal growth (Fig. 1a). In this approach, a mechanical scratch is applied to the spin-coated film after a specific spin-coating time to artificially induce nuclei, followed by thermal annealing to initiate crystal growth. The exact moment that the nucleation is induced, termed the induced nucleation time (*t*^s^_0_), determines the crystallization pathway and final film morphology. Early-stage nucleation yields large domains (LD), intermediate nucleation generates unidirectional domains (UD), and late-stage nucleation results in small domains (SD). These regimes reflect distinct crystallization kinetics during spin-coating.

To understand the mechanistic basis of the TDIN strategy, we first discuss the formation of clusters that act as potential nucleation sites in a wet film during this event. The nucleation sites can be located in the bulk (homogeneous nucleation), the liquid/air or liquid/substrate interface (heterogeneous nucleation), where nucleation may preferentially occur depending on the material system, and the cusp-like regions (induced nucleation), illustrated in Fig. 1b. In classical nucleation theory (CNT), the change in Gibbs free energy (Δ*G*) for the cluster formation consists of two competing terms (Equation S51) graphically presented in Fig. 1c: a positive surface term (Δ*G*_S_) scaling with the square of the radius (r), and a negative volume term (Δ*G*_V_) scaling with the cube of r. A spherical approximation of the cluster is used for conceptual and analytical simplicity^29,30^. Although the actual crystal growth may deviate from an ideal spherical geometry, the key conclusions of the model rely on the relative evolution of the nucleation barrier and critical radius rather than the exact nucleus geometry. At very small cluster sizes, the Δ*G*_S_ dominates, resulting in an increase in the overall Δ*G*. As the cluster grows, the Δ*G*_V_ governs, lowering the total Δ*G*. The transition point, the critical radius (r^*^), marks the smallest cluster size at which further growth becomes energetically favorable, such that clusters can grow further to a site enabling nucleation. Compared to the bulk (solid line), clusters at the interface (dotted line) bear a reduced Δ*G*_S_, lowering Δ*G* and finally reducing the barrier to form larger clusters with subsequent nucleation. Clusters at the cusp (dashed line) show the lowest barrier due to the largest interfacial contact, turning nucleation most favorable in this region.

To understand the nucleation kinetics, it is important to realize that both the cluster size (*r*) and critical radius r^*^ are related to the concentration (*C*) of the solute in the solution in a reciprocal manner, as shown in Fig. 1d. The critical radius decreases with concentration, as derived from Supplementary Fig. 1a and explained by Equation S52. The analysis in Fig. 1d provides three curves: bulk (red solid), interface (red dotted), and cusp (red dashed), each representing a different surface energy (Δ*G*_S_). In contrast, *r* in solution (blue curve) increases with *C* and intersects the three curves at key concentrations: critical concentration for mechanically induced nucleation (*C*_i_), concentration for the spontaneous interfacial nucleation (*C*_n_), and critical concentration for spontaneous bulk nucleation (*C*_ss_). According to CNT, nucleation begins only when *r* exceeds r^*^. These thresholds directly map to the morphological regimes LD, UD, and SD in Fig. 1a, as discussed below.

The relation between *C* and *t*^s^_0_ in Fig. 1e follows a LaMer-like trend (detailed explanation is given by the description of Supplementary Fig. 1b). The concentration first increases during solvent evaporation, followed by a decrease as nucleation and growth consume solute, eventually returning to the solubility (*C*_s_) limit^31,32^. Mapping *C*_i_ and *C*_ss_ from Fig. 1d into this curve divides the TDIN process into three time regimes, each with a distinct film morphology upon induced nucleation.

### (1) Blue regime (*C* < *C*_i_, short *t*^s^_0_)

At short times, not much solvent has evaporated yet, and the concentration of solute in the wet film is still low. In this regime, *r* (blue curve in Fig. 1d) is smaller than r^*^ at the cusp (red dashed curve), and therefore nucleation cannot be mechanically induced. The formed small clusters of solutes at this low concentration will just disassemble back into the solution. During subsequent annealing, *C* gradually increases, and spontaneous interfacial nucleation is initiated only when *r* exceeds r^*^ at the interface (red dotted curve at *C*_n_). By this time, the scratch tip has left the wet film, and the continuous cusp line is no longer active for crystal growth. Consequently, the LD morphology is formed via delayed spontaneous nucleation.

### (2) Red regime (*C*_i_ ≤ *C* <*C*_ss_, intermediate *t*^s^_0_)

In this regime the concentration has already increased due to evaporation and *r* exceeds r^*^ at the cusp, but remains below r^*^ in the bulk (red solid curve). Scratching creates a continuous nucleation front promoting UD growth until *C* reaches *C*_ss_ at which bulk nucleation commences. LDs emerge later, once *C* reaches *C*_n_, since Δ*G*_S_ at the cusp is lower than at the air/liquid or liquid/substrate interface inducing an earlier UD nucleation as visualized in Supplementary Movie 1.

### (3) Yellow regime (*C* ≥ *C*_ss_, long *t*^s^_0_)

In this high concentration regime, *r* exceeds r^*^ already in the bulk (red solid curve) resulting in the formation of numerous spontaneous bulk nuclei before nucleation is mechanically induced, so that the entire film crystallizes during spin-coating into a SD morphology identical to films without scratching.

We can conclude that by controlling the supersaturation via adjusting *t*^s^_0_ it is possible to effectively control the three film morphologies by TDIN, as summarized in Fig. 1a. Importantly, nucleation and growth are not intrinsically tied to specific processing stages, i.e., spin-coating or annealing, but are governed by the evolution of solute concentration. Depending on the supersaturation trajectory, nucleation and subsequent growth may occur during spin-coating, annealing, or both. The three regimes described above represent distinct concentration-dependent crystallization pathways rather than stage-specific processes. The different growth modes are explained by the concentration dependent r^*^ trends for the nucleation in bulk, at interface, and at cusp, offering a mechanistic basis for the prediction of the film morphology. Importantly, the TDIN model does not exclude spontaneous nucleation, but instead exploits the temporal and spatial separation between induced and spontaneous nucleation pathways to control the crystallization behavior.

To verify the mechanistic framework established above, we performed TDIN experiments on PEA-based 2D perovskite films ((PEA)_2_SnI_4_) as model system, systematically varying the spin-coating time for the induced nucleation before annealing (Fig. 1f). The *t*^s^_0_ was adjusted from 34 s to 52 s for a 0.1 M precursor solution spin-coated at 2000 rpm. Mechanically inducing nucleation below 36 s does not lead to an interfacial nucleation event resulting in LD morphologies. At intermediate *t*^s^_0_ between 38 s and 48 s, the induced nucleation generates a continuous growth front yielding the UD morphology. When the induced nucleation was delayed beyond 50 s, the SD morphology is identical to the spontaneously nucleated film, indicating that bulk nucleation and film crystallization are fully presented during spin-coating. These experimentally defined time windows of TDIN correspond to the three morphological regimes introduced earlier.

Beyond (PEA)_2_SnI_4_, the TDIN strategy was further applied to other functional materials, including the 2D/3D perovskite (MeOPEA)_1.8_Cs_0.1_SnI_3.9_, 3D perovskite MAPbI_3_, organic semiconductor TIPS-pentacene, and inorganic salt NaH_2_PO_4_ (Fig. 1g). Scanning electron microscopy images further confirm that the distinct LD, UD, and SD morphologies are preserved at the microscale, in agreement with the macroscopic optical images (Supplementary Fig 2–6). We note that the absolute domain sizes associated with the LD, UD, and SD regimes can vary between materials while still preserving the characteristic morphology evolution. In all cases, the same LD-UD-SD morphological sequence is observed by varying *t*^s^_0_, demonstrating the universality of this approach across distinct crystalline materials. Detailed results of the morphology evaluation of each material upon induced nucleation are provided in the SI (Supplementary Fig. 7). In these datasets, LD domains are randomly distributed in both position and size, reflecting the stochastic nature of their nucleation events. This irregularity makes it challenging to isolate this domain growth for quantitative analysis. In contrast, UD domains exhibit a clear, systematic reduction in size with increasing *t*^s^_0_, which is a direct consequence of delaying the nucleation onset. This well-defined temporal evolution not only enables robust modeling of the crystal growth, but also serves as a controllable analog for the otherwise unpredictable timing of the spontaneous LD nucleation.

**Fig. 2:**
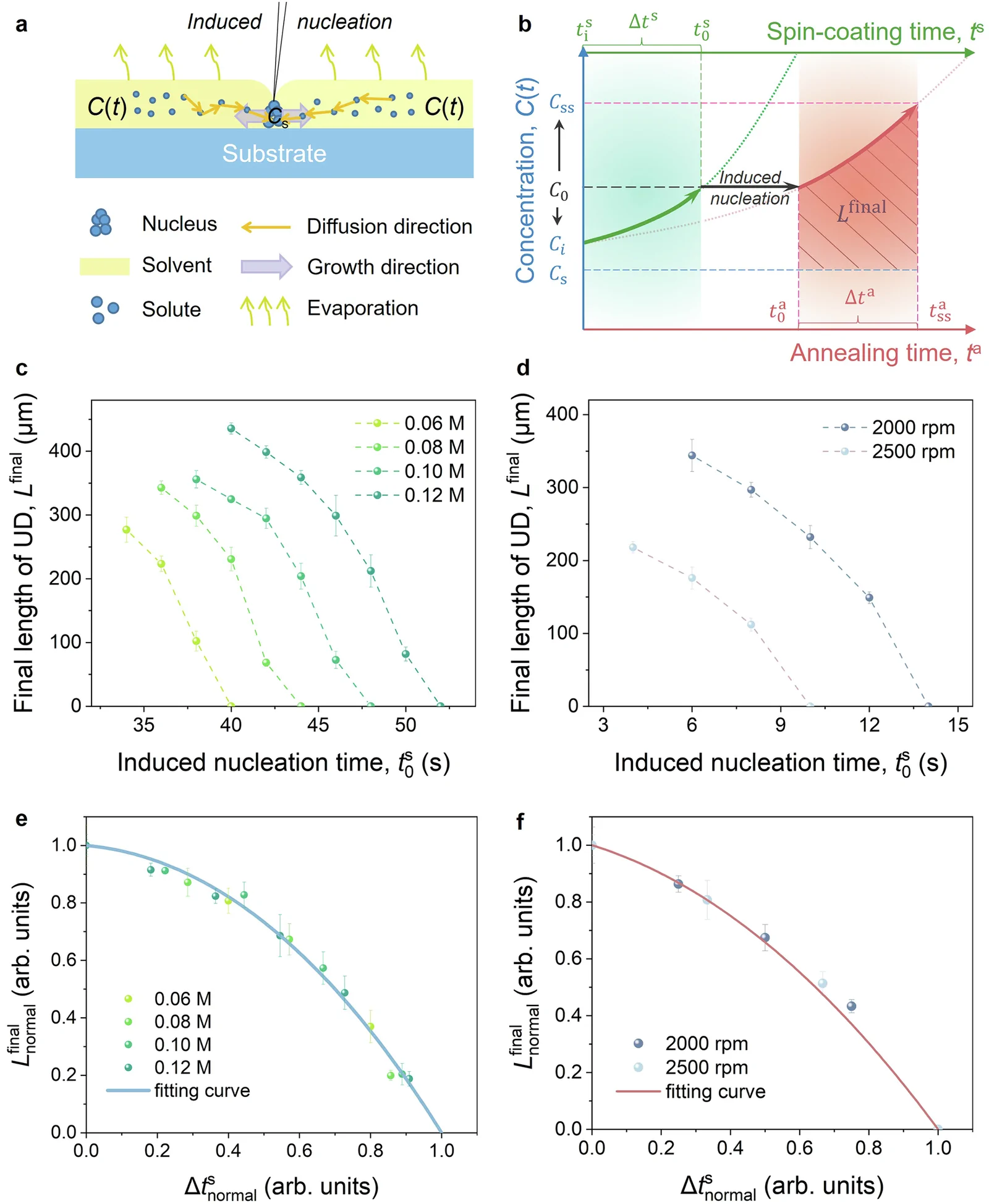
Development of the crystallization kinetics model. **a** Schematic illustration of the crystallization process of unidirectional domains (UDs). **b** Modeling the relation between the concentration of the wet film to spin-coating time (*t*^s^) and annealing time (*t*^a^). Relation between average final length of UDs (*L*^final^) and induced nucleation time (*t*^s^_0_) for (**c**) different initial concentrations (*C*^init^) of (PEA)_2_SnI_4_ and (**d**) different spin-coating speeds (*ω*) of TIPS-pentacene. Fitting curves based on the model and the relation between normalized final length (*L*^Final^_normal_) and normalized spin-coating interval (Δ*t*^s^_normal_) for various (**e**) *C*^init^ of (PEA)_2_SnI_4_ and (**f**) *ω* of TIPS-pentacene. Error bars represent the standard deviation of the measurements.

To quantitatively explain the systematic decrease in UD size with *t*^s^_0_, the relevant growth kinetics following nucleation must be considered. When nuclei are mechanically induced, crystallization starts due to mass transport resulting from the concentration difference between the wet film and the nuclei surface, as illustrated in Fig. 2a. The concentration at the surface of the nuclei is fixed at *C*_s_, whereas the concentration in the wet film increases with the spin-coating and annealing times, defined as C(*t*). Once the solute reaches the nuclei surface, crystal growth proceeds and the UDs grow perpendicular to the induced nucleation line. Since the interfacial reaction at the nuclei surface is assumed to be significantly faster than solute diffusion, in this case, the growth is diffusion-controlled^33–35^.

As demonstrated in Fig. 2b, the nucleation is induced at the concentration (*C*_0_), which is located between *C*_i_ and *C*_ss_, to emulate distinct *C*_n_ scenarios shown in Fig. 1e and to formulate a diffusion-controlled growth model for TDIN. In the first spin-coating stage, the concentration rises from *C*_i_ to *C*_0_, at which nucleation in the wet film is induced, as indicated by the green solid curve in Fig. 2b. In the second stage, annealing after the induced nucleation and transfer onto the hot plate, the concentration further increases from *C*_0_ to *C*_ss_ marked by the red solid curve. The changes in concentration during sample transfer (≤1 s) are neglected relative to the spin-coating and annealing durations. To quantitatively describe the UD growth, a diffusion-based model is established under three simplifying assumptions: (1) the substrate is effectively infinite relative to the UD dimensions; (2) UD growth ceases once the wet-film concentration reaches *C*_ss_, at which bulk nucleation dominates; and (3) solvent evaporation is spatially uniform during spin-coating and annealing, with areal evaporation fluxes *Q*_spin_ and *Q*_anneal_, respectively. Under these assumptions, the concentration evolution is defined as

1 $$C=\left\{\begin{array}{c}\frac{{C}_{{{{\rm{i}}}}}}{1-\frac{{Q}_{{{{\rm{spin}}}}}{C}_{{{{\rm{i}}}}}}{\alpha }\left({t}^{{{{\rm{s}}}}}-{t}_{{{{\rm{i}}}}}^{{{{\rm{s}}}}}\right)},\,{t}_{{{{\rm{i}}}}}^{{{{\rm{s}}}}}\le {t}^{{{{\rm{s}}}}}\le {t}_{0}^{{{{\rm{s}}}}}\,\left({{{\rm{spin\; coating}}}}\right)\\ \frac{{C}_{{{{\rm{ss}}}}}}{1-\frac{{Q}_{{{{\rm{anneal}}}}}{C}_{{{{\rm{ss}}}}}}{\alpha }\left({t}^{{{{\rm{a}}}}}-{t}_{{{{\rm{ss}}}}}^{{{{\rm{a}}}}}\right)},\,{t}_{0}^{{{{\rm{a}}}}}\le {t}^{{{{\rm{a}}}}}\le {t}_{{{{\rm{ss}}}}}^{{{{\rm{a}}}}}\,\left({{{\rm{annealing}}}}\right)\end{array}\right.$$

where *α* is the concentration coefficient that is described in Equation S8. Here *t*^s^ and *t*^a^ denote the spin-coating and annealing times, respectively; *t*^s^_i_corresponds to the spin-coating time when *C* = *C*_i_, *t*^s^_0_ is the spin-coating time when *C* = *C*_0_, *t*^a^_0_ is the annealing time when *C* = *C*_0_, and *t*^a^_ss_ marks the annealing time when *C* = *C*_ss_. The spin-coating and annealing intervals in Fig. 2b are defined as Δ*t*^s^ = *t*^s^_0_ - *t*^s^_i_, in which *C*_i_ increases to *C*_0_, and Δ*t*^a^ = *t*^a^_ss_ - *t*^a^_0_ during the increase from *C*_0_ to *C*_ss_, respectively. These two intervals follow the relationship as described in Equation S12 and Supplementary Fig. 8.

Since the growth is diffusion-limited, the final UD length (*L*^final^) is proportional to the shadow area within the annealing stage in Fig. 2b and the following definition is provided:

2 $${\int }_{0}^{{L}^{{{{\rm{final}}}}}}\frac{{\rho }_{{{{\rm{solute}}}}}}{M{k}_{{{{\rm{c}}}}}}{{{\rm{d}}}}L={\int }_{{t}_{0}^{{{{\rm{a}}}}}}^{{t}_{{{{\rm{ss}}}}}^{{{{\rm{a}}}}}}\left(C(t)-{C}_{{{{\rm{s}}}}}\right){{{\rm{d}}}}{t}^{{{{\rm{a}}}}}$$

where *k*_c_ is the mass-transfer coefficient which is treated as a constant for each material^36^. In Figs. 2c, d*L*^final^ is plotted as a function of *t*^s^_0_ for (PEA)_2_SnI_4_ and TIPS-pentacene for different initial concentrations (*C*^init^) and spin-coating speeds (*ω*), respectively. The data points are extracted from the optical micrographs in Supplementary Figs 9 and S10. At a fixed *C*^init^ or *ω*, *L*^final^ decreases with increasing *t*^s^_0_ since an increased *t*^s^_0_ leads to a larger Δ*t*^s^ and decreased Δ*t*^a^, which eventually leads to a smaller *L*^final^, as described by the model and thus illustrated by the shadow area in Fig. 2b. Raising *C*^init^ or reducing *ω* shift the entire experimental curves in Fig. 2c, d to larger *L*^final^ values, since a higher *C*^init^ or a slower *ω* yields thicker dry films, which in turn increases *L*^final^, as described by Equation S34.

To minimize material-specific parameters (like *α*, *k*_c_, *C*^init^ or *ω*) and simplify the description of the model, the final length of UDs and spin-coating intervals are normalized into *L*^final^_normal_ = *L*^final^/ *L*^final^_max_ and Δ*t*^s^_normal_ = Δ*t*^s^/ Δ*t*^s^_max_, respectively. The relation between *L*^final^_normal_ and Δ*t*^s^_normal_ is described in Equation 3 by introducing two dimensionless parameters (*R* = *C*_s_/ *C*_ss_ and *S*_Si_ = *C*_i_/ *C*_s_):

3 $$ 	 \left(R-{{\mathrm{ln}}}\left(R{S}_{{{{\rm{Si}}}}}\right)-\frac{1}{{S}_{{{{\rm{Si}}}}}}\right){L}_{{{{\rm{normal}}}}}^{{{{\rm{final}}}}}={{\mathrm{ln}}}\left(1-\left(1-R{S}_{{{{\rm{Si}}}}}\right){\Delta t}_{{{{\rm{normal}}}}}^{{{{\rm{s}}}}}\right)\\ 	 -\frac{1-\left(1-R{S}_{{{{\rm{Si}}}}}\right){\Delta t}_{{{{\rm{normal}}}}}^{{{{\rm{s}}}}}}{{S}_{{{{\rm{Si}}}}}}+R-{{\mathrm{ln}}}\left(R{S}_{{{{\rm{Si}}}}}\right)$$

As shown in Figs. 2e, f, all datasets from Figs. 2c, d are collapsed onto one single curve using only the parameter pair of *R* and *S*_Si_ per material. The best-fit values and coefficient of determination are reported in Table S1. The same data transformation is performed for the 2D/3D perovskite (MeOPEA)_1.8_Cs_0.1_SnI_3.9_, as shown in Supplementary Fig. 11, further supporting the generality of the model. The consistent agreement between experiment and model across distinct processing conditions and materials indicates that UD growth follows a universal kinetic mechanism. More importantly, the ability to define the concentration-dependent nucleation window provides a practical model for controlling the crystallization pathways and improving film uniformity in solution-processed semiconductor films, particularly for large-area processing.

To further examine the generality of the proposed model under more complex processing conditions, we extended the analysis to a two-step spin-coating process, as shown in Supplementary Fig. 12. Despite the staged spin-coating protocol, the crystallization behavior exhibits a similar morphological evolution from LD to UD and finally to SD, consistent with the one-step process. Notably, the onset of the UD formation occurs at an earlier time, accompanied by a compressed processing window Δ*t*^s^ for the UD regime compared to the one-step process (Supplementary Fig. 9). This behavior is attributed to the increased solvent evaporation rate during the second high-speed spinning step, which accelerates the concentration increase in the wet film. As a result, the wet film reaches *C*_i_ earlier, and the transition from *C*_i_ to *C*_ss_ proceeds within a shorter time interval. These observations are fully consistent with the model, independent of the specific spin-coating protocol. This result further highlights the robustness of the proposed crystallization kinetics and its applicability to more realistic multi-step processing conditions.

As a summary, the proposed model consists of two key components: (i) a nucleation model based on CNT, which defines the critical concentrations (*C*_i_ and *C*_ss_) governing the onset of induced and spontaneous nucleation, and (ii) a diffusion-controlled growth model that quantitatively describes domain evolution after nucleation. The applicability of this framework relies on systems where the concentration evolution is governed by solvent evaporation and crystal growth is limited by mass transport. Under these conditions, the crystallization pathway can be described by the parameter pair of *R* and *S*_Si_, which links nucleation and growth across different processing conditions.

**Fig. 3:**
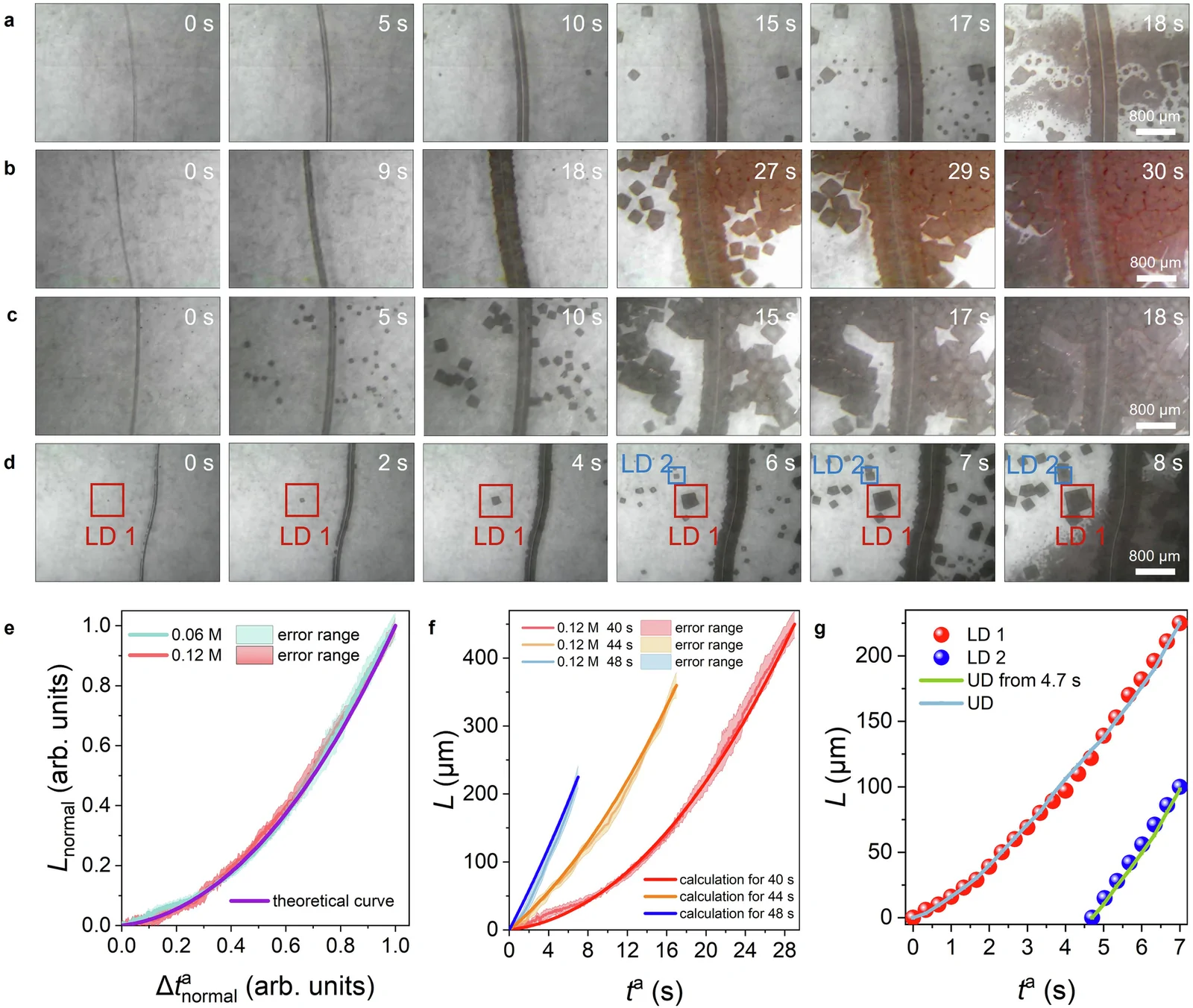
Experimental validation of the crystallization kinetics model. Movie snapshots of the (PEA)_2_SnI_4_ crystallization at different initial concentrations (*C*^init^) and induced nucleation time (*t*^s^_0_): (**a**) *C*^init^ = 0.06 M and *t*^s^_0_ = 34 s, (**b**) *C*^init^ = 0.12 M and *t*^s^_0_ = 40 s, (**c**) *C*^init^ = 0.12 M and *t*^s^_0_ = 44 s and (**d**) *C*^init^ = 0.12 M and *t*^s^_0_ = 48 s. The scale bar is 800 µm. **e** Relation between normalized growth length (*L*_normal_) and normalized annealing time (Δ*t*^a^_normal_) for the crystallization shown in Fig. 3a, b and the theoretical curve derived from the model. **f** the time-dependent length (*L*) of the unidirectional domains (UDs) as a function of annealing time (*t*^a^) for the crystallization shown in Fig. 3b–d with curves calculated from the model. **g** Change in length of the crystal diagonal of large domain (LD) during annealing time for LD1, marked by red box, and LD2, marked by blue box, shown in Fig. 3d together with the UD growth. LD1 and LD2 nucleate at 0 s and 4.7 s, respectively. The error range represents the standard deviation of distance measurements across sub-regions of the crystal.

Besides the description of the *L*^final^, the model is further applied to understand the growth kinetics of the domains. Thereby, the time-dependent UD length (*L*) during the annealing is defined to be distinct from *L*^final^ introduced earlier. As shown in Supplementary Fig. 13, *L*^final^ is determined from optical microscopy images after crystallization is completed, while *L* is extracted from time-resolved movie frames during crystallization. In order to describe the change of *L* during annealing, the growth model from Fig. 2b is modified into Supplementary Fig. 14 based on Equation S32. As described by the model, the relation between *L* and *t*^a^ can be predicted by applying the dimensionless parameters *R* and *S*_Si_ that are defined previously. The prediction of *L* and *t*^a^ is first evaluated under ideal onset conditions of the UD growth of (PEA)_2_SnI_4_. As shown in Fig. 3a, b the crystallization was monitored for *C*^init^ of 0.06 M and 0.12 M and nucleation was induced at *t*^s^_0_ of 34 s and 40 s, respectively, corresponding to the ideal onset points of the UD growth window indicated by the frames in Supplementary Fig. 15a. Time-lapse optical micrographs of the crystallization process of the two films were extracted from recordings of Supplementary Movie 1 and 2, and plotted in Supplementary Fig. 16a. The corresponding LD, UD, and SD regions in Fig. 3a–d are more clearly labeled in Supplementary Fig. 17. The UD growth completes at 17 s and 29 s for 0.06 M and 0.12 M, respectively, ~1 s before the entire film is crystallized as shown in the final images of Figs. 3a and 3b. Normalized *L* versus *t*^a^ curves for both concentrations in Fig. 3e are in agreement with the predicted theoretical curve derived from the model using the parameter pair of *R* and *S*_Si_ as 0.85 and 1.01 as obtained from Fig. 2e, respectively. This accordance between experimental and theoretical results confirms that the model accurately captures the UD growth kinetics regardless of the initial processing conditions if the nucleation is induced at the onset of the growth window.

To further evaluate the applicability and generality of the model, the crystallization kinetics were examined under conditions when nucleation is induced within the UD growth window and not at its onset that is under *C*_0_ ≠ *C*_i_. This study addresses a more realistic scenario when nucleation is initiated at arbitrary coating times during processing. The UD growth was initiated at *t*^s^_0_ of 44 s and 48 s in two wet films of the same *C*^init^ of 0.12 M shown in Fig. 3c, d, corresponding to the UD window indicated by the frames in Supplementary Fig. 15b. The growth length of the UDs during annealing was extracted from Supplementary Movies 2 to 4 and plotted in Fig. 3f. For the fixed *C*^init^, the growth can be described by the Equations S42 to S50 with the main inputs of *t*^s^_0_, *R* and *S*_Si_. Despite the different *C*_0_ values at the three nucleation times, the experimentally monitored growth closely follows the theoretical curves provided by the model. Additionally, to the previously discussed projection of the growth initiated at the onset of the growth window, this agreement confirms that the model quantitatively also describes the UD crystallization even when the nucleation occurs at arbitrary times and processing conditions within the operation window. Importantly, this analytical capability enables rational design of the processing conditions of an optimized film crystallization for various applications. By choosing a desired induced nucleation time, it is possible to anticipate the complete domain growth and thereby tailor the final film morphology to meet process and device constraints.

To assess whether the model also applies to spontaneously nucleated domains, the growth of LDs was investigated and compared to UD growth under identical conditions. As also shown in Fig. 3d, two LDs, marked as LD1 and LD2 and highlighted by red and blue boxes, successively nucleate at different times. LD1 appears simultaneously with the UD, while LD2 emerges 4.7 s later. The growth trajectories of both LDs were extracted from Supplementary Movie 4 and plotted in Supplementary Fig 16b. To enable direct comparison with the UD growth, the size of both LDs was defined as half of the diagonal length of the square domain. Using this metric, the growth of LD1 coincides with the UD during the same growth time, while LD2, once nucleated after 4.7 s, and also evolves along the same growth profile as the UD as shown in Fig. 3g. This confirms that each nucleus, once formed, grows according to the same universal, diffusion-controlled mechanism, regardless of whether nucleation is induced (for UD) or spontaneous (for LD).

Since these nucleation events occur at different annealing times, the local concentration at the moment of nucleation varies for each domain. As a result, although governed by the same growth law, each domain exhibits a distinct crystallization kinetics determined by the nucleation time. Notably, earlier nucleated LDs such as LD1 initially grow at reduced growth rate due to lower supersaturation, while later ones, like LD2, experience faster initial growth, as demonstrated in Supplementary Fig 16b, under steeper concentration gradient between wet film (*C*) and surface of the cluster (*C*_s_). Thus, each domain crystallizes under a distinct driving force, leading to domain-specific growth kinetics that may affect spatial uniformity and overall film quality. These insights reinforce the significance of the model since it captures both the intrinsic kinetics shared by all domains and the extrinsic impact introduced by different nucleation times. Consequently, the model offers a unified framework for understanding and controlling crystallization in solution-processed films.

**Fig. 4:**
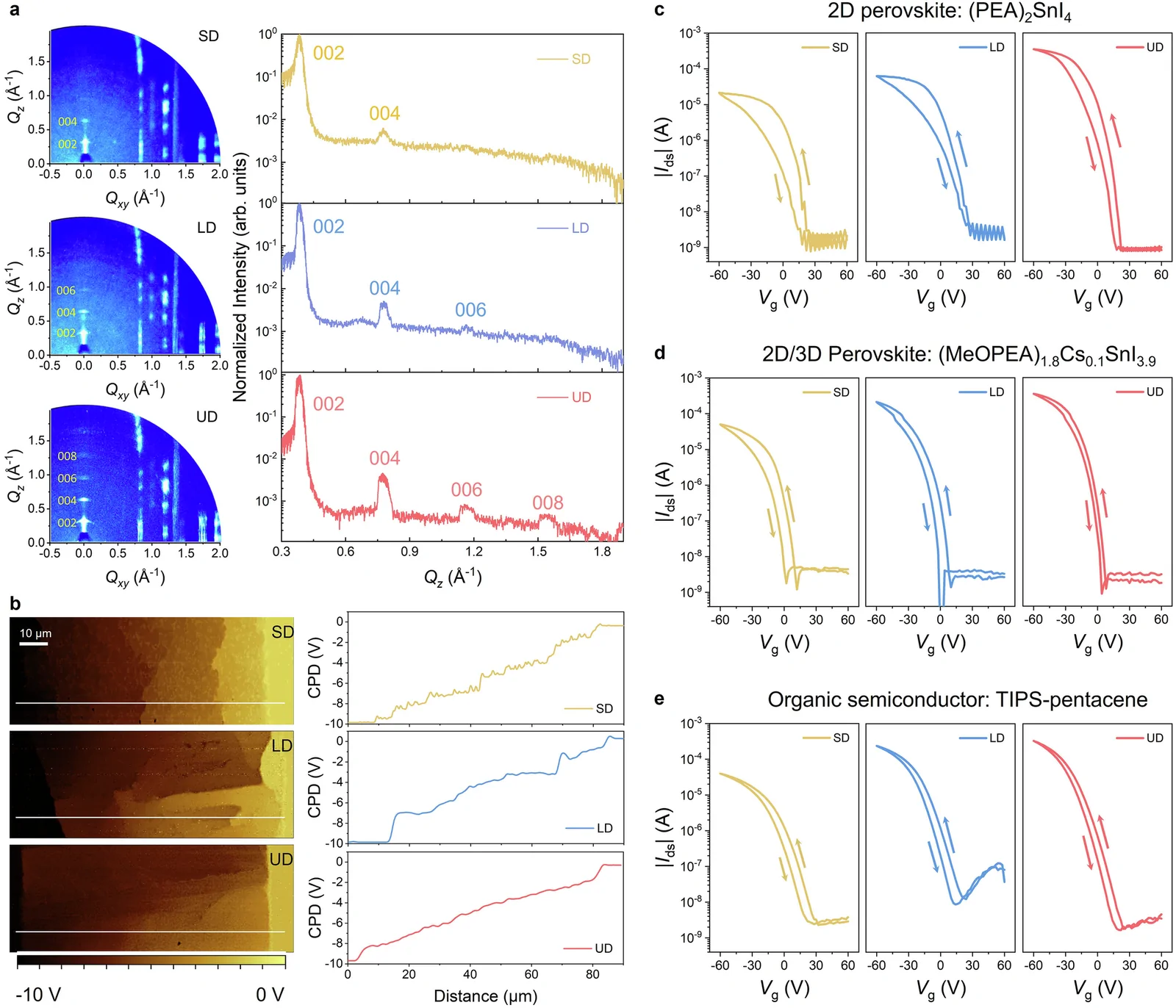
Structure and charge transport of different crystallization morphologies. **a** Grazing incidence wide-angle X-ray scattering patterns for (PEA)_2_SnI_4_ films with small domain SD, large domain LD and unidirectional domain UD, respectively, and the corresponding out-of-plane intensity profiles (selected reflections are assigned by Miller indices). *Q*_*z*_ and *Q*_*xy*_ denote the out-of-plane and in-plane components of the scattering vector, respectively. **b** Kelvin probe force microscopy (KPFM) contact potential difference (CPD) maps and corresponding profiles for field-effect transistors with SD, LD and UD morphologies recorded by KPFM at gate bias of −44 V and drain bias of −9 V with a grounded source. Transfer characteristics (|*I*_ds_ | -*V*_g_) at room temperature for the three different film morphologies for (**c**) 2D perovskite (PEA)_2_SnI_4_, **d** 2D/3D perovskite (MeOPEA)_1.8_Cs_0.1_SnI_3.9_ and (**e**) organic semiconductor TIPS-pentacene.

After systematically and quantitatively understanding the crystallization kinetics, the impact on the film quality was further studied for (PEA)_2_SnI_4_ as an illustrative model material. The three distinct morphologies were obtained from a solution with *C*^init^ of 0.06 M by TDIN setting *t*^s^_0_ at 32 s for LD, 34 s for UD and 42 s for SD. To investigate the influence of distinct crystallization pathways on the resulting structural and electronic properties, representative LD, UD, and SD regions of the films were selectively characterized. Grazing incidence wide-angle X-ray scattering (GIWAXS) was exploited to evaluate the crystal structure, and the results are shown in Fig. 4a. The highest out-of-plane intensity is attributed to the interlayer (002) reflection corresponding to a d-spacing of 16.3 Å that is in agreement with literature^37,38^. The higher number of the (00 l) reflections for the UD morphology indicates long-range crystals (l = 2, 4, 6, 8) in comparison to the LD film with large crystals (l = 2, 4, 6) and SD morphology with small crystals (l = 2, 4). The larger number of higher-order reflections indicates a well-ordered structure of the UD film among the three samples^39,40^. The out-of-plane (002) interlayer diffraction peak was further analyzed to determine the crystallinity. The coherence length (CL) increases from 373 Å for SD, to 414 Å and 421 Å for LD and UD, respectively. The larger CL for the UD film is associated with its higher crystallinity, confirming higher molecular order along the out-of-plane direction. The UD film also reveals narrower and split in-plane diffraction features compared to the SD and LD morphologies arising from multi-domain formation with similar orientations, indicating an enhanced in-plane crystallinity and domain size.

To further confirm the film quality along the in-plane direction, optical and atomic force microscopy images in Supplementary Fig. 18 are analyzed. SD films consist of 2 µm domains in size surrounded by pinholes resulting in a root mean square roughness (RMS) of 9.6 nm. The LD films exhibit a larger domain size of around 300 µm and a lower RMS of 6.7 nm. The UD films reveal a pinhole-free surface and the lowest RMS of 1.9 nm among the studied series. Potential maps and corresponding profiles of a transistor channel for the three film morphologies were recorded by Kelvin probe force microscopy (KPFM) as shown in Fig. 4b. The mapping of the local potential allows to evaluate the influence of the film morphology on the electrical properties. A configuration with biased electrodes was used to simulate operating conditions above the threshold voltage of the FET (Supplementary Fig. 19). For the SD film, the potential distribution shows intermittent voltage drops with strong fluctuations at the domain boundaries. These boundaries act as structural defects and trapping sites for carriers, thereby impeding transport and resulting in low effective charge carrier mobility^41–43^. A random orientation of long domain boundaries in the LD film creates irregular potential maps and resistive barriers across the channel, causing significant voltage drops despite relatively high local charge carrier mobility within each domain, as discussed below. In contrast, the UD film exhibits a nearly linear potential decline with only minor fluctuations, reflecting the reduced density of boundaries and indicating more efficient carrier transport, which underpins their superior device performance.

To assess these structural and electronic features on a macroscopic scale, the FET performance of the films was subsequently evaluated. The spatially differentiated morphologies enable a direct comparison between distinct crystallization pathways and their corresponding electronic properties. The device geometry with the three film morphologies is shown in Supplementary Fig. 20. The transfer characteristics were recorded at room temperature at drain-source voltages (*V*_ds_) of −60 V with gate-source voltage (*V*_g_) scans in forward and backward directions. As shown in Fig. 4c, the devices based on UDs exhibit the highest FET mobility (μ_FET_) of 2.6 cm^2 ^V^−1^ s^−1^ which is significantly improved compared to LDs with 0.6 cm^2 ^V^−1^ s^−1^ and SDs with 0.2 cm^2 ^V^−1^ s^−1^, as calculated from the transfer curves in the forward direction. For comparison, the local charge carrier mobility determined by optical pump-terahertz probe spectroscopy measurements (μ_OPTP_) is shown in Supplementary Fig. 21. The μ_OPTP_ of 8.9 and 8.4 cm^2 ^V^−1^ s^−1^ for LD and UD films, respectively, are nearly identical. In contrast, the μ_OPTP_ for SD films decreases to 6.3 cm^2 ^V^−1^ s^−1^. While the macroscopic μ_FET_ and local μ_OPTP_ charge carrier mobilities are on the same order of magnitude for the UD morphology, the device mobility for LDs and especially for SDs is decreased by more than one order of magnitude due to the reduced order and higher density of domain boundaries, as proven by the GIWAXS, AFM and KPFM results. Furthermore, transient absorption measurements in Supplementary Fig. 22 show comparable carrier relaxation dynamics among LD, UD, and SD films, suggesting that the excited-state carrier dynamics within the crystalline domains are broadly similar across the three morphologies. Additional time-resolved photoluminescence measurements in Supplementary Fig. 23 reveal similar decay dynamics for the LD and UD films, whereas the SD film exhibits a faster decay, indicating enhanced defect-assisted non-radiative recombination in the SD film. This observation is consistent with the photoconductivity dynamics shown in Supplementary Fig. 21, suggesting that the superior FET performance mainly arises from continuous transport pathways along the aligned domains with reduced density of boundaries rather than lower defect density. The FETs based on the UD morphology also show the highest channel current and on/off ratio (*I*_on/off_) of 5 × 10^5^, the smallest dual-sweep hysteresis (Δ*V*) of 10.2 V (at |*I*_ds_ | = 10^−6 ^A in both sweep directions), the lowest threshold voltage (*V*_TH_) of 6.1 V, and the lowest subthreshold swing (SS) of 5.0 V dec^−1^, indicating improved device performance compared to the two other film morphologies as summarized in Table S2. The enhanced device performance of the UD morphology is attributed to its anisotropic domain growth and continuous charge-transport pathways, which reduce domain-boundary as trapping sites across the device channel. The output characteristics for the corresponding devices with various morphologies are plotted in Supplementary Fig. 24a revealing the highest gate modulation of the current for the UD perovskite FET. Bias-stability measurements in Supplementary Fig. 25 further reveal that UD devices exhibit the slowest current degradation under continuous gate and drain bias stress, followed by LD and SD devices, indicating improved operational stability associated with the reduced domain boundary density in UD films.

To evaluate the generality of the three types of film morphologies beyond the model (PEA)₂SnI_4_ system, two chemically distinct semiconductors were additionally examined consisting of (MeOPEA)_1.8_Cs_0.1_SnI_3.9_ (Fig. 4d), and TIPS-pentacene (Fig. 4e). Although the specific spin-coating protocols and processing windows of TDIN differ between these semiconductors (details provided in the experimental section of SI), the characteristic morphological progression was consistently reproduced through fine-tuning of the nucleation time as shown in Figures S10 and S11. Both semiconductors show an increase in μ_FET_ for films with a larger domain size. For (MeOPEA)_1.8_Cs_0.1_SnI_3.9_, the mobility improves from 0.3 cm^2 ^V^−1^ s^−1^ (SD) to 0.8 cm^2 ^V^−1^ s^−1^ (LD), reaching 2.8 cm^2 ^V^−1^ s^−1^ (UD). For TIPS-pentacene, despite intrinsically lower transport, μ_FET_ rises from 0.2 cm^2 ^V^−1^ s^−1^ to 0.5 cm^2 ^V^−1^ s^−1^ and finally to 0.7 cm^2 ^V^−1^ s^−1^ across the same morphological sequence. Device parameters are summarized in Tables S3 and S4, with corresponding output characteristics shown in Figs. S24b and S24c. The statistical analysis of multiple devices further confirms the improved and reproducible performance of UD-based FETs compared to LD and SD devices. The overall trends are consistent with those obtained in the case of (PEA)_2_SnI_4_. The recurrence of this transport behavior across chemically and structurally different semiconductors underscores the universality of the crystallization mechanism and of the impact on the electronic properties.

In conclusion, by integrating a TDIN strategy, stochastic nucleation becomes programmable, enabling direct control of the film morphology. The emergence of distinct morphological regimes is rationalized by concentration-dependent critical cluster radii in different environments. Based on this strategy, we establish a universal model that captures heterogeneous crystallization kinetics across materials and processing conditions. The model is broadly applicable, as validated for perovskite and organic semiconductors which exhibit distinct morphological regimes and crystallization behaviors. In-situ monitoring confirms the predictive accuracy of the model, showing that all nuclei, whether induced or spontaneous, grow via the same diffusion-controlled mechanism despite distinct domain-specific driving forces. The developed model offers a general strategy for understanding and controlling crystallization kinetics and defect formation relevant for both lateral and vertical device architectures. Since grain boundaries and defect distribution influence charge transport and recombination in both lateral and vertical devices, this universal strategy bears a broad potential for precise tailoring and elevating the performance of solution-processed optoelectronic devices beyond FET architectures.

## Methods

### Materials

All chemical reagents and solvents were used without further purification. Lead (II) iodide (PbI_2_, 99.99%), tin (II) iodide (SnI_2_, 99.999%), methylammonium iodide (MAI), cesium iodide (CsI), 6,13-bis(triisopropylsilylethynyl)pentacene (TIPS-pentacene), sodium dihydrogen phosphate (NaH_2_PO_4_), N,N-dimethylformamide (DMF, anhydrous, 99.8%), dimethyl sulfoxide (DMSO, anhydrous, ≥99.9%) toluene (anhydrous, ≥99.9%), chlorobenzene (CB, anhydrous, ≥99.9%) were purchased from Sigma-Aldrich. Tetrahydronaphthalin (Tetralin, anhydrous, ≥99.9%) was purchased from TCI chemical. Silicon wafers and ultraflat glasses were purchased from Ossila. 2-(2-Methoxyphenethyl)ammonium iodide (MeOPEAI) was purchased from Greatcellsolar Materials. Phenethylammonium iodide (PEAI) was purchased from Xi’an Polymer Baolaite Photoelectric Technology Corporation.

### Substrate preparation

Heavily p-doped bare Si/SiO_2_ wafers (1.5 × 2 cm) and ultraflat glass were used as substrates and cleaned in an ultrasonic bath by deionized water, acetone, and isopropyl alcohol and in the next step, blown dry with N_2_. The substrates were treated by oxygen plasma for 20 min to improve the hydrophilicity before used.

### Thin film preparation for TDIN

#### 2D (PEA)_2_SnI_4_ perovskite film

The precursor solutions were prepared in a stoichiometric ratio of PEAI and SnI_2_ 2:1 by dissolving PEAI and SnI_2_ in mixed DMF/DMSO solvent (1:1). The solution of defined concentrations (varied from 0.06 M to 0.12 M) was stirred overnight at 40 °C in a glove box and cooled to room temperature before deposition. The as-prepared fresh precursor solution was spin-coated at 2000 rpm with the defined times (varied from 32–52 s) on the glass for the morphology characterization or the silicon wafer for the device measurement, and annealed at two different temperatures, first at 60 °C around 10 min for crystallization followed by 100 °C for 10 min to remove the residual solvent.

#### 2D/3D (MeOPEA)_1.8_Cs_0.1_SnI_3.9_ perovskite film

The precursor solutions were prepared in a stoichiometric ratio of MeOPEAI, CsI and SnI_2_ 1.8:0.1:1 by dissolving them in mixed DMF/DMSO solvent (1:1). The 0.1 M solution was stirred overnight at 40 °C in a glove box and cooled to room temperature before deposition. The as-prepared fresh precursor solution was spin-coated at 2000 rpm with the defined times (varied from 50–64 s) on the glass for the morphology characterization or the silicon wafer for the device measurement, and annealed at two different temperatures, first at 60 °C around 10 min for crystallization followed by 100 °C for 10 min to remove the residual solvent.

#### 3D MAPbI_3_ perovskite film

The precursor solutions were prepared in a stoichiometric ratio of MAI and PbI_2_ 2:1 by dissolving them in mixed DMF/DMSO solvent (1:1). The 0.25 M solution was stirred overnight at 40 °C in a glove box and cooled to room temperature before deposition. The as-prepared fresh precursor solution was spin-coated at 2000 rpm with the time varied from 36–54 s on the glass substrate and annealed at 100 °C for 5 min.

#### Organic TIPS-pentacene semiconductor film

The precursor solutions were prepared by dissolving TIPS-pentacene in mixed solvent (45% toluene + 45% CB + 10% tetralin). The 10 mg/ml solution was stirred overnight at 60 °C in a glove box and cooled to room temperature before deposition. The as-prepared fresh precursor solution was spin-coated at defined speeds (varied from 2000 rpm to 2500 rpm) with defined times (from 3 s to 14 s) on the silicon wafer and annealed at 100 °C for 10 min.

#### Inorganic NaH_2_PO_4_ salt film

The precursor solutions were prepared by dissolving NaH_2_PO_4_ in deionized water at the concentration of 600 mg/ml. The as-prepared fresh precursor solution was spin-coated at 2000 rpm on the glass substrate and annealed at 100 °C for 10 min in air.

### Device fabrication and measurement

Bottom-gate top-contact configuration was employed for the FET devices. The 300 nm SiO_2_ layer of the silicon wafers was adopted as a dielectric layer. After film preparation, the source and drain electrodes were deposited with a thickness of 50 nm by gold thermal evaporation through a shadow mask (from Ossila), yielding transistor channels with dimensions of 80 × 1000 μm (length × width) for perovskite devices and 30 × 1000 μm for TIPS-pentacene devices. The device characterization was performed using a semiconductor parameter analyzer (Keithley 4200-SCS) of a low-temperature probe station. Both transfer and output characteristics were collected in pulse mode with on time of 0.05 s and off time of 1 s. Charge carrier mobility of the FETs were extracted from the following equation^44^:

4 $$\mu=\frac{2{L}_{{{{\rm{channel}}}}}}{{W}_{{{{\rm{channel}}}}}{C}_{{{{\rm{od}}}}}}{\left(\frac{\partial \sqrt{{I}_{{{{\rm{ds}}}}}}}{\partial {V}_{{{{\rm{g}}}}}}\right)}^{2}$$

where *L*_channel_, *W*_channel_, and *C*_od_ are the channel length and width and the unit capacitance of the oxide dielectric, respectively.

### Film characterization

Thin film morphology was characterized using a Leica POM and a Bruker Dimension Icon FS AFM in tapping mode at room temperature. GIWAXS measurements of (PEA)_2_SnI_4_ perovskite films were performed at the TU Dortmund DELTA Synchrotron. The beam size was 1.0 mm × 0.2 mm (width × height), and samples were irradiated just below the critical angle for total reflection with respect to the incoming X-ray beam (∼0.1°). For the film with unidirectional domains, the X-ray beam was positioned to overlap with the UD region, ensuring that the scattering signal is dominated by the aligned domains. The scattering intensity was detected on a two-dimensional image plate (MAR-345) with a pixel size of 150 μm (2300 × 2300 pixels) and the detector was placed 523 mm from the sample center. All X-ray scattering measurements were performed under vacuum (∼1 mbar) to reduce air scattering and beam damage to the sample. All data processing and analysis were performed using the software package Data squeeze. The CL was calculated for selected reflections from the Scherrer equation^45^. The interlayer distance was calculated from Bragg’s law: 2*dsin θ* = *nλ*, where *θ* = 5.46° and n = 2, respectively^46^.

### Movie recordings

Perovskite crystal growth was recorded in the glovebox using a WADEO microscope camera as MPEG-4/MOV (H.264, 8-bit RGB) movie with a resolution of 1920 × 1440 pixels and a frame rate of 30 fps. Movies were quantified using digital image processing techniques implemented in Python (OpenCV and NumPy-based workflow). Initially, movie footage of the crystallization process was used to isolate individual frames, which were converted to grayscale. To enhance the visibility and clarity of crystals within the frames, manual contrast and brightness adjustments were applied (linear rescaling of pixel intensities by a user-defined contrast factor and brightness offset), improving the contrast between the growing crystals and the background and enabling more robust boundary detection.

A region of interest (ROI) was then manually selected (user-defined coordinates) on the adjusted images to focus analysis on the specific area where crystal growth occurred. Within this ROI, intensity-based binary thresholding was applied using user-tuned threshold levels so that pixels corresponding to crystal features were separated from the background, generating binary images with crystal regions represented as foreground and the background suppressed. Continuous curves tracing crystal boundaries (contours) were then identified from the binary images using OpenCV contour detection. To minimize noise, very small detections were removed using a minimum feature-size (area) criterion, ensuring that only features consistent with crystal growth were retained.

For each remaining contour, a thickness metric was extracted and stored for subsequent analysis. To obtain spatially resolved growth information, the manually selected ROI was segmented into multiple smaller sub-regions (uniform slices across the ROI height), and the thresholding–contour detection–thickness calculation steps were repeated independently for each segment. Within each segment, the most representative feature was retained (the thickest detected line), reducing sensitivity to spurious edges and fragmented detections.

The resulting thickness values were further screened to prevent non-physical outliers from biasing statistics by excluding measurements that were much smaller or much larger than the median thickness within a frame (median-based outlier rejection using user-defined lower/upper multiplicative cutoffs). After filtering, for each frame and each ROI selection, summary statistics were computed, including the mean thickness and the standard deviation across segments (with the corresponding sample size).

Finally, the same movie was analyzed multiple times using different manually selected ROIs, and for each ROI the processing parameters (ROI placement, segmentation granularity, contrast/brightness adjustment, threshold levels, and outlier cutoffs) were fine-tuned to maximize signal-to-noise and provide the most stable quantification for that region. For presentation across repeated ROI selections, the reported central value at each time point corresponds to the mean of the per-ROI frame means. Error bars represent the standard error of the mean across ROI selections, calculated as the standard deviation of the per-ROI frame means divided by the number of ROI selections.

### Optical pump-terahertz (THz) probe spectroscopy

We build our THz spectrometer by a Ti:sapphire amplified pulsed laser system. The output of the laser features the following characteristics: the central wavelength is 800 nm; the pulse duration is around ~50 fs and the repetition rate is 1 kHz. In our study, the THz field is produced via the optical rectification effect in a ZnTe crystal. The bandwidth of the THz pulse is ~ 2 THz. The pump light is 400 nm, which is generated by second harmonic generation in a BBO crystal. The time-dependent product of photocarrier generation yield and mobility is calculated from

5 $$\frac{\Delta \sigma }{{N}_{{{{\rm{abs}}}},{{{\rm{film}}}}}}=\left(-\frac{{\varepsilon }_{0}c\,\left({n}_{1}+{n}_{2}\right)}{d}\cdot \frac{\triangle E}{E}\right)\cdot \frac{d}{{N}_{{{{\rm{abs}}}}}}=\varphi \mu \,$$

where ${N}_{{{{\rm{abs}}}}}$ is the absorbed sheet photon density, ${n}_{1}$ = 1.96 is the THz refractive index of fused silica, ${n}_{2}=1$ the refractive index of the air. ${N}_{{{{\rm{abs}}}}}$ is obtained as the product of the incident photon density and the absorption. ${N}_{{abs},{film}}$ is the number of absorbed photons in the unit thickness. Owing to the transient nature of the THz pulse (~ 1-2 ps duration), the THz field probes the local, intrinsic charge carrier mobility in the spatial range of ~10 nm. The pump-induced THz absorption -∆*E*/*E* is proportional to the photoconductivity ∆*σ*, which is related to the product of the photogenerated carrier density and mobility (-∆*E*/*E* ~∆*σ* = *e*∆(*N* · *μ*)). The product of photon-to-carrier efficiency (quantum yield *φ*) and mobility $(\varphi \mu )$ can be further inferred as a function of pump-probe delay time by applying the thin film approximation.

### Kelvin probe force microscopy (KPFM)

The contact potential difference (CPD) between the tip and sample for the perovskite films of FETs was measured with KPFM, using an Asylum Research MFP3D microscope from Oxford Instruments and a lock-in amplifier (HF2LI-MOD by Zürich Instruments). Since perovskites deteriorate under ambient conditions, the experiment was carried out in an argon glovebox. The bias voltages to the source and drain electrodes were provided by the auxiliary output of the lock-in amplifier, whereas the gate voltage was provided by an external battery. The cantilevers were conductively coated Pt-Ir (SCM-PIT-V2 by Bruker) with first eigenmode ω_0_ of ∼75 kHz and a spring constant of ∼3 N/m. The experiment was conducted with a scan rate of 0.2 Hz. For heterodyne KPFM, we excited the cantilever mechanically at *ω*_0_ and electrically at *ω*_1_-*ω*_0_, where *ω*_1_ is the second oscillating eigenmode of the cantilever. Then, by frequency mixing of the mechanical and the electrical oscillations of the cantilever, we generated a signal that coincided with the second resonance frequency and was therefore enhanced and used as input for the KPFM loop. In order to minimize the effect of stray electrostatic fields and topographical anomalies on the KPFM signal, KPFM was used in the heterodyne frequency-modulation variation^47^. After locating an area of the perovskite film between the source and drain electrodes with the microscope camera, the cantilever was positioned, and the KPFM experiment was conducted. In order to perform the experiments with an activated conductive channel, the drain and gate were biased at −9 V and −44 V, respectively, with respect to the grounded source to ensure an open channel according to the transfer and output characteristics.

### Transient absorption spectroscopy

Femtosecond transient absorption (fs-TA) spectra in the visible range were obtained using a Helios pump–probe setup (Ultrafast Systems) driven by an amplified 1030 nm laser system (Pharos, Light Conversion, 200 fs, 200 μJ, 1 kHz). The fundamental laser output was converted to 400 nm using an optical parametric amplifier (Orpheus-F, Light Conversion) and employed as the excitation pump. The visible broadband probe light was generated by directing a portion of the fundamental laser through an optical delay stage and focusing it onto a sapphire crystal. The 400 nm pump and broadband probe beams were spatially overlapped and focused onto the perovskite film samples. The transmitted probe light was collected and analyzed as a function of wavelength and pump–probe delay time.

### Time-resolved photoluminescence

TRPL was measured by a 4Piocs gated-iCCD camera (Stanford Computer Optics) with a Ti-sapphire laser (Coherent, Astrella, 5 mJ, 800 nm, 35 fs, 1 kHz). The excitation pulses at 400 nm were generated using an optical parametric amplifier (Orpheus, Light Conversion). The transient PL decays of the perovskite films were recorded with a low excitation power of 5 μW at a repetition rate of 1 kHz.

## Supplementary information

Supplementary Information

Description of Additional Supplementary Files

Supplementary Movie 1

Supplementary Movie 2

Supplementary Movie 3

Supplementary Movie 4

Transparent Peer Review file

## Source data

Source Data

## Data Availability

The data generated in this study have been deposited in the Edmond Research Data Repository under the DOI 10.17617/3.S597NE and are publicly available under a Creative Commons Attribution 4.0 International License (CC BY 4.0). Source data are provided with this paper.

## References

[1] Li, M. et al. Acceleration of radiative recombination for efficient perovskite LEDs. *Nature* **630**, 631–635 (2024). 10.1038/s41586-024-07460-7 PMC11186751 38811739

[2] He, J. et al. Solution-processed wafer-scale indium selenide semiconductor thin films with high mobilities. *Nat. Electron.* **8**, 244–253 (2025).

[3] Hu, S. et al. Steering perovskite precursor solutions for multijunction photovoltaics. *Nature* **639**, 93–101 (2025). 10.1038/s41586-024-08546-y PMC11882461 39715627

[4] Johnson, H. M. et al. Discovery of crystallizable organic semiconductors with machine learning. *J. Am. Chem. Soc.* **146**, 21583–21590 (2024). 10.1021/jacs.4c05245 PMC11311223 39051486

[5] Temiño, I. et al. Morphology and mobility as tools to control and unprecedentedly enhance X-ray sensitivity in organic thin-films. *Nat. Commun.* **11**, 2136 (2020). 10.1038/s41467-020-15974-7 PMC7195493 32358502

[6] Sun, G. & Tanaka, H. Surface-induced water crystallisation driven by precursors formed in negative pressure regions. *Nat. Commun.* **15**, 4–6 (2024). 10.1038/s41467-024-50188-1 PMC11282091 39060256

[7] Sosso, G. C. et al. Crystal nucleation in liquids: open questions and future challenges in molecular dynamics simulations. *Chem. Rev.* **116**, 7078–7116 (2016). 10.1021/acs.chemrev.5b00744 PMC4919765 27228560

[8] Sawatzki-Park, M., Wang, S. J., Kleemann, H. & Leo, K. Highly ordered small molecule organic semiconductor thin-films enabling complex, high-performance multi-junction devices. *Chem. Rev.* **123**, 8232–8250 (2023). 10.1021/acs.chemrev.2c00844 PMC10347425 37315945

[9] Wang, Z. et al. The coupling and competition of crystallization and phase separation, correlating thermodynamics and kinetics in OPV morphology and performances. *Nat. Commun.* **12**, 332 (2021). 10.1038/s41467-020-20515-3 PMC7804468 33436619

[10] Nie, W. et al. High-efficiency solution-processed perovskite solar cells with millimeter-scale grains. *Science* **347**, 522–525 (2015). 10.1126/science.aaa0472 25635093

[11] Min, H. et al. Hot-casting-assisted liquid additive engineering for efficient and stable perovskite solar cells. *Adv. Mater.* **34**, 2205309 (2022). 10.1002/adma.202205309 35841176

[12] Wang, S. et al. Modification of two-dimensional Tin-based perovskites by pentanoic acid for improved performance of field-effect transistors. *Small* **19**, 2207426 (2023). 10.1002/smll.202207426 36908090

[13] Zhu, H. et al. High-performance and reliable lead-free layered-perovskite transistors. *Adv. Mater.* **32**, 1–9 (2020). 10.1002/adma.202002717 32584475

[14] Whitehead, C. B., Saim, O. & Finke, R. G. LaMer’s 1950 model for particle formation of instantaneous nucleation and diffusion-controlled growth: a historical look at the model’s origins, assumptions, equations, and underlying sulfur sol formation kinetics data. *Chem. Mater.* **31**, 7116–7132 (2019).

[15] Wang, J. et al. Controlling the crystallization kinetics of lead-free tin halide perovskites for high performance green photovoltaics. *Adv. Energy Mater.* **11**, 1–18 (2021).

[16] Guan, Y. et al. Gypsum heterogenous nucleation pathways regulated by surface functional groups and hydrophobicity. *Nat. Commun.* **16**, 713 (2025). 10.1038/s41467-025-55993-w PMC11739488 39820035

[17] Long, Z., Wei, W. & Sun, C. Single particle fluorescence imaging of perovskite nanocrystal crystallization for illustrating coupled nucleation-and-growth. *Nat. Commun.* **16**, 5664 (2025). 10.1038/s41467-025-60826-x PMC12215261 40593586

[18] Naik, R. R. et al. Discovering equations that govern experimental materials stability under environmental stress using scientific machine learning. *npj Comput. Mater.* **8**, 72 (2022).

[19] Wang, Z. et al. Molecularly resolved mapping of heterogeneous ice nucleation and crystallization pathways using in-situ cryo-TEM. *Nat. Commun.* **16**, 7349 (2025). 10.1038/s41467-025-62900-w PMC12335531 40783494

[20] Yang, Y. et al. Suppressing vacancy defects and grain boundaries via Ostwald ripening for high-performance and stable perovskite solar cells. *Adv. Mater.* **32**, 1–7 (2020). 10.1002/adma.201904347 31880354

[21] Bu, T. et al. Modulating crystal growth of formamidinium–caesium perovskites for over 200 cm2 photovoltaic sub-modules. *Nat. Energy* **7**, 528–536 (2022).

[22] Zang, S. et al. Direct observation and control of non-classical crystallization pathways in binary colloidal systems. *Nat. Commun.* **16**, 3645 (2025). 10.1038/s41467-025-58959-0 PMC12003862 40240410

[23] Peng, Y. et al. In situ observation of coalescence of nuclei in colloidal crystal-crystal transitions. *Nat. Commun.* **14**, 4905 (2023). 10.1038/s41467-023-40627-w PMC10427646 37582924

[24] Bistervels, M. H. et al. Light-driven nucleation, growth, and patterning of biorelevant crystals using resonant near-infrared laser heating. *Nat. Commun.* **14**, 6350 (2023). 10.1038/s41467-023-42126-4 PMC10564937 37816757

[25] Lee, Y. H. et al. Multiple freezing–melting pathways of high-density ice through ice XXI phase at room temperature. *Nat. Mater.* **25**, 302–309 (2026) 10.1038/s41563-025-02364-x PMC12867761 41073658

[26] Lee, M. et al. Observing growth and interfacial dynamics of nanocrystalline ice in thin amorphous ice films. *Nat. Commun.* **15**, 908 (2024). 10.1038/s41467-024-45234-x PMC10827800 38291035

[27] Dong, H. et al. Decoupling nucleation and growth of crystal for DMSO-free self-assembly Tin Perovskite Films and their optoelectronic application. *Adv. Funct. Mater*. **35**, 2420593 (2025).

[28] Maschwitz, T. et al. How crystallization additives govern halide perovskite grain growth. *Nat. Commun.* **16**, 9894 (2025). 10.1038/s41467-025-65484-7 PMC12603034 41213992

[29] Liu, C., Cheng, Y. & Ge, Z. Understanding of perovskite crystal growth and film formation in scalable deposition processes. *Chem. Soc. Rev.* **49**, 1653–1687 (2020). 10.1039/c9cs00711c 32134426

[30] Frenkel, D. & Auer, S. Prediction of absolute crystal-nucleation rate in hard-sphere colloids. *Nature* **409**, 1020–1023 (2001). 10.1038/35059035 11234006

[31] Thanh, N. T. K., Maclean, N. & Mahiddine, S. Mechanisms of nucleation and growth of nanoparticles in solution. *Chem. Rev.* **114**, 7610–7630 (2014). 10.1021/cr400544s 25003956

[32] LaMer, V. K. & Dinegar, R. H. Theory, production and mechanism of formation of monodispersed hydrosols. *J. Am. Chem. Soc.* **72**, 4847–4854 (1950).

[33] Nicholson, S. T. et al. Characterization of mass transfer within the crystal-solution boundary layer of l-Alanine {120} faces using laser interferometry during growth and dissolution. *Cryst. Growth Des.* **23**, 2755–2769 (2023). 10.1021/acs.cgd.2c01541 PMC10080658 37038400

[34] Ball, R. C. & Somfai, E. Theory of diffusion controlled growth. *Phys. Rev. Lett.* **89**, 135503 (2002). 10.1103/PhysRevLett.89.135503 12225035

[35] Murphy, W. M., Oelkers, E. H. & Lichtner, P. C. Surface reaction versus diffusion control of mineral dissolution and growth rates in geochemical processes. *Chem. Geol.* **78**, 357–380 (1989).

[36] Seader, J. D. & Henley, E. J. *Crystallization, Desublimation, and Evaporation*. In Separation Process Principles, 2nd edn (John Wiley & Sons, Hoboken, 2006).

[37] Gao, Y. et al. Highly stable lead-free perovskite field-effect transistors incorporating linear Π-conjugated organic ligands. *J. Am. Chem. Soc.* **141**, 15577–15585 (2019). 10.1021/jacs.9b06276 31525969

[38] Wang, S. et al. Odd–even alkyl chain effects on the structure and charge carrier transport of two-dimensional Sn-based perovskite semiconductors. *J. Am. Chem. Soc.* **146**, 19128–19136 (2024). 10.1021/jacs.4c03936 PMC11258789 38953716

[39] Tsai, H. et al. High-efficiency two-dimensional Ruddlesden-Popper perovskite solar cells. *Nature* **536**, 312–317 (2016). 10.1038/nature18306 27383783

[40] Qin, M., Chan, P. F. & Lu, X. A systematic review of metal halide perovskite crystallization and film formation mechanism unveiled by in situ GIWAXS. *Adv. Mater.* **33**, 1–28 (2021). 10.1002/adma.202105290 34605066

[41] Son, D. Y. et al. Self-formed grain boundary healing layer for highly efficient CH3NH3PbI3 perovskite solar cells. *Nat. Energy* **1**, 1–8 (2016).

[42] Niu, T. et al. Stable high-performance perovskite solar cells via grain boundary passivation. *Adv. Mater.* **30**, 1–11 (2018). 10.1002/adma.201706576 29527750

[43] Jiménez, J. M. et al. Modification of charge trapping at particle/particle interfaces by electrochemical hydrogen doping of nanocrystalline TiO_2_. *J. Am. Chem. Soc.* **138**, 15956–15964 (2016). 10.1021/jacs.6b08636 PMC5193466 27960341

[44] Choi, H. H. et al. Critical assessment of charge mobility extraction in FETs. *Nat. Mater.* **17**, 2–7 (2017). 10.1038/nmat5035 29255225

[45] Holzwarth, U. & Gibson, N. The Scherrer equation versus the Debye-Scherrer equation. *Nat. Nanotechnol.* **6**, 534 (2011). 10.1038/nnano.2011.145 21873991

[46] Richel, A., Johnson, N. P. & McComb, D. W. Observation of Bragg reflection in photonic crystals synthesized from air spheres in a titania matrix. *Appl. Phys. Lett.* **76**, 1816–1818 (2000).

[47] Axt, A. et al. Know your full potential: Quantitative Kelvinprobe force microscopy on nanoscale electrical devices. *Beilstein J. Nanotechnol.* **9**, 1809–1919 (2018). 10.3762/bjnano.9.172 PMC6009372 29977714

