# Supplementary material for: Universal crystallization kinetics of solution-processed semiconductors
Source: Nat Commun. 2026 Oct 14;17:10785. doi: 10.1038/s41467-026-78354-7 (PMC13669315; doi:10.1038/s41467-026-78354-7)
Supplement: Supplementary file 1 [file 41467_2026_78354_MOESM7_ESM.pdf]

nature portfolio

Peer Review File

Universal Crystallization Kinetics of Solution-Processed Semiconductors

Corresponding Author: Professor Tomasz Marszalek

This file contains all reviewer reports in order by version, followed by all author rebuttals in order by version.

Version 1:

Reviewer comments:

Reviewer #1

(Remarks to the Author)
The manuscript titled "Universal Crystallization Kinetics of Solution-Processed Semiconductors" shows an interesting phenomenon regarding the crystallization dynamics, particularly in terms of the control of nucleation density and its spatial environment. Although the crystallization kinetics theory is textbook-level concept, the authors design an elaborate spatial difference in the film to realize three distinct morphologies. To provide a better understanding of this technology and its application for the audience, several important comments should be addressed:
1. The characterization of the crystallization kinetics in this work appears to be somewhat limited. What evidence supports that the growth process occurs only during the annealing stage, while only nucleation takes place during the spin-coating stage?
2. What evidence supports whether the crystallization initiates from the top surface or the bottom interface?
3. In Fig. S8, why does the sample with a higher concentration exhibit a delayed onset of unidirectional growth? This observation appears somewhat counterintuitive.
4. How does the experimental procedure ensure that the three assumptions stated in line 250 are valid?
5. Material defects can significantly affect the optoelectronic performance of the devices. It is recommended to provide additional characterization of defect states for materials prepared via different growth modes.
6. The details of the FET devices are insufficient. How are SD, LD, and UD films implemented in the device architecture? How is the stability of the devices prepared via different growth modes?
7. The device performance should be presented in a statistical manner to demonstrate the reliability of the conclusions.
8. How can the horizontally grown crystallization mode be applied to devices such as solar cells that require a vertical structure?

Reviewer #2

(Remarks to the Author)
This manuscript introduces a time-defined induced nucleation strategy to separate the nucleation and growth times that enables semiconductors to crystallize at distinct stages during solution processing. From this study, the authors come up with a model to capture the essential features of semiconductor nucleation and growth in solution across different processing conditions. The work is of current interest and the approach is neat with some potentially valuable results. However, the distinct aspects of the developed model are not clearly articulated, especially against the benchmark models of LaMer , Avrami, etc.--it seems to be still mainly based on classical nucleation theory, which needs to be justified on its own, leaving the induced nucleation only an experimental design innovation. Also, the wider relevance and presentation of the manuscript need to be much improved to warrant the publication. More detailed comments are given below.

1) The demonstrated semiconductor systems are mainly low-dimensional perovskites, why not 3-dimensional perovskites that are actually the workhorse of the now most promising perovskite solar cells? This inclusion will give very forceful support to the model.

2) There should be discussion how the model can be practically used to design processing conditions of semiconductor devices, especially with a large size.

3) Different nucleation mechanisms are involved, both induced and spontaneous. Would it make analysis more difficult since they interfere with each other and we nee distinguish between the two? Is it possible to only allow induced one since the spontaneous one has higher energy cost?

4) The films from the strategy are quite inhomogeneous globally, making it unsuitable for devices. Even for characterizations such as GIWAX, mobility, etc., they are referred to specific parts of the films?

5) Length L's are variously used, but they do not seem to be defined clearly. While we may see L is represented by areas in concentration vs time plots, they are not clearly illustrated in the actual film images and how we get the L values from the images. By the way, in Fig M1(SI), L is also shown, is that the same L we are talking about?

6) There are also many other parameters such as Css, many different t's, etc., which are not clearly defined as well. For example, what is meant by supersaturation for Css? quite confusing.

7) Many equations have problems, for example, Eq S9, why plus sign in the denominator? How can one go from Eq S11 to S12? It is said that one can get max t if t's are zero in Eq S12, but if then, the l;eft side becomes all zero.

8) SI, Fig M2: only 3 points, enough to make a case? Also why only for UD? Actually, I wonder why do we have SD, UD, LD instead of SD, MD (medium domain), LD? It seems UD need to be explained more clearly.

9) Nucleation is induced at a specific location, so diffusion should play a very important role. But the model seems to have no information on diffusion parameters.

10) SI Eq 52: There are also problems here, such as photoconductivity change (delta), film (not flim).

11) Line 427: mobility inccrease with morphology? (change with morphology?) Why UD morphology is the best?

12) Fig 3abcd: which is LD, UD, LD? They should be more clearly labeled.

13) Check the whole manuscript and SI, e.g., SI, line 71: time interval, ...

Reviewer #3

(Remarks to the Author)
The work by Ling et al. reported an innovative method to decouple the nucleation and crystal growth process in a wide range of solution-processed semiconductor thin films. Time-defined induced nucleation (TDIN) is achieved by scratching the wet film within a specific time window after spin coating, during which the supersaturation is high enough for scratching to induce heterogeneous nucleation yet low enough to avoid homogenous nucleation in the bulk. The subsequent thermal annealing then allows the author to draw a direct correlation between crystal size and annealing time, which can be described by a diffusion-based model. I think this work provides theoretical insights for optimising the fabrication procedure of solution-processed optoelectronic thin films, especially for metal-halide perovskites, where the time interval between spin coating of wet film and the onset of thermal annealing is known to critically determine the performance of perovskite PVs. Induced crystal growth perpendicular to the scratch line is also beneficial for solution-processed FETs, where crystal alignment along the channel length direction can help reduce charge trapping at grain boundaries, as demonstrated by the authors.
Therefore, I recommend that this paper be published in Nature Communications after addressing the following concerns
1. In Figures 1f and g, the author demonstrates the TDIN strategy on five different material systems. While the universal LD-UD-SD evolution is clearly seen in 2D perovskite, 2D/2D perovskite, and 3D perovskite, the trend becomes less obvious in TIPS-pentacene and inorganic salts. In the latter two cases, clear large domains can still be identified in the SD figures, which requires further clarification.
2. In this paper, the author considered heterogeneous nucleation initiated at the tip, the solution-air interface, and homogenous nucleation within the solution bulk. In practice, heterogeneous nucleation can also initiate at the solution-substrate interface, which should be taken into account, given that a diverse range of substrates with different surface energies has been used.
3. Since the author mainly studied the heterogeneous nucleation (from scratch tip) and unidirectional growth of cylindrical crystals, the plots in Figures 1c and 1d should be derived for cylindrical rather than spherical nuclei.
4. Since the crystal growth induced by scratching is highly anisotropic, the author should specify the alignment condition employed for GIWAXS measurements. e.g. whether the direction of the incident X-ray is parallel or perpendicular to the scratch line. Considering the length of the crystal grown from scratch (a few hundred μm) is very similar to the width of the X-ray beam (1mm), I think that the incident X-ray beam must be parallel and overlap with the scratch to ensure that the crystals sampled by the X-ray are indeed the UDs rather than the LDs around it.
5. The author should also report the anisotropy in FET mobilities when the channel is parallel or perpendicular to the direction of UD growth.

Reviewer #4

(Remarks to the Author)
A strategy is introduced to separate crystal crystallization and growth in this work, and a time-defined induced nucleation

model is constructed to achieve quantitative analysis of heterogeneous crystals and tailor the crystallinity of thin film based on solution-processed. This article effectively combines theoretical calculations with experimental results, and demonstrates the universality of the proposed model by replacing different material systems. However, this work employs relatively limited microscopic characterization techniques for analyzing crystallization morphology. Commonly used methods such as SEM and TEM were not utilized, which constrains the depth and comprehensiveness of the experimental results. Overall, this is a good article that provides a relatively universal and quantitative model for heterogeneous nucleation, which is of great help for future regulation of thin film growth. In conclusion, I recommend that this article be published after minor revisions.

1. This work mainly introduces three heterogeneous nucleation regions (bulk, cusp and interface(gas/liquid)), but besides these regions, the wet film and substrate are also a heterojunction interface. Why is this interface not discussed here? Furthermore, has there been any attempt to use different types of substrates for experiments (such as rough substrates and relatively smooth substrates)? If possible, please provide relevant experimental and theoretical analysis data, thank you!
2. This work mainly discusses one-step spin-coating. However, in the actual preparation process, multi-step spin-coating is also a very common spin-coating mode. If possible, please supplement the relevant experimental data and theoretical analysis of multi-step spin-coating, thank you!
3. The characterization of grains in this article is too macroscopic. In addition to macroscopic morphology, probing the microscopic properties of grains is highly valuable. For example, acquiring surface morphology and cross-sectional images of the films using SEM would provide important insights. If possible, please supplement relevant experimental data, thank you!
4. Many of the contents in this work involve theoretical calculations and the application of various computational models. If possible, please briefly explain the role and applicable conditions of the model using the corresponding calculations. This will greatly help readers understand the content of the article, thank you!

Version 2:

Reviewer comments:

Reviewer #1

(Remarks to the Author)
The authors have substantially improved the manuscript. This work presents a reasonable and readily understandable model for crystallization modulation. Although the current implementation based on mechanical scratching is still far from practical application, the results highlight the importance of considering anisotropic crystallization in device design. I therefore recommend acceptance of the manuscript. However, before publication, I still have several technical questions, most of which are related to my previous comments.
1. For defect assessment, time-resolved photoluminescence (TRPL) measurements would be more appropriate, as they can provide information on defect-related non-radiative recombination. When performing both transient absorption (TA) and TRPL measurements, please ensure that the laser is focused on the appropriate region and that the excitation intensity is sufficiently low to avoid significant Auger recombination. Several parallel scratch lines could be introduced to enlarge the UD region, and/or the LD region could be removed by laser scribing.
2. In Fig. S20, the FET channel is oriented parallel to the grain orientation of the UD perovskite. Could the authors fabricate a device with the channel oriented perpendicular to the grain direction to further demonstrate the anisotropic transport properties of the UD perovskite? Two parallel scratch lines may be used to obtain a sufficiently long UD region. If it is technically difficult to produce a region long enough for device fabrication, the authors may instead provide what they have in their response to this comment.

Reviewer #2

(Remarks to the Author)
The files are for the first round of revisions, and I did not find the files for addressing specific to my latest comments.

Reviewer #3

(Remarks to the Author)
The authors have addressed all my comments.

Reviewer #4

(Remarks to the Author)
Thank you very much for the author's efforts! After revision, the reliability and overall logical chain of the article have become more reliable and convincing. My recommendation is to accept, but there are some small suggestions as follows:
1.Thank you very much for the author's additional description of liquid/substrate interface nucleation. However, I noticed that there is still only one curve for the interface in Fig.1C. Does the author mean that theoretically the situation of the two interfaces(liquid/air and liquid/substrate) is the same? If so, please provide corresponding theoretical and experimental results to prove this conclusion。
2.The additional SEM characterization is appreciated. However, the magnification of the provided SEM images is still insufficient to clearly resolve the microstructural features of the grains. Furthermore, the crystallographic orientation and facet

characteristics of the grains are also critical. If possible, the authors are encouraged to provide additional crystallographic characterization, such as EBSD, to reveal the grain orientation.

Version 3:

Reviewer comments:

Reviewer #1

(Remarks to the Author)
The authors have satisfactorily addressed my concerns, and I have no further questions.

Reviewer #4

(Remarks to the Author)
Thank you very much to the authors for their careful and thorough revisions. The manuscript has been significantly improved, and the overall logic, clarity, and reliability of the presented arguments are now much stronger and more convincing. In my opinion, the authors have adequately addressed the concerns I raised during the review process. I therefore recommend acceptance of the manuscript in its present form.

Open Access This Peer Review File is licensed under a Creative Commons Attribution 4.0 International License, which permits use, sharing, adaptation, distribution and reproduction in any medium or format, as long as you give appropriate credit to the original author(s) and the source, provide a link to the Creative Commons license, and indicate if changes were made.
In cases where reviewers are anonymous, credit should be given to 'Anonymous Referee' and the source.
The images or other third party material in this Peer Review File are included in the article’s Creative Commons license, unless indicated otherwise in a credit line to the material. If material is not included in the article’s Creative Commons license and your intended use is not permitted by statutory regulation or exceeds the permitted use, you will need to obtain permission directly from the copyright holder.
To view a copy of this license, visit https://creativecommons.org/licenses/by/4.0/

MAX PLANCK INSTITUTE
FOR POLYMER RESEARCH

Division Molecular Electronics

Prof. Dr. hab. Tomasz Marszalek
Group Leader

marszalek@mpip-mainz.mpg.de

+49-(0) 6131 379 721
+49-(0) 6131 379 320

Point-by-point response to the reviewers' comments

Reviewer: 1

Comment: The authors have substantially improved the manuscript. This work presents a reasonable and readily understandable model for crystallization modulation. Although the current implementation based on mechanical scratching is still far from practical application, the results highlight the importance of considering anisotropic crystallization in device design. I therefore recommend acceptance of the manuscript. However, before publication, I still have several technical questions, most of which are related to my previous comments.

For defect assessment, time-resolved photoluminescence (TRPL) measurements would be more appropriate, as they can provide information on defect-related non-radiative recombination. When performing both transient absorption (TA) and TRPL measurements, please ensure that the laser is focused on the appropriate region and that the excitation intensity is sufficiently low to avoid significant Auger recombination. Several parallel scratch lines could be introduced to enlarge the UD region, and/or the LD region could be removed by laser scribing.

Answer: We thank the reviewer for this valuable suggestion. Following the reviewer's recommendation, we have performed additional time-resolved photoluminescence (TRPL) measurements on the LD, UD and SD regions based on $(PEA)_2SnI_4$ to further evaluate defect-related non-radiative recombination. The corresponding results have been added to Figure S23. During the TRPL measurements, the laser spot was carefully positioned within the target region. A sufficiently low excitation was used to minimize carrier-density-dependent effects such as Auger recombination.

The TRPL decay curves show that both LD and UD exhibit significantly slower decay than SD, indicating reduced defect-assisted non-radiative recombination compared with the SD film. The decay dynamics of the LD and UD regions are similar, with slightly smaller decline for the LD morphology following the trend of the photoconductivity dynamics in Figure S21. This confirms that the enhanced FET performance of the UD films cannot be explained solely by a reduction in defect density. Instead, the superior device performance mainly originates from the continuous transport pathways due to the aligned domains with reduced density of boundaries.

Page 23: "Additional time-resolved photoluminescence measurements in **Figure S23** reveal similar decay dynamics for the LD and UD films, whereas the SD film exhibits a faster decay, indicating enhanced defect-assisted non-radiative recombination in the SD film. This observation is consistent with the photoconductivity dynamics shown in **Figure S21**, suggesting that the superior FET performance mainly arises from continuous transport pathways along the aligned domains with reduced density of boundaries rather than lower defect density."

Page 19 (SI): "**Time-resolved Photoluminescence** TRPL was measured by a 4Piocs gated-iCCD camera (Stanford Computer Optics) with a Ti-sapphire laser (Coherent, Astrella, 5 mJ, 800 nm, 35fs, 1 kHz). The excitation pulses at 400 nm were generated using an optical parametric amplifier (Orpheus, Light Conversion). The transient PL decays of the perovskite films were recorded with a low excitation power of 5 μW at a repetition rate of 1 kHz."

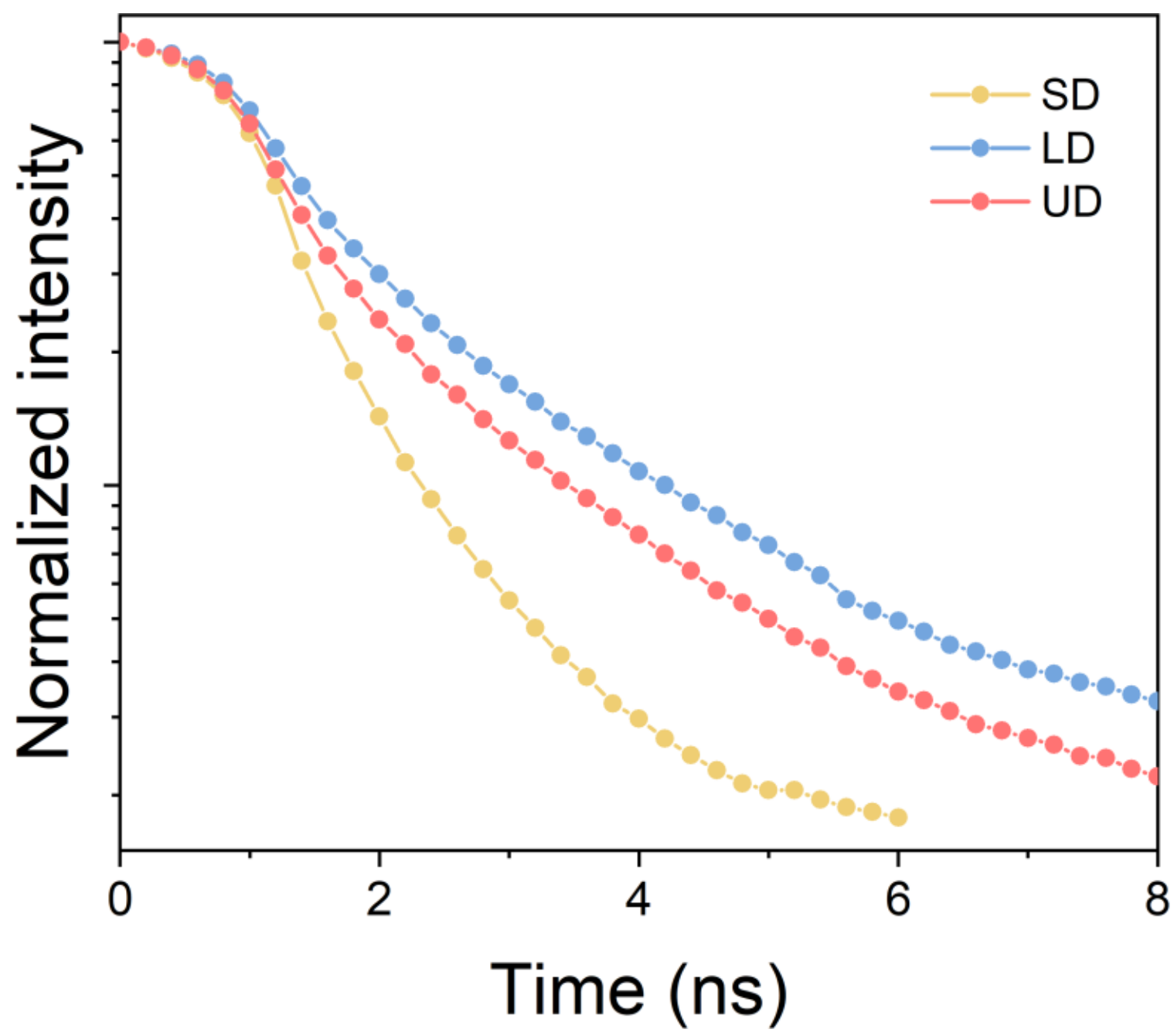

Figure S23. Normalized time-resolved photoluminescence decay curves of the SD, LD, and UD films measured under identical excitation conditions.

Comment: In Fig. S20, the FET channel is oriented parallel to the grain orientation of the UD perovskite. Could the authors fabricate a device with the channel oriented perpendicular to the grain direction to further demonstrate the anisotropic transport properties of the UD perovskite? Two parallel scratch lines may be used to obtain a sufficiently long UD region. If it is technically difficult to produce a region long enough for device fabrication, the authors may instead provide what they have in their response to this comment.

Answer: We thank the reviewer for this valuable suggestion. We carefully considered this device geometry and evaluated whether it could be realized using two parallel scratch lines.

To examine this possibility, we prepared samples with two parallel scratch lines and verified the film morphology by optical microscopy, as shown in Figure R1. Instead of extending a single continuous UD region, two parallel scratches produce two independently growing UD regions that impinge upon each other and form a collision boundary. In our system, the maximum length of the UD region for $(PEA)_2SnI_4$ is about 400 µm, while the channel width of the FET devices is 1000 µm. Therefore, a device with the channel perpendicular to the UD growth direction would inevitably include either the scratch line or the collision boundary within the channel. In this case, the measured transport would be strongly influenced by these structural discontinuities, making it difficult to distinguish the intrinsic transport perpendicular to the UD growth direction from scattering and trapping at the scratch or domain boundary.

For this reason, we believe that such a device would not provide a reliable evaluation of the anisotropic transport properties of the UD film and could lead to misleading conclusions. Nevertheless, we agree that direct measurement of in-plane anisotropic transport in a sufficiently large continuous UD domain would be interesting and will be an important direction for future work.

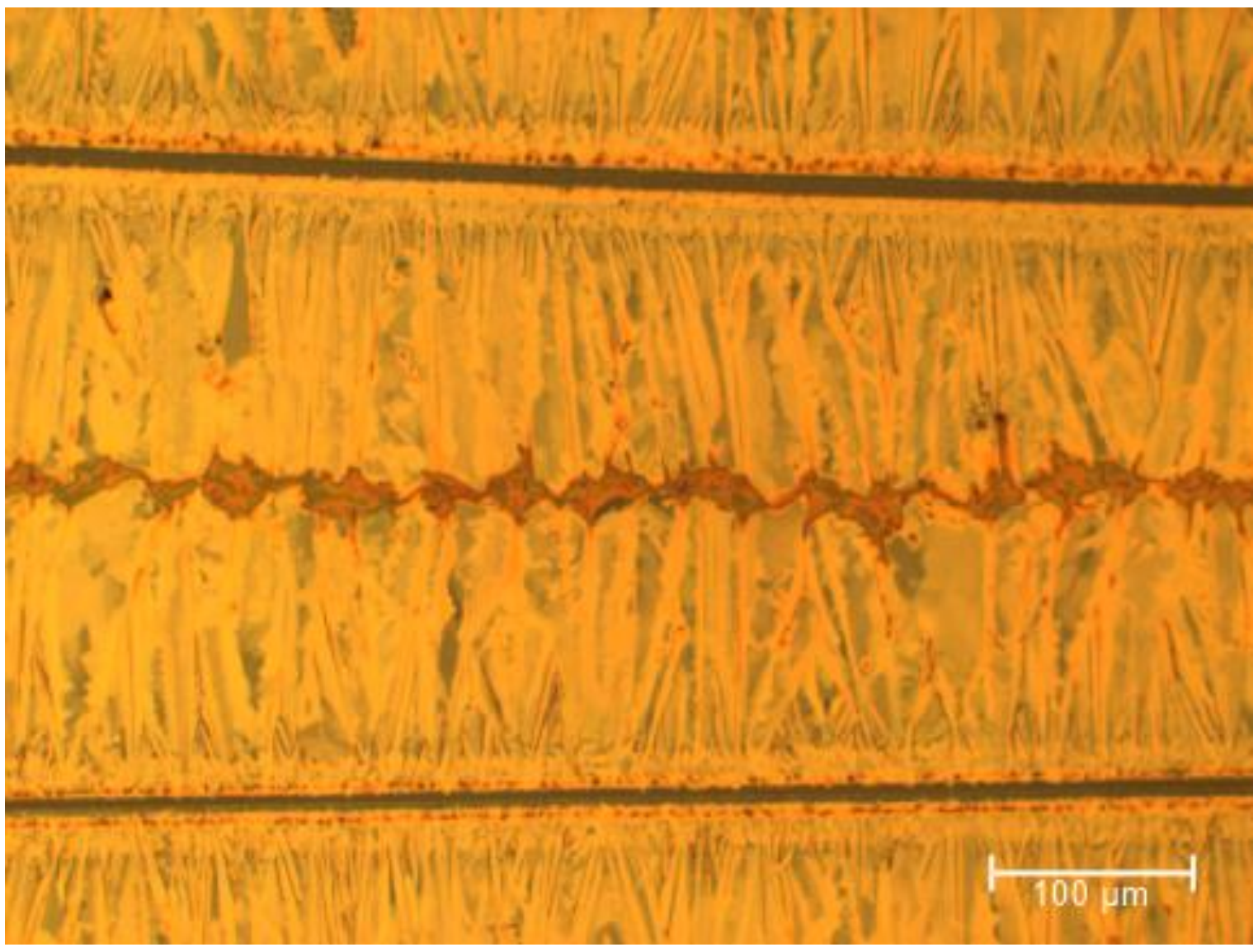

Figure R1. Optical microscopy image of the film prepared with two scratch lines. Two independently grown UD regions meet in the middle, forming a collision boundary.

Reviewer: 4

Comment: Thank you very much for the author's efforts! After revision, the reliability and overall logical chain of the article have become more reliable and convincing. My recommendation is to accept, but there are some small suggestions as follows:

Thank you very much for the author's additional description of liquid/substrate interface nucleation. However, I noticed that there is still only one curve for the interface in Fig.1C. Does the author mean that theoretically the situation of the two interfaces (liquid/air and liquid/substrate) is the same? If so, please provide corresponding theoretical and experimental results to prove this conclusion.

Answer: We thank the reviewer for this insightful comment. We would like to clarify that the single "Interface" curve in Figure 1C is not intended to imply that nucleation at the liquid/air and liquid/substrate interfaces is theoretically identical. Instead, it represents heterogeneous interfacial nucleation in a general sense. Since the nucleation barrier depends on the specific material system and interfacial properties, the liquid/air and liquid/substrate interfaces may exhibit different barriers. To avoid this possible misunderstanding, we have revised the figure caption to explicitly clarify this point.

Page 6: "*The interface curve represents heterogeneous interfacial nucleation in a general sense and is not intended to distinguish between the liquid/air and liquid/substrate interfaces, whose nucleation barriers may differ depending on the specific material system and interfacial properties.*"

Comment: The additional SEM characterization is appreciated. However, the magnification of the provided SEM images is still insufficient to clearly resolve the microstructural features of the grains. Furthermore, the crystallographic orientation and facet characteristics of the grains are also critical. If possible, the authors are encouraged to provide additional crystallographic characterization, such as EBSD, to reveal the grain orientation.

Answer: We thank the reviewer for this valuable suggestion. We agree that higher-magnification SEM images and crystallographic orientation analysis would provide additional information on the grain structure. The SEM images included in the revised manuscript were acquired at the highest magnification that could be achieved without introducing noticeable electron-beam damage. Due to the high beam sensitivity of the Sn-based halide perovskite films, further increasing the magnification or electron dose resulted in visible cracking and degradation of the film, preventing reliable imaging of the original microstructure.

We also carefully considered EBSD measurements. However, EBSD characterization of beam-sensitive halide perovskites remains technically challenging, as electron-beam exposure induces structural degradation. To characterize the crystallographic orientation towards the surface, we refer to the GIWAXS results presented in the manuscript, which reveal a highly arranged crystal structure of the UD films. As discussed in the manuscript, the UD film exhibits a larger coherence length, narrower and split in-plane diffraction features, and highly ordered molecular packing, indicating enhanced crystallinity, enlarged domains, and similar in-plane

crystal orientations compared with the SD and LD films. While additional crystallographic characterization would certainly be valuable, the primary focus of this work is the development and experimental validation of the proposed crystallization model. The structural characterization presented here provides complementary evidence supporting the proposed model and the associated device performance.

Point-by-point response to the reviewers' comments

Reviewer: 1

Comment: The manuscript titled "Universal Crystallization Kinetics of Solution-Processed Semiconductors" shows an interesting phenomenon regarding the crystallization dynamics, particularly in terms of the control of nucleation density and its spatial environment. Although the crystallization kinetics theory is textbook-level concept, the authors design an elaborate spatial difference in the film to realize three distinct morphologies. To provide a better understanding of this technology and its application for the audience, several important comments should be addressed:

Answer: We thank the reviewer for the careful assessment of our work and for recognizing the interest of the crystallization kinetics and morphology control demonstrated in this study.

We agree that the fundamental concepts of crystallization kinetics are well established, such as Lamer curve or Avrami model (References [14-19] in the manuscript). However, the key contribution of this work lies in extending these concepts toward a deterministic and quantitative framework that enables the separation of nucleation and growth in space and time. In particular, the time-defined induced nucleation (TDIN) strategy allows us to control individual nucleation events and directly correlate them with subsequent domain growth.

This approach differs from classical models, which typically describe ensemble-averaged behavior (e.g. nucleation density or crystallization kinetics for overall films), whereas our framework resolves the evolution of individual domains and provides a unified description across different materials and processing conditions. The underlying crystallization concepts are classical, but the present work extends them from ensemble-averaged descriptions to a deterministic, time-resolved framework for individual heterogeneous nucleation events and subsequent domain growth in solution-processed thin films.

In response to the reviewer's comment, we have further clarified this aspect in the revised manuscript to stronger highlight the conceptual advance and its relevance for understanding and controlling crystallization in solution-processed thin films.

Page 4: "While classical crystallization theories describe an ensemble-averaged behavior, the present work establishes a model to resolve and control the evolution of individual domains under heterogeneous conditions."

Our point-by-point responses are provided below.

Comment: (R1.1) The characterization of the crystallization kinetics in this work appears to be somewhat limited. What evidence supports that the growth process occurs only during the annealing stage, while only nucleation takes place during the spin-coating stage?

Answer: We thank the reviewer for this important comment regarding the temporal relationship between nucleation and growth. We would like to clarify that the present work does not assume that nucleation occurs exclusively during spin-coating and growth only during annealing. Instead, both nucleation and growth are governed by the evolution of solute concentration, and may occur during spin-coating, annealing, or both, depending on the processing conditions.

Specifically, three distinct crystallization regimes are identified in the graph of Figure 1e based on the concentration-dependent nucleation condition at mentioned in the manuscript at page 9:

(i) In the blue regime ($C < C_i$), nucleation does not occur during spin-coating due to insufficient supersaturation, and both nucleation and subsequent growth take place during later annealing stage when the concentration increases, which leads to LD formation.

(ii) In the red regime ($C_i \leqslant C < C_{ss}$), nucleation is mechanically induced after a defined time of spin-coating (via scratching), while subsequent UD growth proceeds as the concentration continues to increase during annealing.

(iii) In the yellow regime ($C \geqslant C_{ss}$), spontaneous nucleation occurs rapidly once the concentration exceeds the bulk nucleation threshold, leading to concurrent nucleation and growth of SD within the film during the spin-coating stage.

Therefore, the TDIN framework does not impose a fixed separation between spin-coating and annealing stages, but instead enables a controllable, concentration-dependent separation of nucleation and growth.

To avoid possible misunderstanding, we have revised the manuscript to explicitly emphasize that nucleation and growth are determined by the supersaturation rather than by specific processing stages.

Page 9: "... controlling the supersaturation via ..."

Page 9: "Importantly, nucleation and growth are not intrinsically tied to specific processing stages, i.e. spin-coating or annealing, but are governed by the evolution of solute concentration. Depending on the supersaturation trajectory, nucleation and subsequent growth may occur during spin-coating, annealing, or both. The three regimes described above represent distinct concentration-dependent crystallization pathways rather than stage-specific processes."

<u>Comment:</u> (R1.2) What evidence supports whether the crystallization initiates from the top surface or the bottom interface?

<u>Answer:</u> In the original manuscript, the discussion primarily focused on heterogeneous nucleation at the liquid/air interface (top surface) to illustrate the mechanism, which may have given the impression that a specific nucleation location was assumed.

We would like to clarify that the model is not restricted to a particular interface. In practice, nucleation in solution-processed thin films may be initiated at different locations, including the liquid/air interface (top surface) or the liquid/substrate interface (bottom interface), depending on the relative interfacial energies and material systems. In our experiments, different substrates (e.g. silicon, glass, and quartz) and different materials were employed, and consistent LD-UD-SD morphological evolution was observed across all cases. Importantly, the nucleation condition in all cases is governed by the same fundamental criterion based on the relation between cluster size and critical radius. Furthermore, the mechanically induced nucleation at the cusp region provides the lowest nucleation barrier and dominates the induced nucleation process for UD. Therefore, identifying a specific nucleation location (top versus bottom interface) is not required for establishing the validity of the proposed model, which is governed by the concentration-dependent nucleation condition. As a result, the overall crystallization pathway and the applicability of the model remain unchanged.

We have clarified this point in the revised manuscript adding a modified illustration of Figure 1b.

Figure 1b:

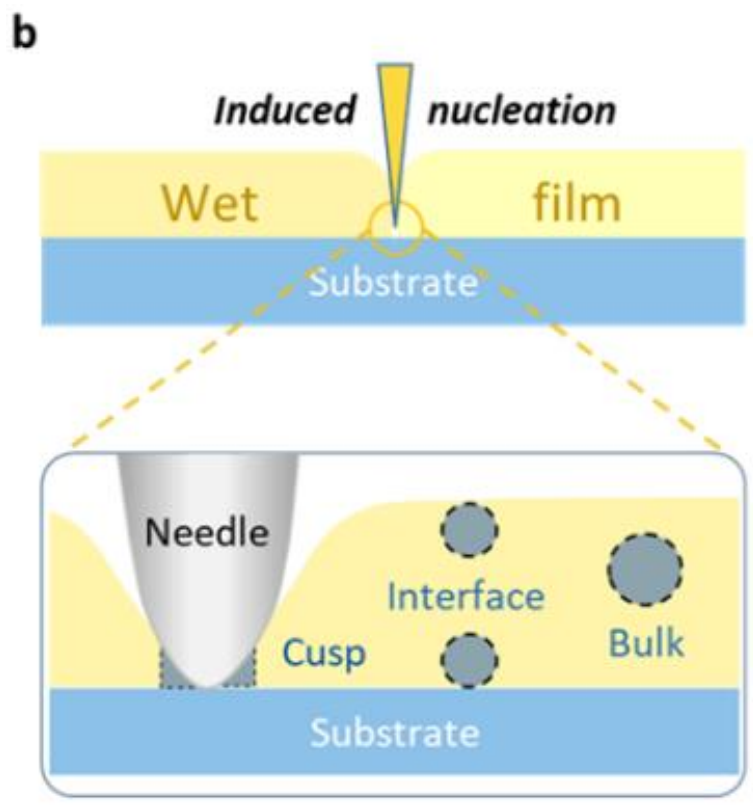

Page 6: "... or liquid/substrate ..."

Page 7: "The nucleation sites can be located in the bulk (homogeneous nucleation) the liquid/air or liquid/substrate interface (heterogeneous nucleation), where nucleation may preferentially occur depending on the material system"

Page 9: "... or liquid/substrate ..."

Comment: (R1.3) In Fig. S8, why does the sample with a higher concentration exhibit a delayed onset of unidirectional growth? This observation appears somewhat counterintuitive.

Answer: We agree that this observation may appear counterintuitive if only the initial precursor concentration is considered in Figure S8 (now updated to Figure S15 and Figure S9). The onset of UD growth is governed by the time required for the wet film to reach the critical concentration for the induced nucleation (C_i), rather than by the initial concentration alone.

The films prepared from different initial concentrations exhibit different concentration-evolution rates because their initial wet film thicknesses are different. In particular, a solution of lower concentration forms a thinner wet film, and therefore a larger relative reduction in wet film volume occurs during solvent evaporation which leads to a faster concentration increase. As a result, the lower concentration film may reach C_i earlier, giving rise to an earlier onset of the UD growth. This behavior is consistent with previous studies on spin-coated films, where films of lower concentration reached supersaturation conditions earlier despite (Reference [5] updated in SI: Spies et al., Adv. Electron. Mater. 2025).

We have clarified this point in the revised Supporting Information.

Page 25 (SI): "In Figure S9, the delayed onset of UD growth at higher precursor concentration may appear counterintuitive if only the initial concentration is considered. However, the onset of the induced nucleation is governed by the time required for the wet film to reach C_i, which depends on the dynamic evolution of the concentration during solvent evaporation.

Spin-coating a solution with a lower precursor concentration typically yields a thinner wet film in which the solvent evaporation results in a larger relative reduction in volume. Consequently, the solute concentration increases more rapidly, allowing the wet film to reach C_i at an earlier time.[5] In contrast, a solution with a higher precursor concentration forms a thicker wet film, leading to a slower relative increase in concentration and a delayed onset of the UD growth."

Reference SI [5]: Spies, M. *et al.* Solvated PbI2 Clusters Preceding the Crystallization of Lead Halide Perovskites–a UV/VIS In Situ Study. *Adv. Electron. Mater.* **11**, 2500060 (2025).

Comment: (R1.4) How does the experimental procedure ensure that the three assumptions stated in line 250 are valid?

Answer: The three assumptions are supported by both the experimental design and direct observations, as detailed below:

(1) Infinite substrate assumption: The dimension of the substrate is orders of magnitude larger than the characteristic size of the unidirectional domains (typically a few hundred micrometers). Therefore, the substrate can be reasonably treated as effectively infinite for the purpose of modelling the UD growth.

(2) Termination of UD growth at C_{ss}: This assumption is based on the classical nucleation theory, in which reaching the critical concentration C_{ss} leads to the onset of spontaneous bulk

nucleation. Experimentally, we directly observe a rapid emergence of numerous nuclei across the film at late stages (see Figure 3 and Supplementary Video S1 to S4), which interrupts further UD growth. This observation is consistent with the model assumption that UD growth ceases once the concentration reaches C_{ss}.

(3) Spatially uniform evaporation: During spin-coating and annealing, the experiments were conducted under controlled and uniform environmental conditions (e.g. atmosphere and pressure), ensuring consistent solvent evaporation across the film. In addition, no significant spatial variation in morphology is observed prior to nucleation, supporting the approximation of spatially uniform evaporation at the scale relevant for the UD growth.

Overall, the consistency between the model prediction and experimental observation across different materials further supports the validity of these assumptions.

Page 6 (SI): "The model is established under three simplifying assumptions, which are supported by both the experimental design and direct observations as: (1) Infinite substrate assumption: The dimension of the substrate is orders of magnitude larger than the characteristic size of the unidirectional domains (typically a few hundred micrometers). Therefore, the substrate can be reasonably treated as effectively infinite for the purpose of modelling the UD growth. (2) Termination of UD growth at C_{ss}: This assumption is based on the classical nucleation theory, in which reaching the critical concentration C_{ss} leads to the onset of spontaneous bulk nucleation. Experimentally, we directly observe a rapid emergence of numerous nuclei across the film at late stages (see Figure 3 and Supplementary Video S1 to S4), which interrupts further UD growth. This observation is consistent with the model assumption that UD growth ceases once the concentration reaches C_{ss}. (3) Spatially uniform evaporation: During spin-coating and annealing, the experiments were conducted under controlled and uniform environmental conditions (e.g. atmosphere and pressure), ensuring consistent solvent evaporation across the film. In addition, no significant spatial variation in morphology is observed prior to nucleation, supporting the approximation of spatially uniform evaporation at the scale relevant for the UD growth."

Comment: (R1.5) Material defects can significantly affect the optoelectronic performance of the devices. It is recommended to provide additional characterization of defect states for materials prepared via different growth modes.

Answer: To assess defect-related carrier dynamics, we performed transient absorption (TA) measurements on $(PEA)_2SnI_4$ films with LD, UD, and SD. The normalized decay kinetics show comparable carrier relaxation dynamics among the three morphologies, indicating that the intrinsic defect-assisted recombination in the crystalline domains is not dramatically different. This result suggests that the improved FET performance of UD films is not primarily due to a large change in intrinsic bulk defect density, but rather arises from the reduced grain boundary density and improved channel morphology.

We have added the TA results as Figure S22 to the Supporting Information and described the new findings in the revised manuscript.

Page 21: "Furthermore, transient absorption measurements in **Figure S22** show comparable carrier relaxation dynamics among LD, UD, and SD films, suggesting that the carrier recombination dynamics within the crystalline domains are broadly similar across the three morphologies."

Page 19 (SI): "**Transient absorption spectroscopy** Femtosecond transient absorption (fs-TA) spectra in the visible range were obtained using a Helios pump–probe setup (Ultrafast Systems) driven by an amplified 1030 nm laser system (Pharos, Light Conversion, 200 fs, 200 μJ, 1 kHz). The fundamental laser output was converted to 400 nm using an optical parametric amplifier (Orpheus-F, Light Conversion) and employed as the excitation pump. The visible broadband probe light was generated by directing a portion of the fundamental laser through an optical delay stage and focusing it onto a sapphire crystal. The 400 nm pump and broadband probe beams were spatially overlapped and focused onto the perovskite film samples. The transmitted probe light was collected and analyzed as a function of wavelength and pump–probe delay time."

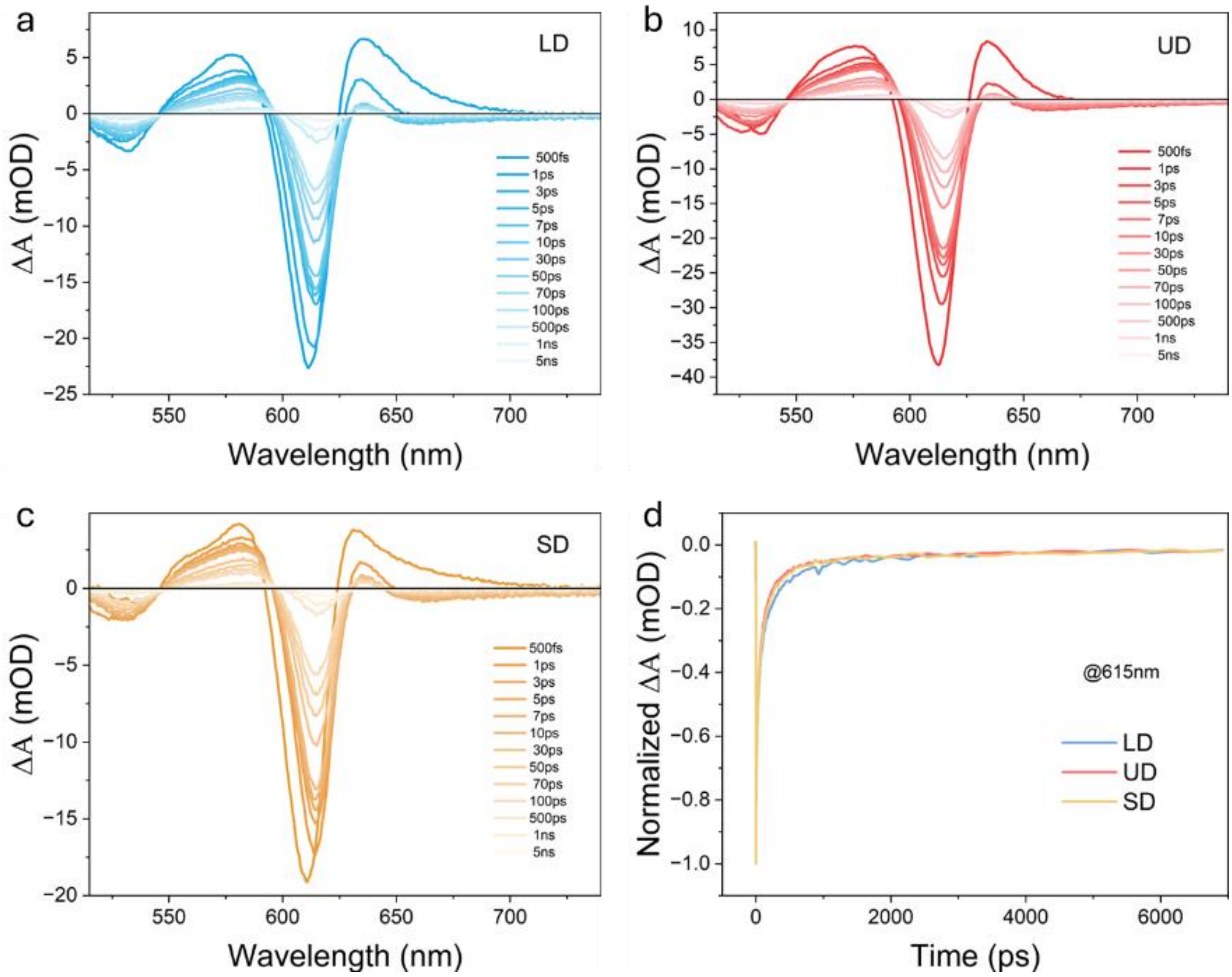

Figure S22. Transient absorption spectroscopic characterization for (a) LD, (b) UD and (c) SD of PEA_2SnI_4 films. (d) Normalized transient absorption decay kinetics monitored at 615 nm for three films.

Comment: (R1.6) The details of the FET devices are insufficient. How are SD, LD, and UD films implemented in the device architecture? How is the stability of the devices prepared via different growth modes?

Answer: We have revised the manuscript to provide a clearer description of how LD, UD, and SD films are incorporated into the FET architecture. Briefly, the semiconductor films are first prepared with the desired crystallization morphology by controlling the nucleation timing during processing, followed by thermal evaporation of the source and drain electrodes. For all the films, the channel is positioned adjacent to the scratch line to preserve film continuity while incorporating the domains into the active channel region. The scratch is located out of the channel and does not disturb the characterization of device. The FETs of SD and LD show same performance for the device with and without the scratch line. Optical microscopy images of the device geometry and channel regions have been added to the Supporting Information as Figure S20.

Regarding device stability, we have performed bias-stability measurements for FETs based on SD, LD, and UD films. The results are now included in the Supporting Information as Figure S24. These measurements show the evolution of the transfer characteristics under bias-stress at $V_{ds} = V_g = -10$ V, and provide a direct comparison of operational stability among the three growth modes. The bias-stability measurements show that UD-based devices retain the highest drain current after 1000 s of continuous bias stress, followed by LD and SD devices. The current retention after 1000 s is approximately 40% for UD, 36% for LD, and 29% for SD, indicating that the UD morphology provides the highest operational stability among the three growth modes.

We note that air-stability measurements were not performed because the devices are based on air-sensitive perovskite semiconductors and cannot be reliably measured under ambient conditions without additional encapsulation or environmental degradation. Therefore, we focus on bias stability measured under controlled conditions, which is more directly relevant for comparing the intrinsic operational stability of devices prepared with different growth modes.

We have clarified these details in the revised manuscript and Supporting Information.

Page 21: "The device geometry with the three film morphologies is shown in **Figure S20**."

Page 22: "Bias-stability measurements in **Figure S24** further reveal that UD devices exhibit the slowest current degradation under continuous gate and drain bias stress, followed by LD and SD devices, indicating improved operational stability associated with the reduced domain boundary density in UD films."

Page 32 (SI): "The channel is positioned adjacent to the scratch line to preserve film continuity while incorporating the domains into the active channel region. The scratch is located out of the channel and does not disturb the characterization of device."

Page 35 (SI): "The bias-stability measurements show that UD-based devices retain the highest drain current after 1000 s of continuous bias stress, followed by LD and SD devices. The current retention after 1000 s is approximately 40% for UD, 36% for LD, and 29% for SD,

indicating that the UD morphology provides the highest operational stability among the three growth modes."

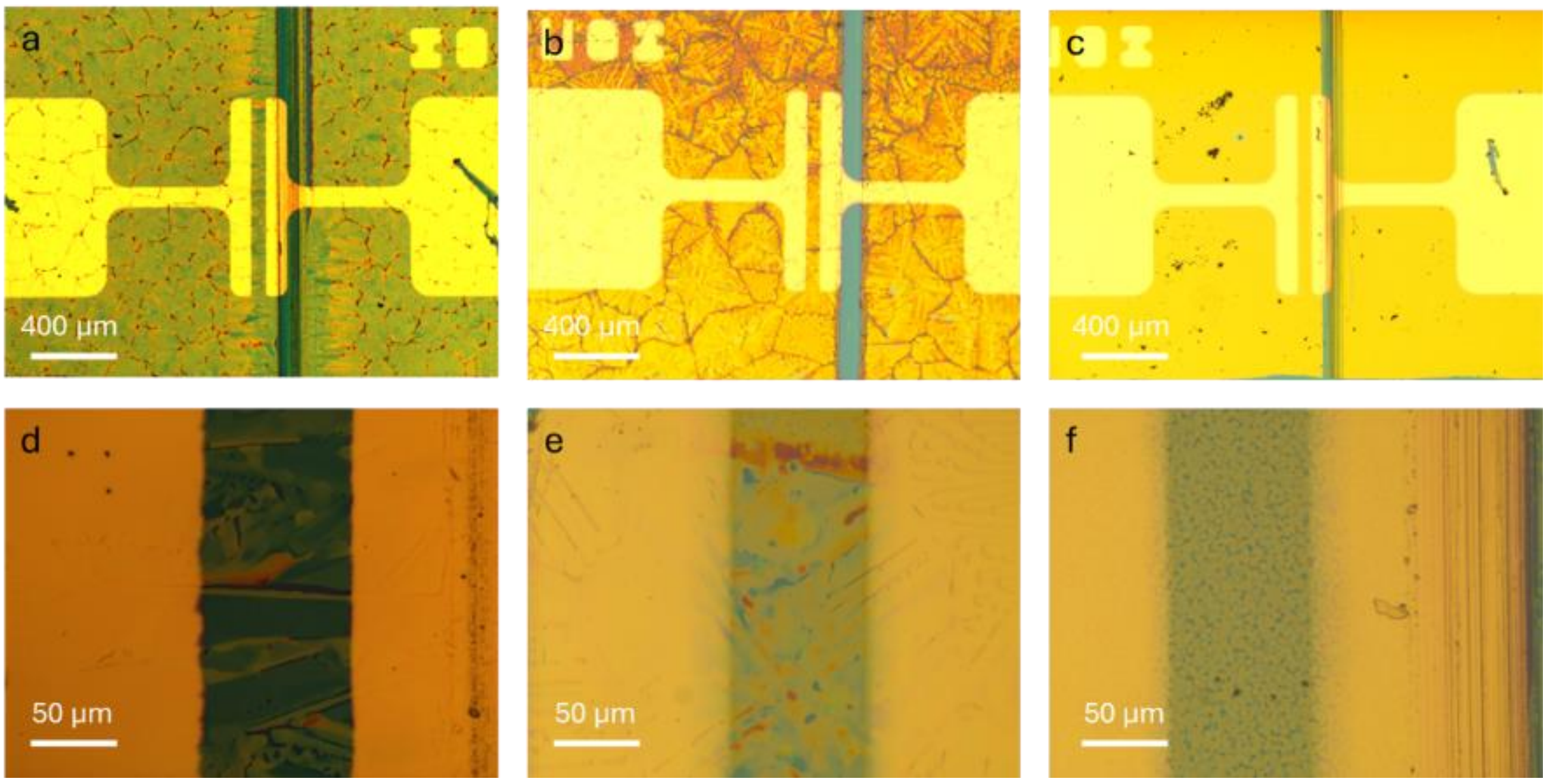

Figure S20. Device geometry and scratch configuration with (a,d) UD, (b,e) LD and (c,f) SD $(PEA)_2SnI_4$ film morphologies. The channel is positioned adjacent to the scratch to preserve film continuity.

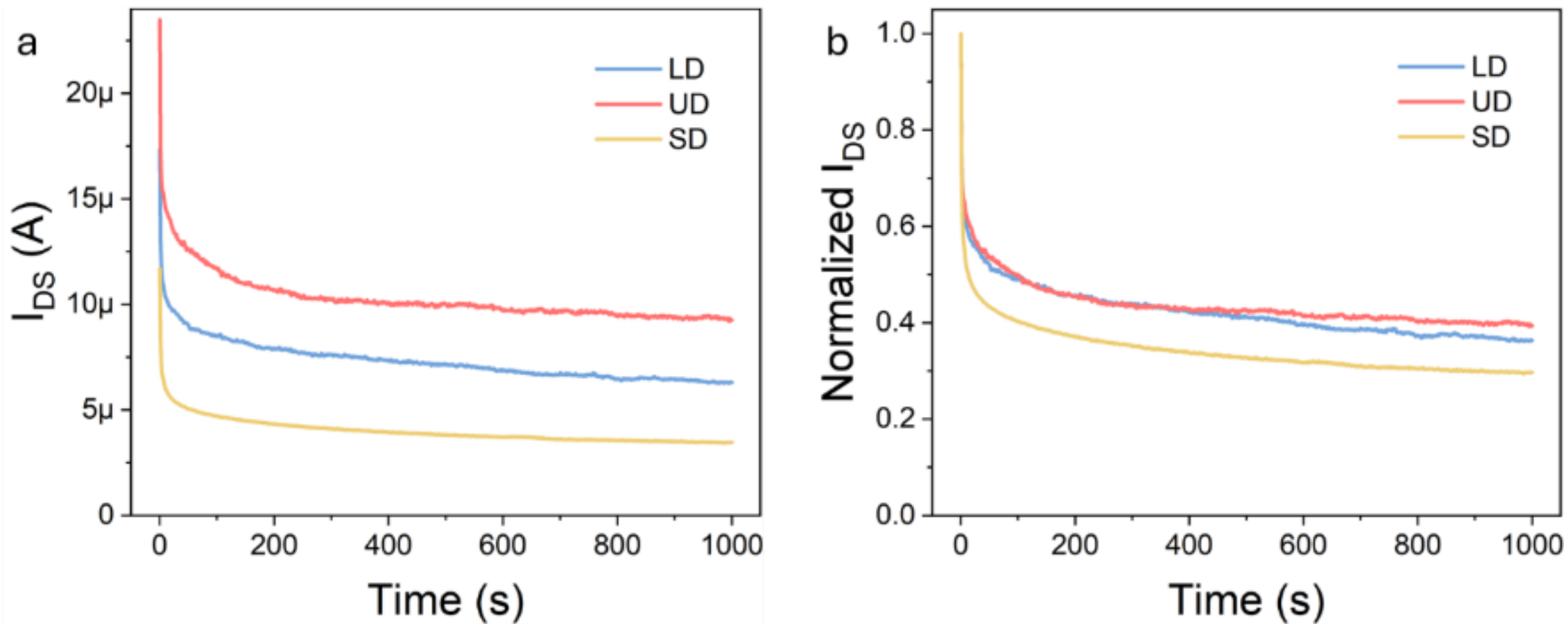

Figure S24. Bias stability of FET devices based on LD, UD, and SD $(PEA)_2SnI_4$ morphologies. (a) Evolution of $|I_{ds}|$ under continuous bias stress at $V_{ds} = V_g = -10$ V. (b) Normalized $|I_{ds}|$ as a function of stress time.

Comment: (R1.7) The device performance should be presented in a statistical manner to demonstrate the reliability of the conclusions.

Answer: To evaluate the reliability and reproducibility of the device performance, we have performed statistical measurements on multiple FET devices based on SD, LD, and UD films.

The statistical results are now included in the revised Support Information (Table S2 to S4), summarizing key device parameters including mobility and on/off ratio.

The analysis confirms that UD devices consistently exhibit superior performance compared to LD and SD devices, in agreement with the reduced grain boundary density and improved crystallinity enabled by the TDIN strategy.

Page 23: "The statistical analysis of multiple devices further confirms the improved and reproducible performance of UD-based FETs compared to LD and SD devices."

Table S2. Summary of FET performance of $(PEA)_2SnI_4$ with SD, LD and UD morphologies. Error bars are obtained from ten independently fabricated devices.

**Device**	**μ_{FET} ($cm^2V^{-1}s^{-1}$)**	**V_{th} (V)**	**$I_{on/off}$**	**SS (V dec^{-1})**	**ΔV (V)** $\lvert I_{ds}\rvert = 10^{-6}$ A
SD	0.2 ± 0.06	10.9 ± 9.8	([illegible]	6.7 ± 2.2	19.5 ± 4.3
LD	0.6 ± 0.2	13.0 ± 5.6	([illegible]	8.1 ± 1.2	12.8 ± 4.9
UD	2.6 ± 0.7	6.1 ± 4.1	([illegible]	5.0 ± 1.4	10.2 ± 4.2

Table S3. Summary of FET performance of $(MeOPEA)_{1.8}Cs_{0.1}SnI_{3.9}$ *with SD, LD and UD morphologies. Error bars are obtained from ten independently fabricated devices.*

**Device**	**μ_{FET} ($cm^2V^{-1}s^{-1}$)**	**V_{th} (V)**	**$I_{on/off}$**	**SS (V dec^{-1})**	**ΔV (V)** $\lvert I_{ds}\rvert = 10^{-6}$ A
SD	0.3 ± 0.1	4.5 ± 4.0	([illegible]	5.7 ± 1.0	15.3 ± 4.6
LD	0.8 ± 0.2	-2.8 ± 2.2	([illegible]	5.0 ± 0.8	11.4 ± 2.9
UD	2.8 ± 0.2	-2.4 ± 1.6	([illegible]	4.8 ± 0.9	5.8 ± 1.2

Table S4. Summary of FET performance of TIPS-pentacene with SD, LD and UD morphologies. Error bars are obtained from ten independently fabricated devices.

Device	μ_{FET} ($cm^2V^{-1}s^{-1}$)	V_{th} (V)	$I_{on/off}$	SS (V dec^{-1})	ΔV (V) $\lvert I_{ds}\rvert = 10^{-6}$ A
SD	0.2 ± 0.09	-9.0 ± 8.2	([illegible]	10.4 ± 0.7	6.3 ± 0.8
LD	0.5 ± 0.05	-7.5 ± 5.4	([illegible]	10.1 ± 0.5	7.1 ± 0.7
UD	0.7 ± 0.05	-3.4 ± 2.7	([illegible]	9.5 ± 0.4	5.5 ± 0.4

Comment: (R1.8) How can the horizontally grown crystallization mode be applied to devices such as solar cells that require a vertical structure?

Answer: We agree that the unidirectional (UD) growth demonstrated in this work is primarily oriented in-plane and is particularly beneficial for lateral transport devices such as field-effect transistors. In vertical devices, charge transport occurs perpendicular to the film plane, but is still strongly influenced by grain boundaries and defects distributed throughout the film. Therefore, strategies that reduce defect density and improve crystallinity, as demonstrated here, can contribute to improved device performance even without explicit in-plane alignment.

While the present study demonstrates in-plane unidirectional growth, the underlying model offers a general strategy for controlling crystallization kinetics and defect formation, which is relevant for both lateral and vertical device architectures. Furthermore, the TDIN strategy provides a general framework for controlling crystallization kinetics, which may be adapted to different processing geometries and device architectures through appropriate tuning of the processing conditions. Since grain boundaries and defect distributions influence charge transport and recombination in both lateral and vertical devices, the TDIN strategy is expected to be broadly applicable to solution-processed optoelectronic devices beyond FET architectures.

Page 23: "The developed model offers a general strategy for understanding and controlling crystallization kinetics and defect formation relevant for both lateral and vertical device architectures. Since grain boundaries and defect distribution influence charge transport and recombination in both lateral and vertical devices, this universal strategy bears a broad potential for precise tailoring and elevating the performance of solution-processed optoelectronic devices beyond FET architectures."

Reviewer: 2

Comment: This manuscript introduces a time-defined induced nucleation strategy to separate the nucleation and growth times that enables semiconductors to crystallize at distinct stages during solution processing. From this study, the authors come up with a model to capture the essential features of semiconductor nucleation and growth in solution across different processing conditions. The work is of current interest and the approach is neat with some potentially valuable results. However, the distinct aspects of the developed model are not clearly articulated, especially against the benchmark models of LaMer， Avrami, etc.--it seems to be still mainly based on classical nucleation theory, which needs to be justified on its own, leaving the induced nucleation only an experimental design innovation. Also, the wider relevance and presentation of the manuscript need to be much improved to warrant the publication. More detailed comments are given below.

Answer: We thank the reviewer for the careful assessment of our work. It is true that the present model is built upon established concepts from classical nucleation and crystallization theories. However, the key advance of this work is not the replacement of classical nucleation theory itself, but the extension of these concepts into a deterministic and experimentally accessible framework.

In contrast to conventional models such as LaMer or Avrami, which primarily describe ensemble-averaged nucleation density and bulk crystallization kinetics, the model based on the TDIN strategy resolves and permits a fine control over individual heterogeneous nucleation events and their subsequent domain growth under different processing conditions. This model enables quantitative prediction of the LD-UD-SD morphology evolution across different materials, which is not possible to achieve using conventional ensemble-based crystallization models.

In the revised manuscript, we have substantially clarified the distinction between the present framework and conventional crystallization models, and improved the discussion of its broader applicability and physical significance.

Page 4: "While classical crystallization theories describe an ensemble-averaged behavior, the present work establishes a model to resolve and control the evolution of individual domains under heterogeneous conditions."

Our point-by-point responses are provided below.

Comment: (R2.1) The demonstrated semiconductor systems are mainly low-dimensional perovskites, why not 3-dimensional perovskites that are actually the workhorse of the now most promising perovskite solar cells? This inclusion will give very forceful support to the model.

Answer: We agree that demonstrating the applicability of the model to 3D perovskites is important. In fact, a 3D perovskite is already included in this work. As shown in Figures 1g and S4, the $MAPbI_3$ 3D perovskite exhibits the same LD–UD–SD morphology evolution and follows the same crystallization behavior described by the TDIN model. Since $MAPbI_3$ is a

representative 3D perovskite widely used as semiconductor in solar cells, these results support the applicability of the model to solution-processed 3D perovskite systems.

Comment: (R2.2) There should be discussion how the model can be practically used to design processing conditions of semiconductor devices, especially with a large size.

Answer: The practical significance of the TDIN model is its ability to define the concentration-dependent nucleation windows governing different crystallization pathways. By identifying the critical concentrations (C_i and C_{ss}) governing the onset and end of induced nucleation, the model provides a quantitative guideline for controlling the time and location of the nucleation during solution processing.

This is particularly relevant for large-area semiconductor fabrication, where uncontrolled nucleation and non-uniform crystallization often lead to significant film inhomogeneity and device-to-device variation. Our work offers a strategy to regulate crystallization kinetics through processing parameters, thereby enabling more deterministic control over film morphology. The model provides guidelines for controlling the nucleation window and crystallization pathways during solution processing. Importantly, although the present work demonstrates the concept using spin-coating, the underlying model is general and can be extended to other scalable solution-processing methods involving controlled crystallization.

We have added a discussion in the revised manuscript to clarify the practical implications of the model for semiconductor processing and large-area device fabrication.

Page 14: "More importantly, the ability to define the concentration-dependent nucleation window provides a practical model for controlling the crystallization pathways and improving film uniformity in solution-processed semiconductor films, particularly for large-area processing."

Comment: (R2.3) Different nucleation mechanisms are involved, both induced and spontaneous. Would it make analysis more difficult since they interfere with each other and we need distinguish between the two? Is it possible to only allow induced one since the spontaneous one has higher energy cost?

Answer: As described in the manuscript, the key principle of the TDIN model is the temporal and spatial differentiation between induced nucleation and spontaneous nucleation pathways.

For spontaneous bulk nucleation leading to SD formation, the separation is primarily temporal. Within the experimentally accessible concentration window ($C_i \leqslant C < C_{ss}$), induced nucleation occurs first at the cusp region, while spontaneous bulk nucleation remains suppressed until the concentration reaches C_{ss}. Therefore, the two processes are temporally separated and do not directly interfere during the initial stage of UD formation.

For spontaneous interfacial nucleation associated with LD formation, partial temporal overlap may occur at later stages. However, these spontaneous nuclei are spatially separated from the

induced nucleation and therefore do not prevent the initial establishment of UD growth. Instead, the subsequent growth of nearby LD regions may gradually limit further lateral expansion of UD domains, which is consistent with both the model and experimental observations.

Importantly, the model does not rely on eliminating spontaneous nucleation, but rather on controlling the temporal sequence and spatial separation between different nucleation pathways.

We have further clarified this point in the revised manuscript.

Page 9: "Importantly, the TDIN model does not exclude spontaneous nucleation, but instead exploits the temporal and spatial separation between induced and spontaneous nucleation pathways to control the crystallization behavior."

Comment: (R2.4) The films from the strategy are quite inhomogeneous globally, making it unsuitable for devices. Even for characterizations such as GIWAX, mobility, etc., they are referred to specific parts of the films?

Answer: In the present work, the spatially differentiated morphology is intentionally introduced to resolve and to analyse distinct crystallization pathways within a single film. Accordingly, all characterizations were performed on specifically selected regions of the films. For GIWAXS measurements, the X-ray beam was aligned parallel to the scratch line to preferentially probe the UD regions, as clarified in the revised manuscript. Similarly, the FET channels were positioned adjacent to the scratch line to ensure that the measured transport properties reflect the representative LD, UD or SD morphology.

Importantly, the purpose of the present work is not to fabricate globally homogeneous device films, but to establish a model for understanding and controlling nucleation and growth pathways in solution-processed semiconductors. The insights obtained from these spatially differentiated regions provide mechanistic guidance for future crystallization engineering and morphology control.

We have clarified these points in the revised manuscript.

Page 20: "To investigate the influence of distinct crystallization pathways on the resulting structural and electronic properties, representative LD, UD, and SD regions of the films were selectively characterized."

Page 21: "The spatially differentiated morphologies enable a direct comparison between distinct crystallization pathways and their corresponding electronic properties. The device geometry with the three film morphologies is shown in **Figure S20**."

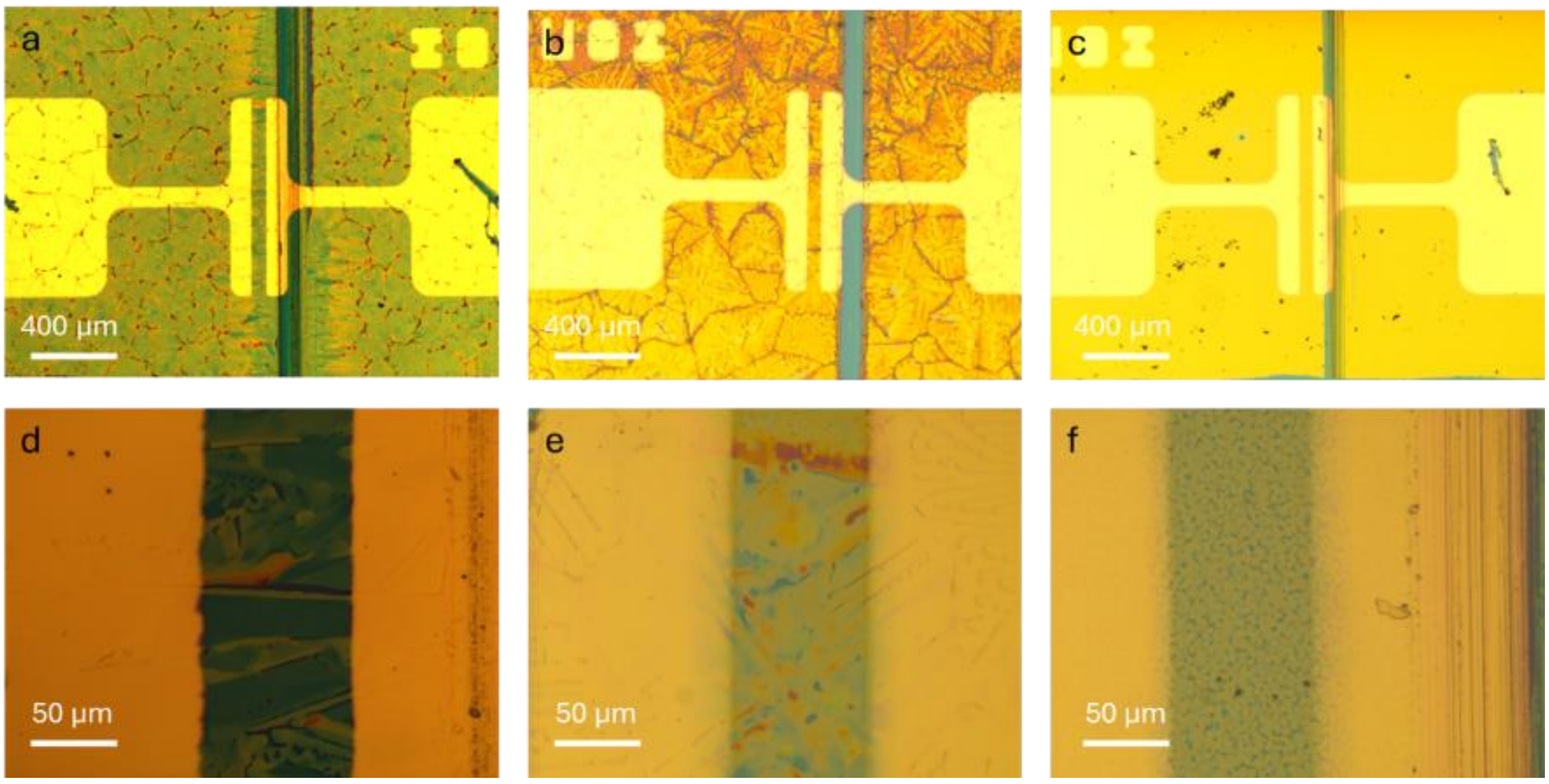

Figure S20. Device geometry and scratch configuration with (a,d) UD, (b,e) LD and (c,f) SD $(PEA)_2SnI_4$ film morphologies. The channel is positioned adjacent to the scratch to preserve film continuity.

Page 16 (SI): "For the film with unidirectional domains, the X-ray beam was positioned to overlap with the UD region, ensuring that the scattering signal is dominated by the aligned domains."

Comment: (R2.5) Length L's are variously used, but they do not seem to be defined clearly. While we may see L is represented by areas in concentration vs time plots, they are not clearly illustrated in the actual film images and how we get the L values from the images. By the way, in Fig M1(SI), L is also shown, is that the same L we are talking about?

Answer: In the present work, L consistently refers to the real-time lateral growth length of the crystal during crystallization, including the one used in Figure M1 (SI). However, L^{subs} in Figure M1 means the length of the substrate.

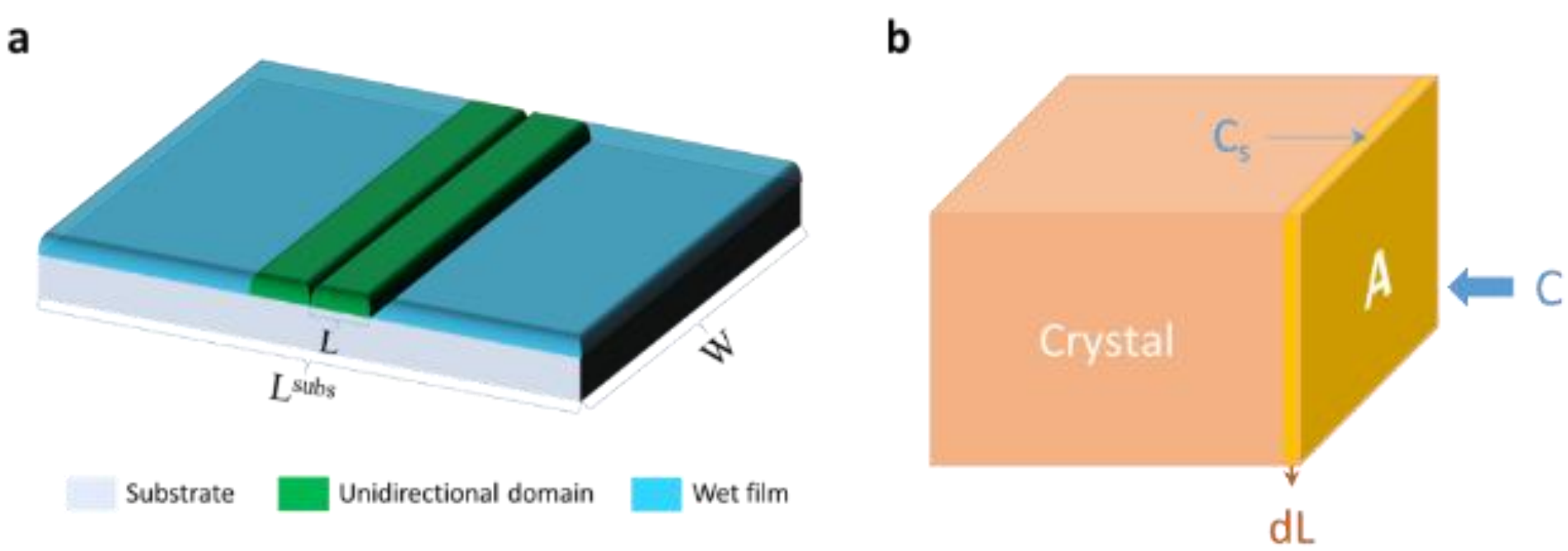

Figure M1. The schematic diagram of the geometry important for the (a) solute diffusion and (b) mass transfer theory.

We also have another important value L^{final} established in the model, which means the final UD length after crystallization. To explicitly illustrate how L and L^{final} are experimentally defined and extracted, we have added Figure S13. Figure S13a illustrates L^{final}, which is extracted from optical microscopy images after the crystallization is completed. In contrast, Figure S13b illustrates L, which is extracted from time-resolved video frames recorded during UD growth. During annealing, L evolves continuously with crystallization time and eventually reaches L^{final} after crystallization is completed.

We have added this explanation in the revised manuscript.

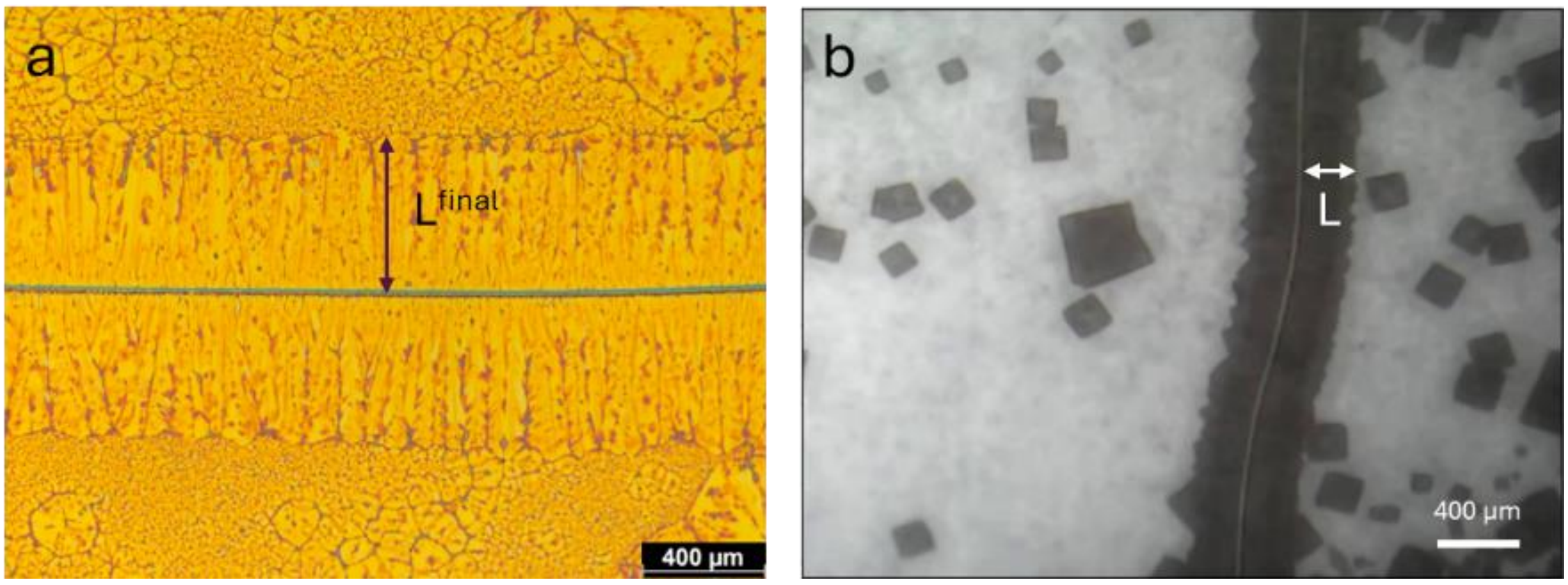

Figure S13. Definition and experimental extraction of the characteristic lengths (a) L^{final} extracted from optical microscopy image and (b) L extracted from time-resolved video frames during annealing.

Page 16: "As shown in **Figure S13**, L^{final} is determined from optical microscopy images after crystallization is completed, while L is extracted from time-resolved video frames during crystallization."

Comment: (R2.6) There are also many other parameters such as Css, many different t's, etc., which are not clearly defined as well. For example, what is meant by supersaturation for Css? quite confusing.

Answer: The characteristic concentrations and times used for the model are summarized in the "List of Abbreviation for the Model" in the Supporting Information. As discussed in the description of the three crystallization regimes on page 9 of the manuscript, C_i and C_{ss} define the boundaries of the concentration window governing the onset and end of induced nucleation, respectively. In particular, C_i denotes the critical concentration for mechanically induced nucleation, while C_{ss} corresponds to the critical concentration for spontaneous bulk nucleation.

To clarify the definitions of the various time parameters used in the model, we have revised their definitions and explicitly linked them to the corresponding concentrations. As shown in Figure 2b, t^s denotes the spin-coating time, t_i^s corresponds to the spin-coating time when $C=C_i$,

and t_0^s is the spin-coating time when C=C0. While t^a is the annealing time, t_0^a is the annealing time when C=C0, and t_{ss}^a marks the annealing time when C=Css.

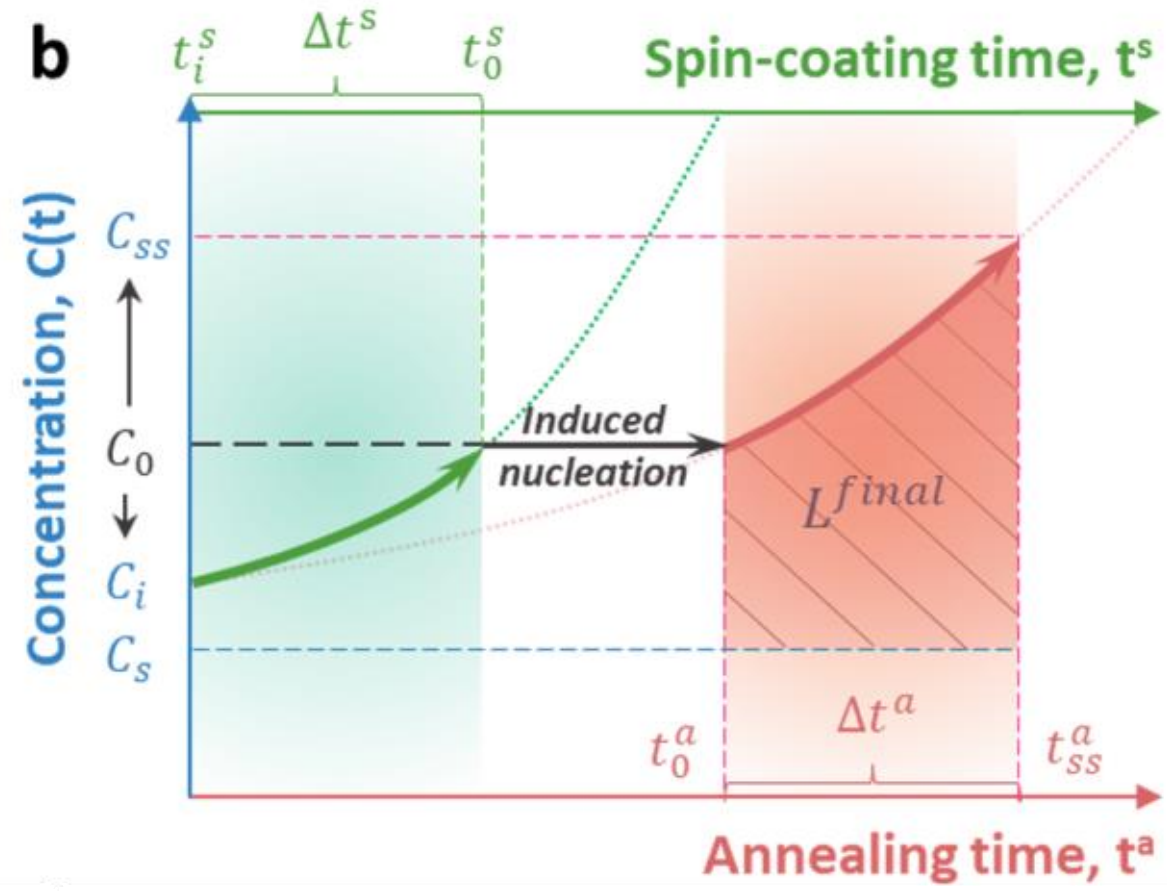

Figure 2. (b) Modelling the relation between the concentration of the wet film to spin-coating and annealing times.

To further avoid ambiguity, we have revised the definitions of Ci, Css, and the characteristic time parameters for improved consistency and readability.

The part change of the 'List of Abbreviation for the Model' in Supporting Information:

C_i	mol/L	Critical concentration for mechanically induced nucleation
C_{ss}	mol/L	Critical concentration for spontaneous bulk nucleation
t_0^s	s	Spin-coating time when C=C0
t_i^s	s	Spin-coating time when C=Ci
t_{ss}^s	s	Spin-coating time when C=Css
t_0^a	s	Annealing time when C=C0
t_i^a	s	Annealing time when C=Ci
t_{ss}^a	s	Annealing time when C=Css

Page 8: "Ci (critical concentration for mechanically induced nucleation)", "Css (critical concentration for spontaneous bulk nucleation)."

Page 13: "t_i^s corresponds to the spin-coating time when C=Ci, t_0^s is the spin-coating time when C=C0, t_0^a is the annealing time when C=C0, and t_{ss}^a marks the annealing time when C=Css."

Comment: (R2.7) Many equations have problems, for example, Eq S9, why plus sign in the denominator? How can one go from Eq S11 to S12? It is said that one can get max t if t's are zero in Eq S12, but if then, the left side becomes all zero.

Answer: We thank the reviewer for checking the derivation carefully. The different signs in Eqs. S8 and S9 arise from the different reference states used for the two processing stages. Eq. S8 describes the forward concentration increase during spin-coating from C_i to C_0. In this case, solvent evaporation reduces the wet-film volume, leading to a negative term in the denominator. In contrast, Eq. S9 is written by taking C_{ss} as the reference final concentration at the end of the annealing growth window and back-calculating the wet-film volume corresponding to C_0. Therefore, the solvent volume evaporated during annealing is added back to the final wet-film volume at C_{ss}, giving rise to the positive term in the denominator.

To further clarify the transition from Eq. S11 to Eq. S12, we have added the reciprocal form of Eq. S11 in the revised Supporting Information. Equating the two expressions for $1/C_0$ directly leads to Eq. S12. We have also revised the wording regarding Δt^s_{max} and Δt^a_{max} to clarify that they are obtained from two separate limiting boundary conditions, $\Delta t^a = 0$ and $\Delta t^s = 0$, respectively, rather than by setting both time intervals to zero simultaneously.

We have revised the supporting information as following:

Page 7 (SI): "It should be noted that Equation S8 is written in the forward direction from C_i to C_0 for the spin-coating process, whereas Equation S9 is written in the reverse direction from C_{ss} back to C_0 during annealing. Therefore, the evaporated solvent volume is subtracted in Equation S8 but added back in Equation S9."

Page 7 (SI): "It should be noted that $\Delta t^s = t^s_0 - t^s_i$ and $\Delta t^a = t^a_{ss} - t^a_0$. Taking the reciprocal form of Equation S11 gives"

Page 7 (SI): "$\frac{1}{C_i} - \frac{Q_{spin}}{\alpha}\Delta t^s = \frac{1}{C_0} = \frac{1}{C_{ss}} + \frac{Q_{anneal}}{\alpha}\Delta t^a$"

Page 7 (SI): "Δt^s_{max} and Δt^a_{max} are obtained from two separate limiting boundary conditions, namely $\Delta t^a = 0$ and $\Delta t^s = 0$, respectively"

Comment: (R2.8) SI, Fig M2: only 3 points, enough to make a case? Also why only for UD? Actually, I wonder why do we have SD, UD, LD instead of SD, MD (medium domain), LD? It seems UD need to be explained more clearly.

Answer: Figure M2 is intended to illustrate the experimentally observed trend and corresponding agreement with the model. Although only a limited number of experimental points are shown, the measured results consistently follow the predicted growth trend under different processing conditions, supporting the mechanistic validity of the model.

Regarding the question of why the quantitative derivation is presented using UD growth, we note that UD provides a well-defined and experimentally trackable growth front, allowing the real-time growth length L to be directly extracted during crystallization. This makes the UD

morphology a suitable model system for establishing the quantitative growth model. But the model is not restricted to UD. As shown in Figure 3d and 3g, by comparing the growth of UD and LD, we demonstrate that the diffusion-based UD growth model can also describe LD growth, confirming that the model captures a general growth mechanism rather than a UD-specific case.

Importantly, UD does not denote a "medium domain" morphology. In the present work, SD, LD, and UD represent distinct crystallization pathways and morphology evolution modes rather than a simple classification based solely on domain size. Specifically, UD refers to the unidirectional growth morphology induced by the mechanically generated nucleation front, characterized by anisotropic domain growth perpendicular to the scratch during crystallization.

We have clarified the definition of UD in the revised manuscript.

Page 6: "*...characterized by the anisotropic growth perpendicular to the scratch...*"

Page 13 (SI): "Figure M2 illustrates the experimentally observed trend and corresponding agreement with the model. Although only a limited number of experimental points are shown, the measured results consistently follow the predicted growth trend under different processing conditions, supporting the mechanistic validity of the model."

Comment: (R2.9) Nucleation is induced at a specific location, so diffusion should play a very important role. But the model seems to have no information on diffusion parameters.

Answer: We agree that diffusion and solute transport play important roles in the localized growth of UD domains after induced nucleation. In the present model, these effects are already incorporated through the mass transfer coefficient k_c, which is introduced to describe the solute transport kinetics during crystallization. The resulting UD growth reflects the combined effects of diffusion, solvent evaporation, and concentration evolution during spin-coating and annealing. Importantly, the good agreement between the model and experimental results across different materials and processing conditions indicates that the model well captures the kinetic processes of the morphology evolution.

Page 10 (SI): "Here, the mass transfer coefficient k_c reflects the combined effects of diffusion, solvent evaporation, and concentration evolution during the crystallization process."

Comment: (R2.10) SI Eq 52: There are also problems here, such as photoconductivity change (delta), film (not flim).

Answer: We thank the reviewer for pointing out these errors in Eq. 52 and the corresponding discussion in the Supporting Information. The notation for the photoconductivity change ($\Delta\sigma$), as well as typographical errors such as "flim" have been corrected in the revised version. We have also carefully revised the equation formatting and symbol definitions throughout the Supporting Information to improve clarity and consistency.

$$\frac{\Delta\sigma}{N_{abs,film}} = \left(-\frac{\varepsilon_0 c\,(n_1+n_2)}{d}\cdot\frac{\Delta E}{E}\right)\cdot\frac{d}{N_{abs}} = \varphi\mu \qquad \text{(Eq. S52)}$$

Comment: (R2.11) Line 427: mobility inccrease with morphology? (change with morphology?) Why UD morphology is the best?

Answer: The wording has been corrected from "The μ_{FET} increases systematically with the film morphology for both cases." to "Both semiconductors show an increase in μ_{FET} for films with larger domain size." at page 22 of the revised manuscript.

The superior device performance of the UD morphology is mainly attributed to its anisotropic domain growth and continuous transport pathways. Compared with SD and LD films with randomly distributed domain boundaries, the UD morphology provides more continuous charge-transport channels with reduced domain-boundary interruption along the transport direction. This interpretation is also consistent with the GIWAXS, KPFM, and THz measurements, which together indicate improved crystallographic ordering and directional carrier transport for the UD morphology.

We have further clarified the morphology-dependent transport mechanism of UD in the revised manuscript.

Page 22: "The enhanced device performance of the UD morphology is attributed to its anisotropic domain growth and continuous charge-transport pathways, which reduce domain-boundary as trapping sites across the device channel."

Comment: (R2.12) Fig 3abcd: which is LD, UD, LD? They should be more clearly labeled.

Answer: To more clearly distinguish the LD, UD, and SD morphologies in Fig. 3a–d, we have added Figure S17 in the Supporting Information showing the last video frames with explicit morphology labels.

Page 16: "The corresponding LD, UD, and SD regions in **Figure 3a-d** are more clearly labeled in **Figure S17**."

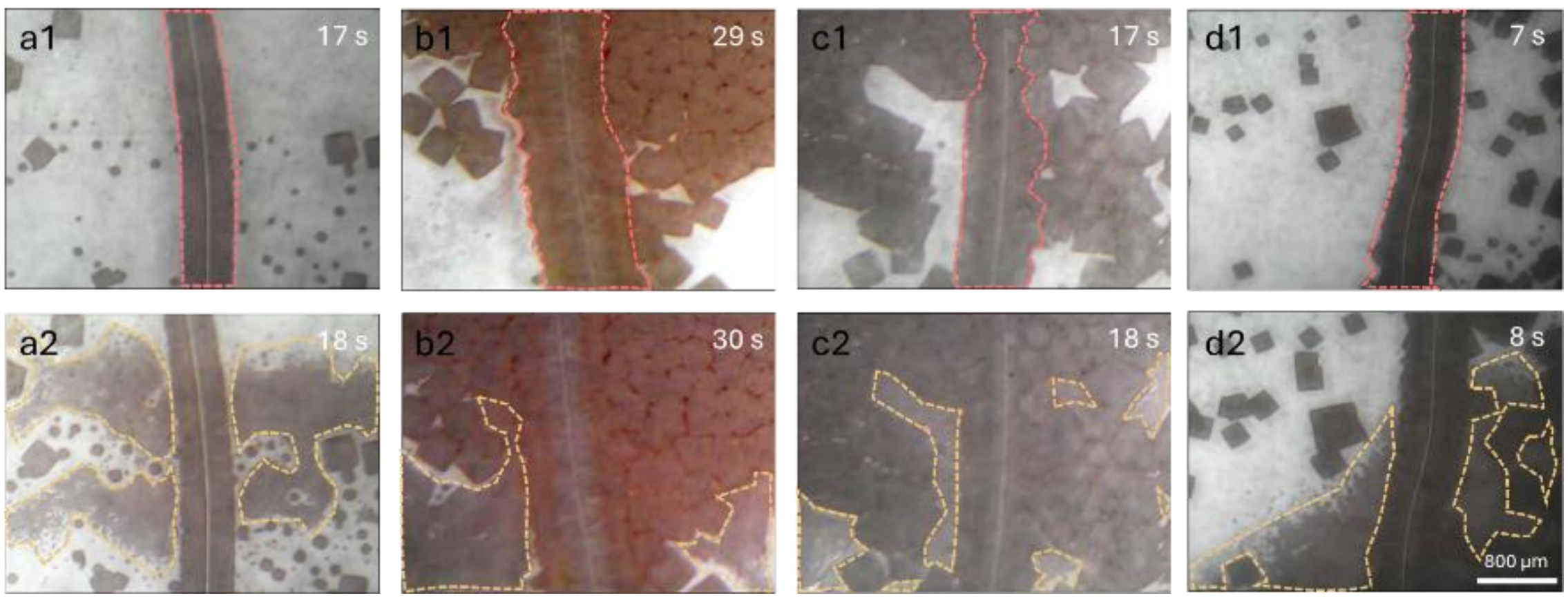

Figure S17. Labeled crystallization video frames corresponding to Figure 3a-d, with an identification of the UD, LD, and SD morphologies. Panels a1-d1 show the UD regions highlighted by red dashed outlines. The dark areas outside the red regions correspond to LD morphologies formed through spontaneous nucleation at earlier annealing times. Panels a2-d2 show the subsequent video frames recorded 1 s later, where the newly emerged dense nucleation regions highlighted by yellow dashed outlines correspond to the SD morphology.

Comment: (R2.13) Check the whole manuscript and SI, e.g., SI, line 71: time interval, ...

Answer: We have carefully rechecked the entire manuscript and Supporting Information to improve the consistency of the terminology throughout the text. The identified issues have been corrected in the revised version.

Reviewer: 3

Comment: The work by Ling et al. reported an innovative method to decouple the nucleation and crystal growth process in a wide range of solution-processed semiconductor thin films. Time-defined induced nucleation (TDIN) is achieved by scratching the wet film within a specific time window after spin coating, during which the supersaturation is high enough for scratching to induce heterogeneous nucleation yet low enough to avoid homogenous nucleation in the bulk. The subsequent thermal annealing then allows the author to draw a direct correlation between crystal size and annealing time, which can be described by a diffusion-based model. I think this work provides theoretical insights for optimising the fabrication procedure of solution-processed optoelectronic thin films, especially for metal-halide perovskites, where the time interval between spin coating of wet film and the onset of thermal annealing is known to critically determine the performance of perovskite PVs. Induced crystal growth perpendicular to the scratch line is also beneficial for solution-processed FETs, where crystal alignment along the channel length direction can help reduce charge trapping at grain boundaries, as demonstrated by the authors. Therefore, I recommend that this paper be published in Nature Communications after addressing the following concerns

Answer: We thank the reviewer for the positive and encouraging assessment of our work, and for recognizing both the fundamental significance of the proposed TDIN strategy and its relevance for practical applications in solution-processed optoelectronic devices. In response to the reviewer's comments, we have further clarified several aspects of the manuscript and provided additional discussion to address the concerns raised. Our point-by-point responses are provided below.

Comment: (R3.1) In Figures 1f and g, the author demonstrates the TDIN strategy on five different material systems. While the universal LD-UD-SD evolution is clearly seen in 2D perovskite, 2D/2D perovskite, and 3D perovskite, the trend becomes less obvious in TIPS-pentacene and inorganic salts. In the latter two cases, clear large domains can still be identified in the SD figures, which requires further clarification.

Answer: We agree that the LD-UD-SD evolution is most visually distinct in the perovskite systems, whereas the distinction is less pronounced for TIPS-pentacene and especially for the inorganic salt system.

For TIPS-pentacene, additional SEM measurements (Figure S5) reveal that the apparent large features in the optical images of the SD are composed of densely packed smaller domains less than 1 µm, whereas the LD morphology consist of domains larger than 100 µm. This confirms that the LD-UD-SD evolution is still valid for TIPS-pentacene.

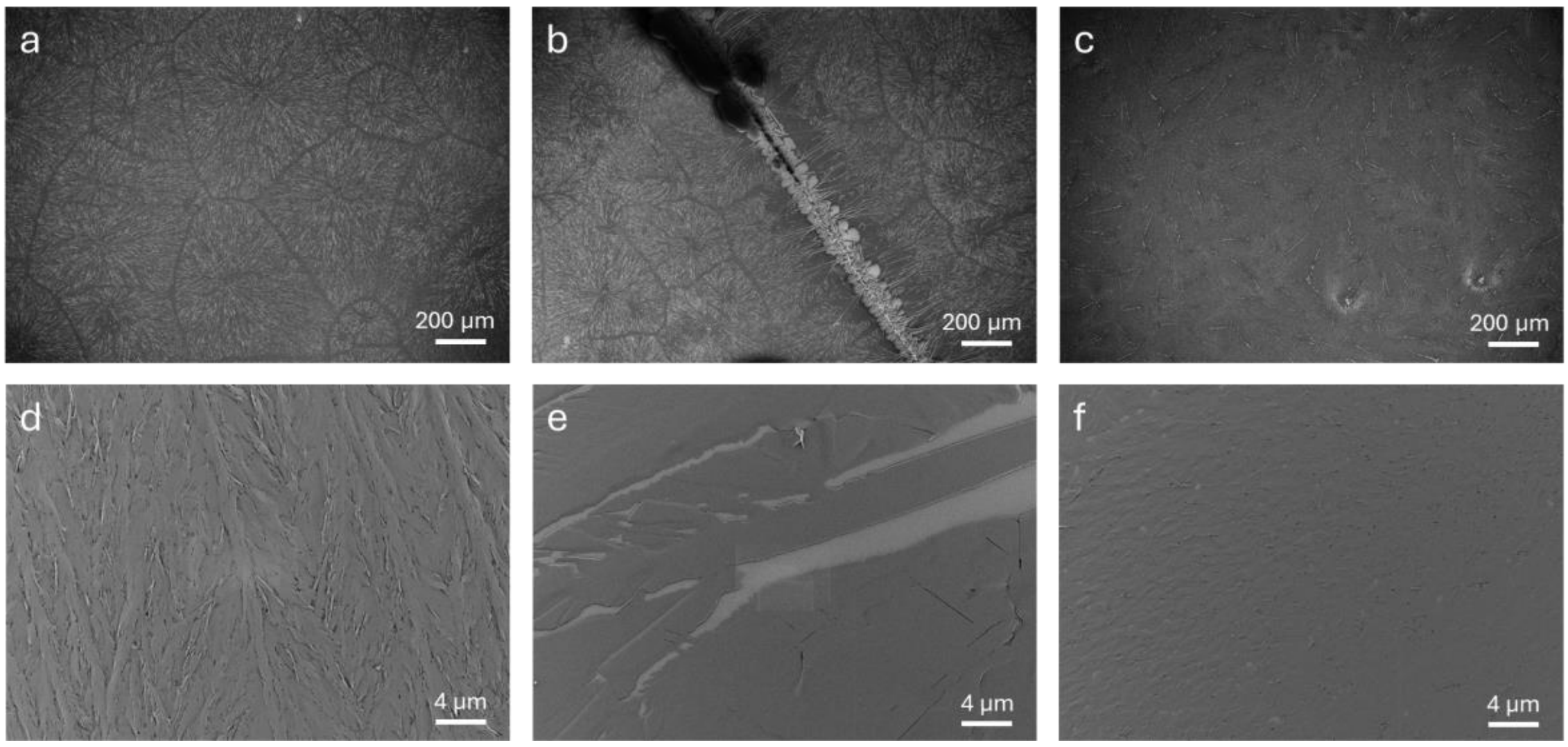

Figure S5. Scanning electron microscopy images of TIPS-pentacene films with (a,d) LD, (b,e) UD and (c,f) SD.

For the inorganic salt system, although the distinction between LD and SD appears less obvious in the optical images, additional SEM measurements (Figure S6) reveal that the LD morphology consists of large crystals exceeding 200 µm, whereas the SD morphology is composed of densely packed smaller domains below 20 µm. This observation is consistent with the behavior observed for TIPS-pentacene and further confirms that the LD-UD-SD evolution remains valid across different material systems.

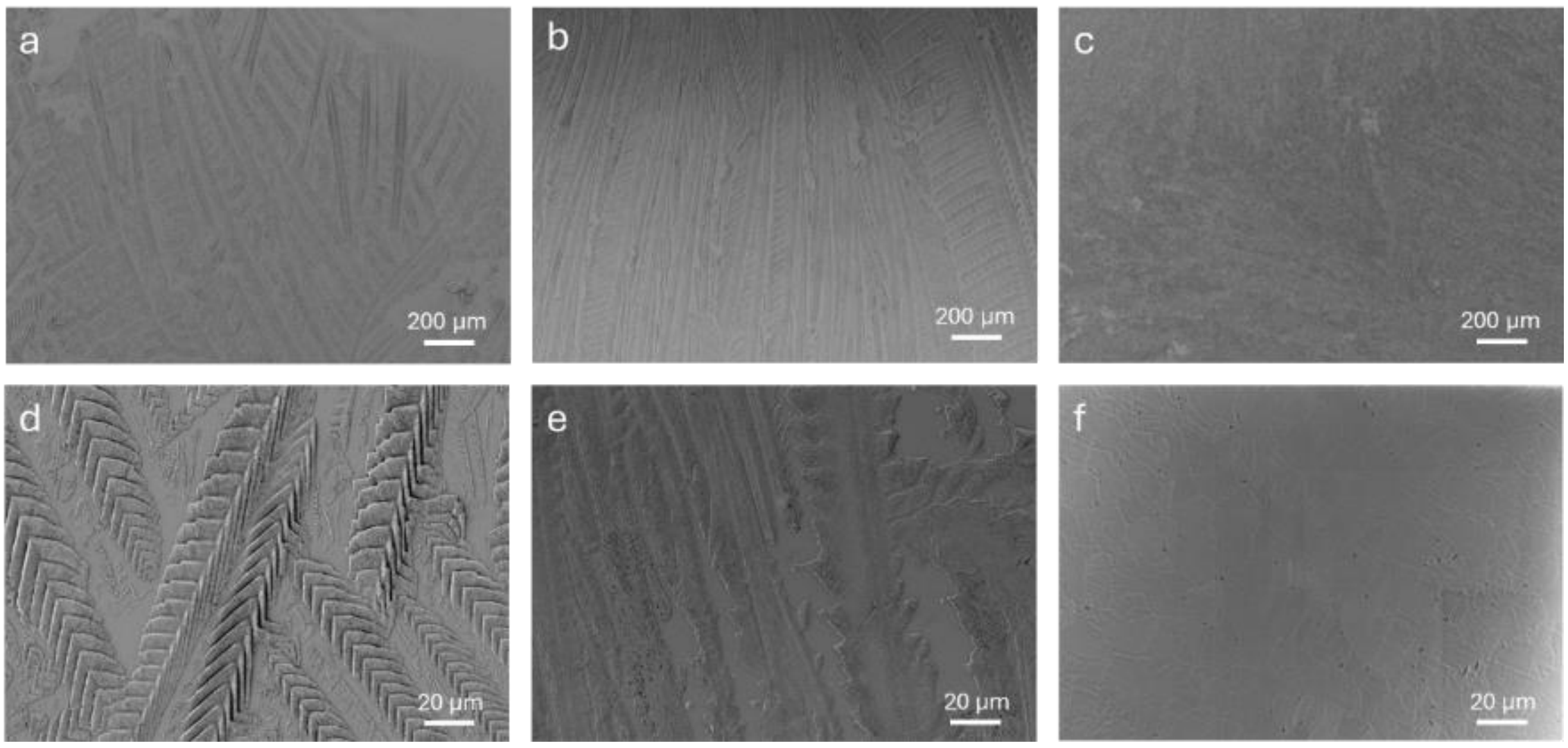

Figure S6. Scanning electron microscopy images of NaH_2PO_4 films with (a,d) LD, (b,e) UD and (c,f) SD.

This clarification has been added to the revised manuscript, together with additional SEM data for supplementary microscopic characterization (Figure S2 to S6).

Page 10: "Scanning electron microscopy images further confirm that the distinct LD, UD, and SD morphologies are preserved at the microscale, in agreement with the macroscopic optical images (**Figure S2** to **S6**). We note that the absolute domain sizes associated with the LD, UD, and SD regimes can vary between materials while still preserving the characteristic morphology evolution."

Comment: (R3.2) In this paper, the author considered heterogeneous nucleation initiated at the tip, the solution-air interface, and homogenous nucleation within the solution bulk. In practice, heterogeneous nucleation can also initiate at the solution-substrate interface, which should be taken into account, given that a diverse range of substrates with different surface energies has been used.

Answer: We agree that heterogeneous nucleation may also occur at the solution/substrate interface, depending on the relative surface energies of the system. In our experiments, different substrates (e.g. silicon dioxide, glass, and quartz) were employed, and consistent LD-UD-SD morphological evolution was observed across all cases.

We note that the dominant nucleation pathway may vary between the air/liquid interface and the substrate/liquid interface depending on material-specific interfacial energetics. However, the nucleation condition in all cases is governed by the same fundamental criterion based on the relation between cluster size and critical radius. Furthermore, the mechanically induced nucleation at the cusp region provides the lowest nucleation barrier and dominates the induced nucleation process for UD. Therefore, the overall crystallization pathway and the validity of the model remain unchanged.

We have clarified this point in the revised manuscript.

Figure 1b:

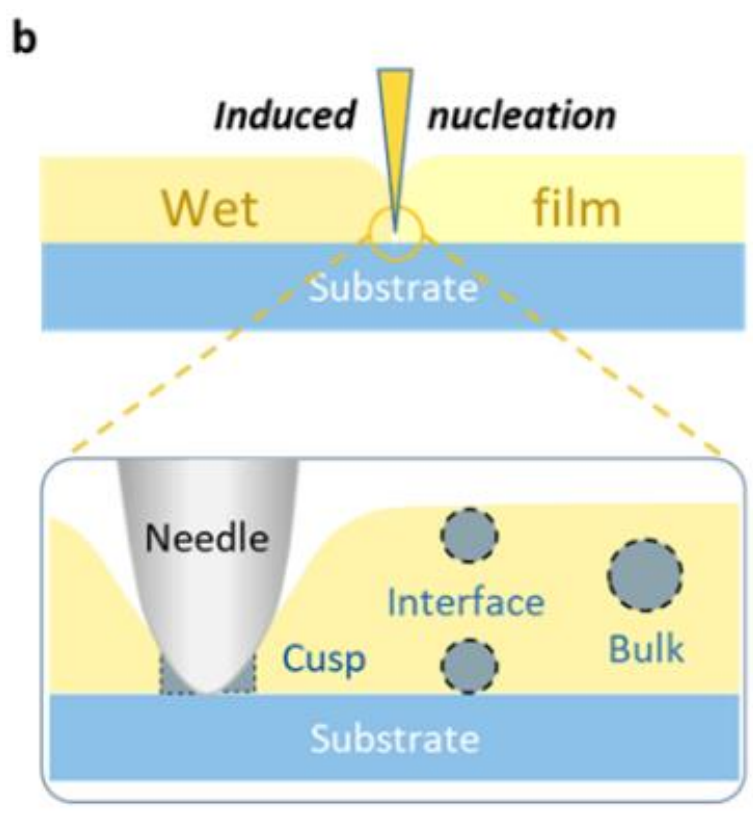

Page 6: "... or liquid/substrate ..."

Page 7: “The nucleation sites can be located in the bulk (homogeneous nucleation) the liquid/air or liquid/substrate interface (heterogeneous nucleation), where nucleation may preferentially occur depending on the material system”

Page 9: “... or liquid/substrate ...”

Comment: (R3.3) Since the author mainly studied the heterogeneous nucleation (from scratch tip) and unidirectional growth of cylindrical crystals, the plots in Figures 1c and 1d should be derived for cylindrical rather than spherical nuclei.

Answer: We agree that the actual crystal morphology in our system, particularly the unidirectional domains, is more appropriately described by anisotropic or cylindrical geometries rather than idealized spherical nuclei. However, the classical nucleation theory (CNT) is employed to capture the general dependence of the nucleation barrier on cluster size and interfacial energy. The use of a spherical nucleus represents a standard approximation in CNT, which allows an analytical description of the relationship between critical radius and concentration. Importantly, the key conclusions of our model rely on the relative trends of critical radius and nucleation barrier across different environments (bulk, interface, cusp), rather than the exact geometric shape of the nucleus. While adopting a cylindrical or anisotropic nucleus modifies the geometric prefactors, it would not change the fundamental dependence of the nucleation condition on supersaturation.

We have clarified this point in the revised manuscript, noting that the spherical approximation is used for conceptual and analytical simplicity.

Page 7: “A spherical approximation of the cluster is used for conceptual and analytical simplicity. Although the actual crystal growth may deviate from an ideal spherical geometry, the key conclusions of the model rely on the relative evolution of the nucleation barrier and critical radius rather than the exact nucleus geometry.”

Comment: (R3.4) Since the crystal growth induced by scratching is highly anisotropic, the author should specify the alignment condition employed for GIWAXS measurements. e.g. whether the direction of the incident X-ray is parallel or perpendicular to the scratch line. Considering the length of the crystal grown from scratch (a few hundred μm) is very similar to the width of the X-ray beam (1mm), I think that the incident X-ray beam must be parallel and overlap with the scratch to ensure that the crystals sampled by the X-ray are indeed the UDs rather than the LDs around it.

Answer: In our GIWAXS measurements, the X-ray beam was positioned to cover the UD region. While the beam size (~1 mm) is larger than the width of individual domains, the UDs extend continuously along the scratch line about two centimeters, resulting in a dominant contribution from the aligned domains within the probed area. This configuration ensures that the scattering signal is dominated by the UDs rather than surrounding LD regions.

We have clarified this experimental detail in the revised Supporting Information.

Page 16 (SI): "For the film with unidirectional domains, the X-ray beam was positioned to overlap with the UD region, ensuring that the scattering signal is dominated by the aligned domains."

Comment: (R3.5) The author should also report the anisotropy in FET mobilities when the channel is parallel or perpendicular to the 2direction of UD growth.

Answer: We agree that anisotropic charge transport is expected in films with UD structures, as the reduction of grain boundaries occurs mainly along the growth direction.

In our device configuration, the scratch line is introduced prior to electrode deposition, and the transistor channel is positioned adjacent to the scratched region to preserve the integrity of the film morphology. As illustrated in Figure S20, to probe transport perpendicular to the UD growth direction, the scratch would need to be oriented across the channel. However, this would inevitably place the scratch line within the channel region, disrupting the film continuity and introducing additional defects that would dominate the transport behavior.

As a result, a direct comparison between parallel and perpendicular transport directions cannot be achieved. We have added the device geometry with the three different film morphologies in the revised manuscript.

Page 21: "The device geometry with the three film morphologies is shown in **Figure S20**."

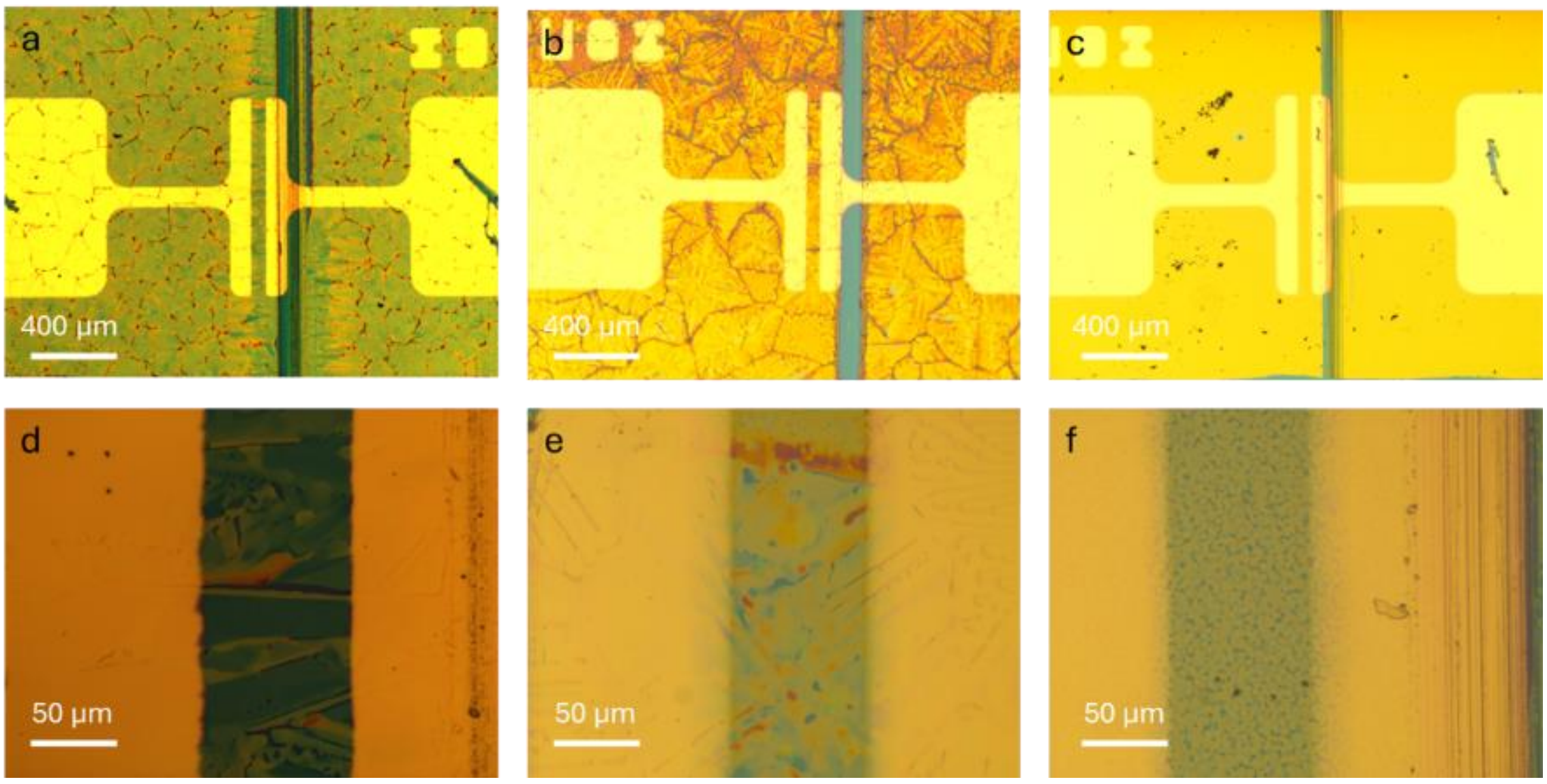

Figure S20. Device geometry and scratch configuration with (a,d) UD, (b,e) LD and (c,f) SD $(PEA)_2SnI_4$ film morphologies. The channel is positioned adjacent to the scratch to preserve film continuity.

Reviewer: 4

Comment: A strategy is introduced to separate crystal crystallization and growth in this work, and a time-defined induced nucleation model is constructed to achieve quantitative analysis of heterogeneous crystals and tailor the crystallinity of thin film based on solution-processed. This article effectively combines theoretical calculations with experimental results, and demonstrates the universality of the proposed model by replacing different material systems. However, this work employs relatively limited microscopic characterization techniques for analyzing crystallization morphology. Commonly used methods such as SEM and TEM were not utilized, which constrains the depth and comprehensiveness of the experimental results. Overall, this is a good article that provides a relatively universal and quantitative model for heterogeneous nucleation, which is of great help for future regulation of thin film growth. In conclusion, I recommend that this article be published after minor revisions.

Answer: We thank the reviewer for the positive and constructive assessment of our work, and for recognizing the significance and generality of the proposed model. In response to the reviewer's comments, we have strengthened the manuscript by incorporating additional microscopic characterization (SEM), including multi-scale analysis across different material systems, and by adding new results on multi-step spin-coating. We have also clarified the theoretical model and its applicability. We believe these revisions improve both the depth and clarity of the study. Our point-by-point responses to the reviewer's comments are provided below.

Comment: (R4.1) This work mainly introduces three heterogeneous nucleation regions (bulk, cusp and interface(gas/liquid)), but besides these regions, the wet film and substrate are also a heterojunction interface. Why is this interface not discussed here? Furthermore, has there been any attempt to use different types of substrates for experiments (such as rough substrates and relatively smooth substrates)? If possible, please provide relevant experimental and theoretical analysis data, thank you!

Answer: We agree that the wet film/substrate interface represents an important heterojunction that can influence the nucleation behavior. In our experiments, different substrates including silicon wafers (transistor application), glass (optical microscopy), and quartz (terahertz characterization) were used for various measurements, and consistent LD-UD-SD morphological evolution was observed across all cases.

We note that the dominant nucleation pathway may vary depending on the relative surface energies of the system. In some cases, nucleation is preferentially initiated at the air/liquid interface, while in others it may occur at the substrate/liquid interface. However, regardless of the specific interface involved, the nucleation process is governed by the same fundamental criterion based on the relation between cluster size and critical radius. Importantly, the mechanically induced nucleation in the cusp region provides the lowest nucleation barrier due to the maximized interfacial contact, and therefore dominates the induced nucleation process

in all cases. As a result, the overall crystallization pathway and the validity of our model remain unchanged.

To clarify this point, we have added a discussion in the revised manuscript, explicitly addressing the role of different interfaces.

Figure 1b: 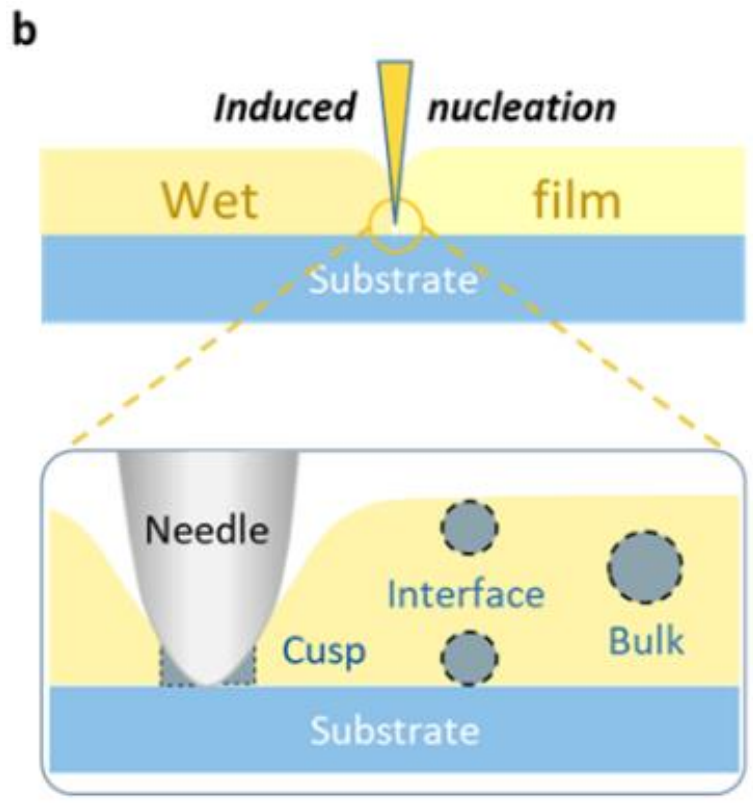

Page 6: "... or liquid/substrate ..."

Page 7: "The nucleation sites can be located in the bulk (homogeneous nucleation) the liquid/air or liquid/substrate interface (heterogeneous nucleation), where nucleation may preferentially occur depending on the material system"

Page 9: "... or liquid/substrate ..."

Comment: (R4.2) This work mainly discusses one-step spin-coating. However, in the actual preparation process, multi-step spin-coating is also a very common spin-coating mode. If possible, please supplement the relevant experimental data and theoretical analysis of multi-step spin-coating, thank you!

Answer: We agree that multi-step spin-coating is widely used in practical thin film fabrication and may introduce more complex solvent evaporation dynamics compared to the one-step process discussed in the original manuscript. In response, we have performed additional experiments using a two-step spin-coating process (first 2000 rpm for 15 s followed by 4000 rpm). The corresponding results have been added to the Supporting Information (Figure S12) and briefly discussed in the main text.

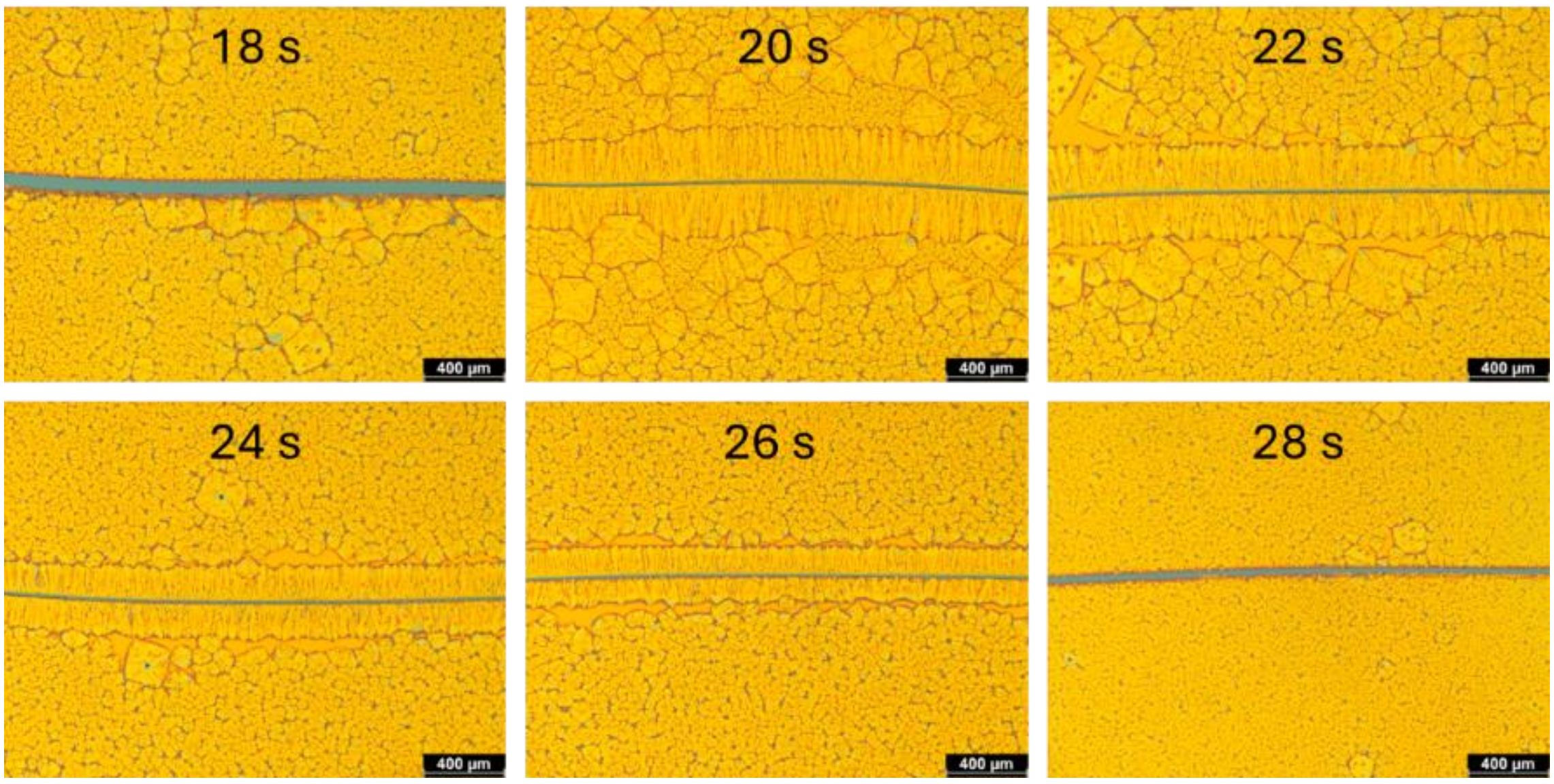

Figure S12. Optical micrographs of $(PEA)_2SnI_4$ films at C^{init} of 0.1M deposited in a two-step spin-coating process: first 2000 rpm for 15 s and followed by 4000 rpm for different spin-coating times varied from 18 s to 28 s. The scale bar is 400 µm.

As shown in Figure S12, the two-step process exhibits a crystallization behavior consistent with the one-step case. Specifically, the domain size decreases progressively with increasing processing time, following the same LD → UD → SD morphological evolution observed in the one-step spin-coating (Figure S9). This confirms that the overall crystallization pathway remains unchanged. Notably, we observe that the onset of UD nucleation occurs at an earlier time compared to the one-step process. In addition, the time interval (UD processing window) is compressed.

We attribute these differences to the increased solvent evaporation rate during the second high-speed spinning step (4000 rpm), which accelerates the increase of concentration in the wet film. As a result, the wet film reaches the critical concentration for induced nucleation (C_i) earlier, and the subsequent evolution from C_i to C_{ss} occurs within a shorter time window. Importantly, these observations are fully consistent with our model, in which the crystallization behavior is governed by the effective concentration window (C_i to C_{ss}) rather than the specific spin-coating protocol. The multi-step spin-coating process modifies the temporal profile of concentration evolution but does not alter the fundamental crystallization kinetics. These results further demonstrate the general applicability of our model under more complex processing conditions.

We have added a discussion in the revised manuscript to clarify that the proposed time-defined induced nucleation model can be generalized to multi-step spin-coating.

Page 14: "To further examine the generality of the proposed model under more complex processing conditions, we extended the analysis to a two-step spin-coating process, as shown in **Figure S12**. Despite the staged spin-coating protocol, the crystallization behavior exhibits a similar morphological evolution from LD to UD and finally to SD, consistent with the one-step process. Notably, the onset of the UD formation occurs at an earlier time, accompanied by a compressed processing window Δt^s for the UD regime compared to the one-step process

(**Figure S9**). This behavior is attributed to the increased solvent evaporation rate during the second high-speed spinning step, which accelerates the concentration increase in the wet film. As a result, the wet film reaches C_i earlier, and the transition from C_i to C_{ss} proceeds within a shorter time interval. These observations are fully consistent with the model, independent of the specific spin-coating protocol. This result further highlights the robustness of the proposed crystallization kinetics and its applicability to more realistic multi-step processing conditions."

Comment: (R4.3) The characterization of grains in this article is too macroscopic. In addition to macroscopic morphology, probing the microscopic properties of grains is highly valuable. For example, acquiring surface morphology and cross-sectional images of the films using SEM would provide important insights. If possible, please supplement relevant experimental data, thank you!

Answer: We agree that microscopic techniques such as SEM can provide valuable insights into the grain structure and film morphology. In response, we have performed additional SEM measurements for representative films across different material systems, including $(PEA)_2SnI_4$, $(MeOPEA)_{1.8}Cs_{0.1}SnI_{3.9}$, $MAPbI_3$, TIPS-pentacene and NaH_2PO_4. The corresponding results have been added to the Supporting Information (Figure S2 to S6).

The SEM images clearly resolve the distinct morphological features associated with the three crystallization regimes. Specifically, LD exhibits extended crystalline regions, UD shows pronounced anisotropic growth with aligned domain structures, and SD consists of densely packed grains. The microscopic observations are fully consistent with the macroscopic optical images presented in the main text.

Regarding the suggestion of cross-sectional SEM images, we would like to clarify that the primary focus of this work is on in-plane crystallization kinetics, particularly the lateral growth of domains governed by diffusion and concentration evolution. The proposed model quantitatively describes the evolution of domain size and morphology in the lateral direction, which is directly captured by the surface morphology. Since the cross-sectional structure mainly reflects the film thickness and vertical stacking, which are not the key variables in the present model, we believe that cross-sectional SEM would provide limited additional insight into the nucleation and growth mechanisms addressed in this study.

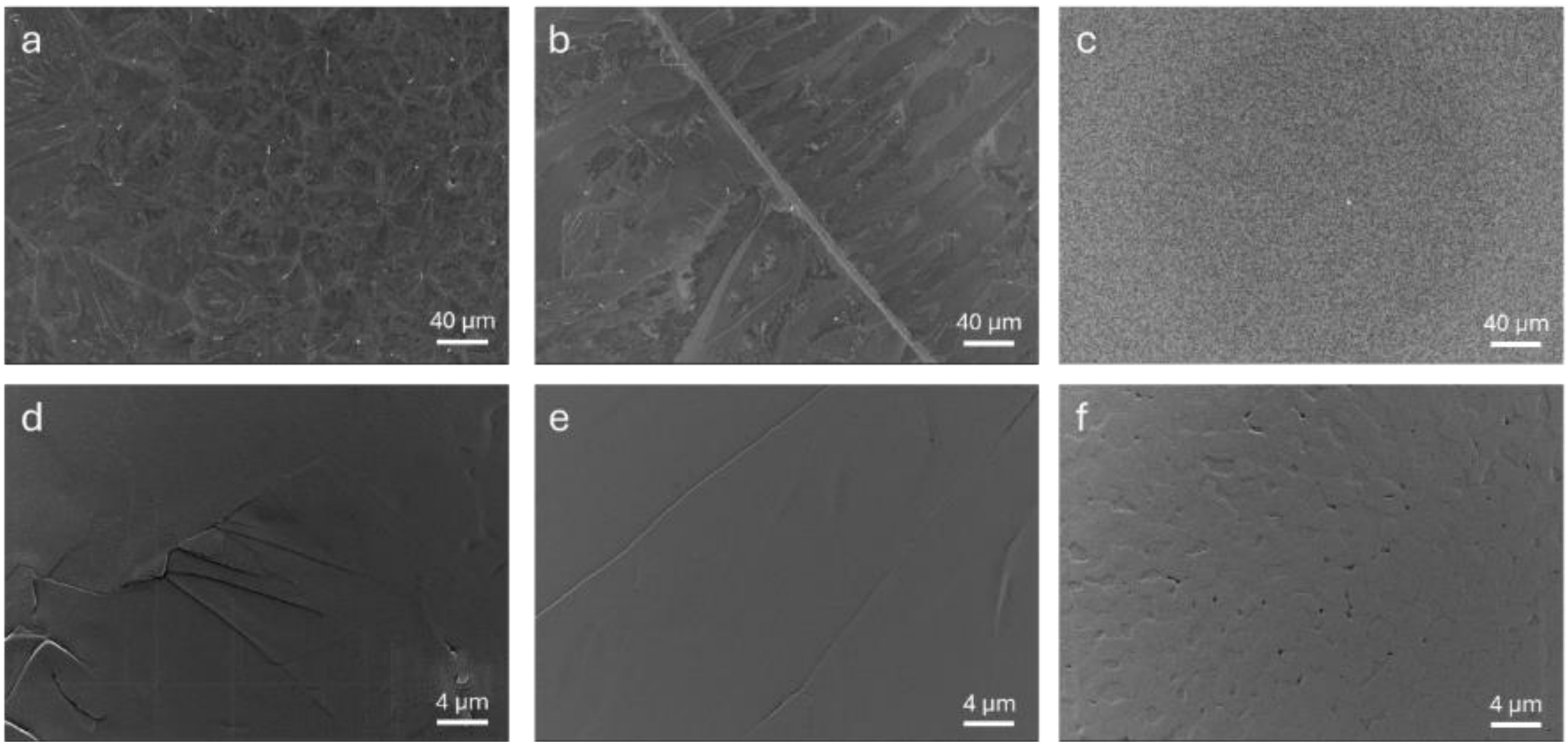

Figure S2. Scanning electron microscopy images of $(PEA)_2SnI_4$ films with (a,d) LD, (b,e) UD and (c,f) SD.

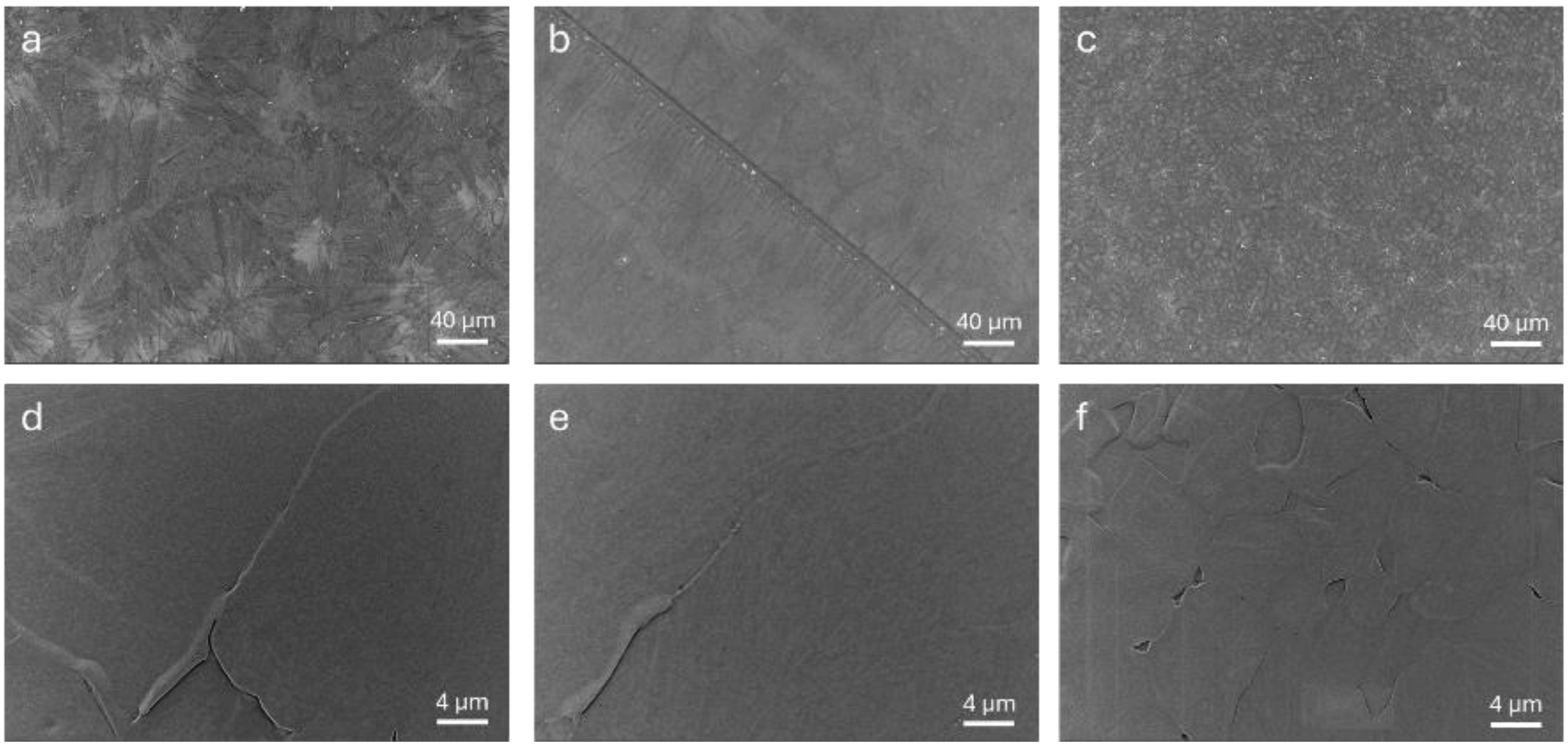

Figure S3. Scanning electron microscopy images of $(MeOPEA)_{1.8}Cs_{0.1}SnI_{3.9}$ films with (a,d) LD, (b,e) UD and (c,f) SD.

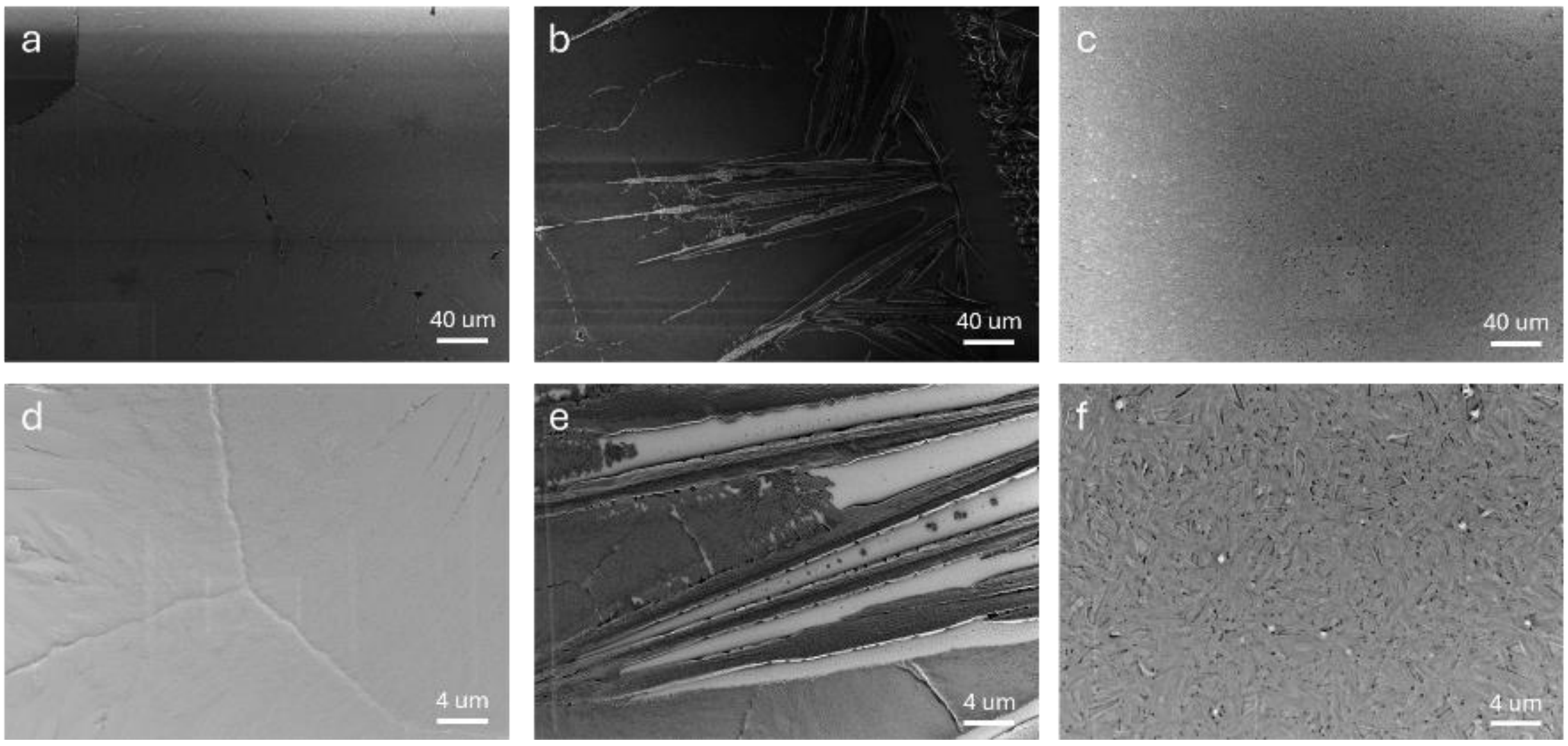

Figure S4. Scanning electron microscopy images of $MAPbI_3$ films with (a,d) LD, (b,e) UD and (c,f) SD.

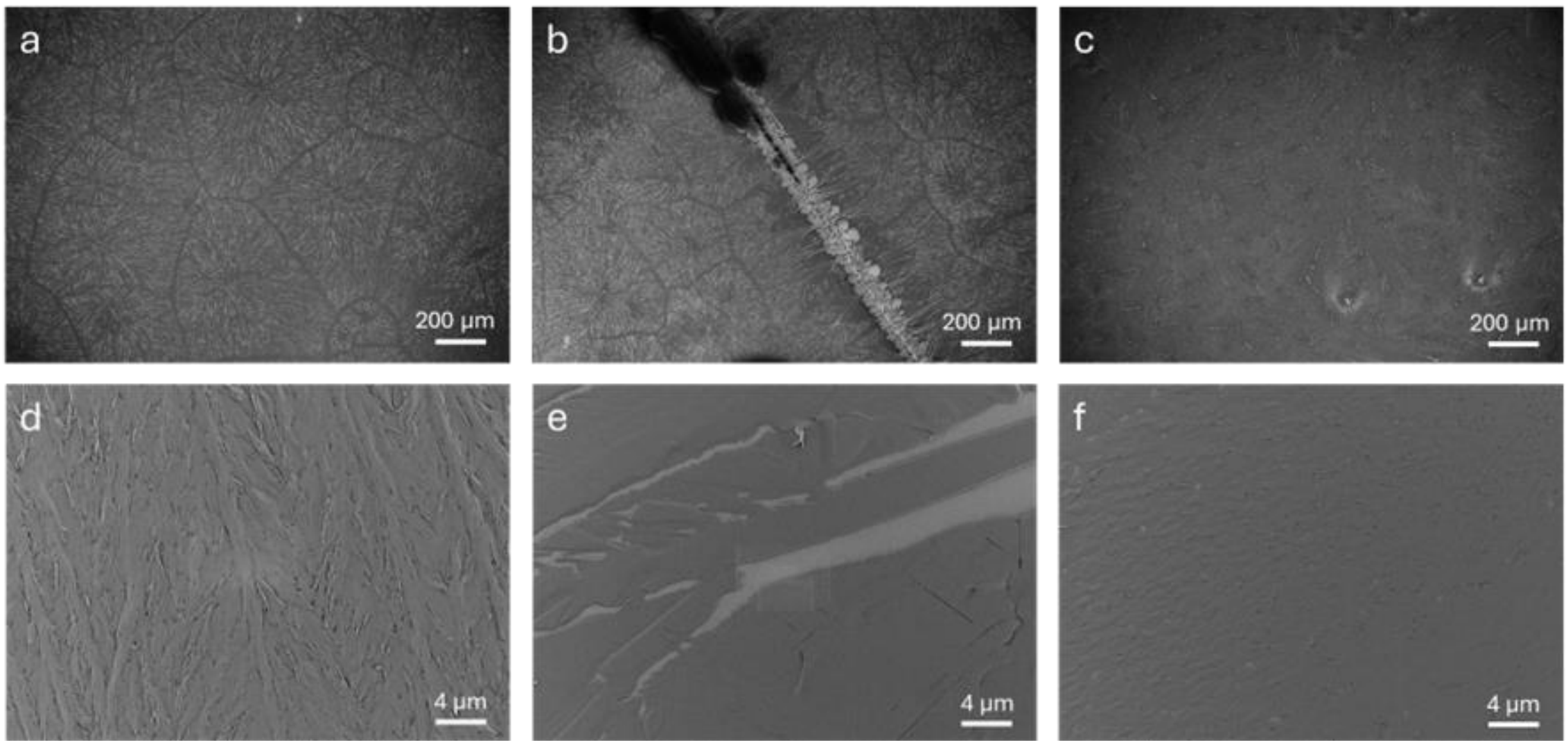

Figure S5. Scanning electron microscopy images of TIPS-pentacene films with (a,d) LD, (b,e) UD and (c,f) SD.

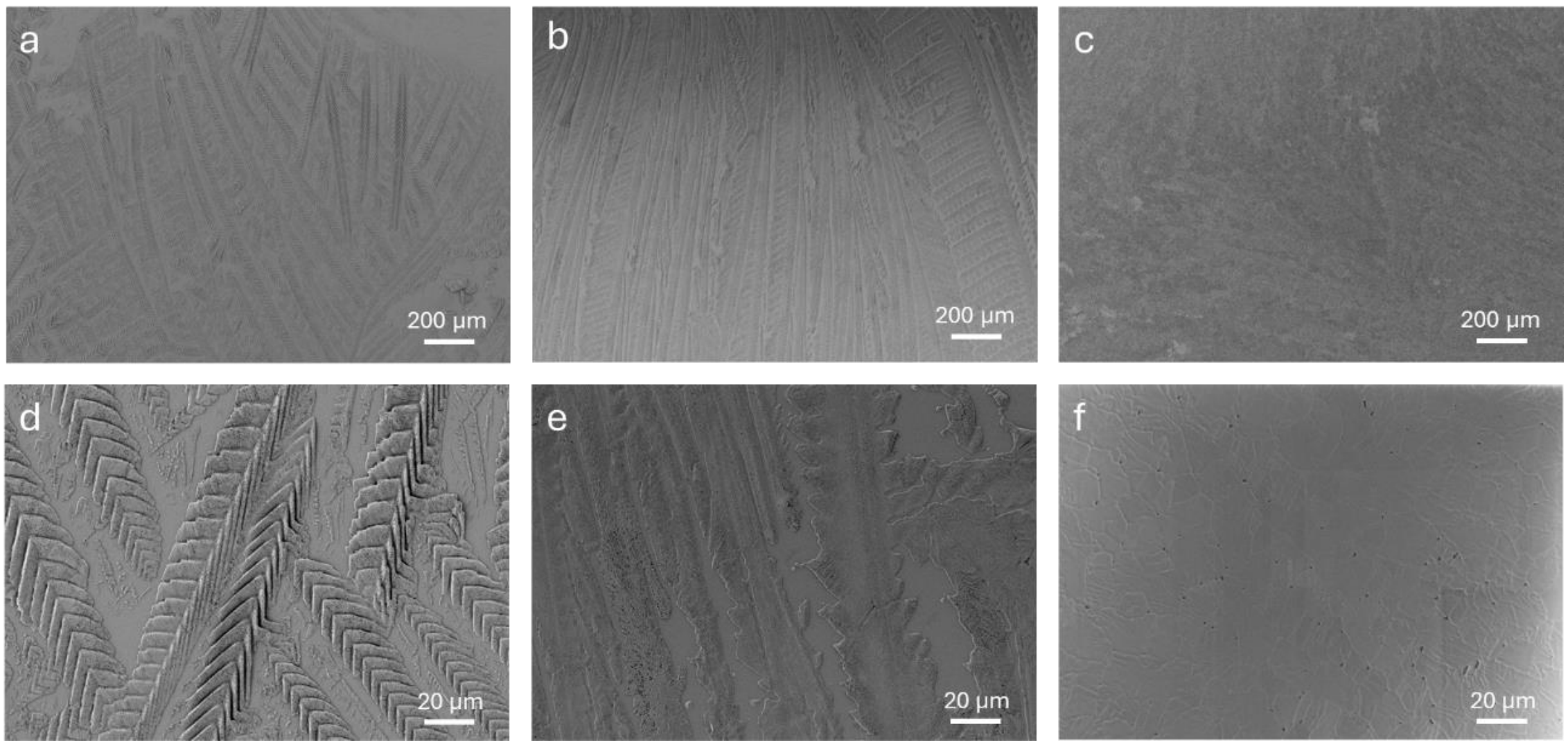

Figure S6. Scanning electron microscopy images of NaH_2PO_4 films with (a,d) LD, (b,e) UD and (c,f) SD.

We have added a discussion in the revised manuscript to incorporate these findings.

Page 10: "Scanning electron microscopy images further confirm that the distinct LD, UD, and SD morphologies are preserved at the microscale, in agreement with the macroscopic optical images (**Figure S2** to **S6**). We note that the absolute domain sizes associated with the LD, UD, and SD regimes can vary between materials while still preserving the characteristic morphology evolution."

Comment: (R4.4) Many of the contents in this work involve theoretical calculations and the application of various computational models. If possible, please briefly explain the role and applicable conditions of the model using the corresponding calculations. This will greatly help readers understand the content of the article, thank you!

Answer: We agree that a clear presentation of the role and applicability of the model is important for readers. In the original manuscript, the applicability conditions of the diffusion-based growth model were already defined through three simplifying assumptions, namely: (i) an effectively infinite substrate relative to the UD dimensions, (ii) termination of UD growth upon reaching C_{ss}, and (iii) spatially uniform solvent evaporation during spin-coating and annealing.

However, we acknowledge that these conditions were primarily introduced in the context of model derivation and may not have been sufficiently emphasized from a conceptual perspective. In response, we have revised the manuscript to make the role and applicability of the model more explicit. Specifically, we clarify that the model describes systems in which crystallization is governed by evaporation-driven concentration evolution and diffusion-limited growth. The proposed model consists of two key components: (i) a nucleation model based on classical nucleation theory, which defines the critical concentrations (C_i and C_{ss}) governing the onset

and end of induced nucleation, and (ii) a diffusion-controlled growth model that quantitatively describes domain evolution after nucleation.

These clarifications have been incorporated into the revised manuscript to improve the understanding of the model and its physical implications.

Page 15: “As a summary, the proposed model consists of two key components: (i) a nucleation model based on CNT, which defines the critical concentrations (C_i and C_{ss}) governing the onset and end of induced nucleation, and (ii) a diffusion-controlled growth model that quantitatively describes domain evolution after nucleation. The applicability of this framework relies on systems where the concentration evolution is governed by solvent evaporation and crystal growth is limited by mass transport. Under these conditions, the crystallization pathway can be described by the parameter pair of R and S_{Si}, which links nucleation and growth across different processing conditions.”
