# Supplementary material for: Universal crystallization kinetics of solution-processed semiconductors
Source: Nat Commun. 2026 Oct 14;17:10785. doi: 10.1038/s41467-026-78354-7 (PMC13669315; doi:10.1038/s41467-026-78354-7)
Supplement: Supplementary file 2 [file 41467_2026_78354_MOESM1_ESM.docx]

**Universal Crystallization Kinetics of Solution-Processed Semiconductors**

Zhitian Ling^1^, Mateusz Brzezinski^1,2^, Shuanglong Wang^1^, Konstantinos Bidinakis^1^, Lei Gao^1^, Chongyao Li^1^, Xin Zhou^1^, Hai I. Wang^1,3^, Stefan A.L. Weber^1^, Paul W.M. Blom^1^, Wojciech Pisula^1,4*^ and Tomasz Marszalek^1,4*^

^1^ Max Planck Institute for Polymer Research, Ackermannweg 10, 55128 Mainz, Germany

^2^ Institute of Physical Chemistry Polish Academy of Sciences, Kasprzaka 44/52, 01-224 Warsaw, Poland

^3^ Nanophotonics, Debye Institute for Nanomaterials Science, Utrecht University, Princetonplein 1, 3584 CC, Utrecht, The Netherlands

^4^ Department of Molecular Physics, Faculty of Chemistry, Lodz University of Technology, Zeromskiego 116, 90-924 Lodz, Poland

^*^To whom correspondence should be addressed.

**Table of contents**

List of Abbreviation for the Model	3

Model	6

Figures	14

Tables	32

Supplementary references	34

**List of Abbreviation for the Model**

| **Abbreviation** | **Unit** | **Physical meaning** |
| --- | --- | --- |
| *A* | m^2^ | Mass transfer area |
| *C* | mol/L | Concentration |
| *C*_i_ | mol/L | Critical concentration for mechanically induced nucleation |
| *C*_n_ | mol/L | Concentration for the spontaneous interfacial nucleation |
| *C*_s_ | mol/L | Solubility |
| *C*_ss_ | mol/L | Critical supersaturation for spontaneous bulk nucleation |
| *C*_0_ | mol/L | Concentration of the induced nucleation |
| *C*^init^ | mol/L | Initial concentration of the precursor solution |
| Δ*C*_A_ | mol/L | Concentration difference between crystal surface and bulk |
| *C’* | mol/L | Revised concentration |
| *D* | m | Average thickness of dry film |
| Δ*G* | J | Change in Gibbs free energy |
| Δ*G*_V_ | J | Change in surface Gibbs free energy |
| Δ*G*_S_ | J | Change in volume Gibbs free energy |
| *k*_c_ | m/s | Mass transfer coefficient |
| *k*_b_ | J/K | Boltzmann constant |
| *L* | m | Time-dependent UD length during annealing |
| *L*^final^ | m | Final UD length after annealing |
| *L*final max | m | Maximum *L*^final^ |
| *L*_normal_ | - | *L*/ *L*^final^ |
| *L*final normal | - | *L*^final^/ *L*final max |
| *L*^subs^ | m | Length of the substrate |
| *M* | kg/mol | Molar mass of the solute |
| *m*_eva_ | kg | Evaporation mass of solvent |
| *m*_i_ | kg | Mass of solute for the wet film at *C*_i_ |
| *m*_UD_ | kg | Mass of UD |
| *n*_i_ | mol | Mole number of solute for the wet film at *C*_i_ |
| *n*_diff_ | mol | Mole number of diffusion |
| *n*_UD_ | mol | Mole number of UD |
| *n*_A_ | mol/s | Mole number transfer rate |
| *Q*_spin_ | kg/(m^2^s) | Areal evaporation fluxes during spin-coating |
| *Q*_anneal_ | kg/(m^2^s) | Areal evaporation fluxes during annealing |
| *r* | m | Radius of cluster |
| *r*^*^ | m | Critical radius for the nucleation |
| *R* | - | *C*_s_/ *C*_ss_ |
| *S* | m^2^ | Area of the substrate |
| *S*_S_ | - | *C*/ *C*_s_ |
| *S*_Si_ | - | *C*_i_/ *C*_s_ |
| *S*_S0_ | - | *C*_0_/ *C*_s_ |
| *t*^s^ | s | Spin-coating time |
| *t*s 0 | s | Spin-coating time when *C* = *C*_0_ |
| *t*s i | s | Spin-coating time when *C* = *C*_i_ |
| *t*s ss | s | Spin-coating time when *C* = *C*_ss_ |
| Δ*t*^s^ | s | *t*s 0 - *t*s i |
| Δ*t*s max | s | *t*s ss - *t*s i |
| Δ*t*s normal | - | Δ*t*^s^/ Δ*t*s max |
| *t*^a^ | s | Annealing time |
| *t*a 0 | s | Annealing time when *C* = *C*_0_ |
| *t*a i | s | Annealing time when *C* = *C*_i_ |
| *t*a ss | s | Annealing time when *C* = *C*_ss_ |
| Δ*t*^a^ | s | *t*a ss - *t*a 0 |
| Δ*t*a max | s | *t*a ss - *t*a i |
| Δ*t*a normal | - | (*t*^a^ - *t*a 0)/ Δ*t*^a^ |
| *T* | K | Absolute temperature |
| *V*_eva_ | m^3^ | Evaporation volume of the solvent |
| *V*_i_ | m^3^ | Volume of the wet film at *C*_i_ |
| *V*_UD_ | m^3^ | Volume of UD |
| *V*_m_ | m^3^/mol | Molar volume of the molecule in the cluster |
| *W* | m | Width of the substrate |
| *α* | - | Concentration coefficient |
| *γ* | J/m^2^ | Surface free energy per unit area of the cluster |
| *ω* | rpm | Spin-coating speed |
| $\rho$_solvent_ | kg/m^3^ | Density of the solvent |
| $\rho$_solute_ | kg/m^3^ | Density of the solute |

**Model**

The nucleation and growth is affacted by the concentration of wet film, which changes during processing. The process is divided into two main stages: spin-coating and annealing. The change of the concentration is dominated by evaporation of the solvent during both stages. The evaporation rate varies between the two stages. The model is established under three simplifying assumptions, which are supported by both the experimental design and direct observations as: (1) Infinite substrate assumption: The dimension of the substrate is orders of magnitude larger than the characteristic size of the unidirectional domains (typically a few hundred micrometers). Therefore, the substrate can be reasonably treated as effectively infinite for the purpose of modelling the UD growth. (2) Termination of UD growth at *C*_ss_: This assumption is based on the classical nucleation theory, in which reaching the critical concentration *C*_ss_ leads to the onset of spontaneous bulk nucleation. Experimentally, we directly observe a rapid emergence of numerous nuclei across the film at late stages (see Figure 3 and Supplementary Movie 1 to 4), which interrupts further UD growth. This observation is consistent with the model assumption that UD growth ceases once the concentration reaches *C*_ss_. (3) Spatially uniform evaporation: During spin-coating and annealing, the experiments were conducted under controlled and uniform environmental conditions (e.g. atmosphere and pressure), ensuring consistent solvent evaporation across the film. In addition, no significant spatial variation in morphology is observed prior to nucleation, supporting the approximation of spatially uniform evaporation at the scale relevant for the UD growth.

First, the concentration change during spin-coating is discussed. The areal evaporation flux during spin-coating is defined as *Q*_spin_. The area of substrate is defined as *S* and the evaporation mass within the time interval Δ*t*^s^ (*t*^s^-*t*s i) as *m*_eva_. From the definition, we know that

$$\begin{aligned} m_{\mathrm{eva}}=Q_{\mathrm{spin}}S\Delta t^{s}\#\left( S1 \right) \end{aligned}$$

With the density of the solvent $\rho$_solvent_, the evaporation volume (*V*_eva_) during Δ*t*^s^ can be expressed by

$$\begin{aligned} V_{\mathrm{eva}}=\frac{m_{\mathrm{eva}}}{\rho_{\mathrm{solvent}}}\#\left( S2 \right) \end{aligned}$$

We define at *C*_i_ the volume of the wet film as *V*_i_, the mole number of solute as *n*_i_, the molar mass of solute as *M*, and the mass of solute as *m*_i_. With the average thickness of dry film *d* and the density of solute $\rho$_solute_ the following expressions are obtained

$$\begin{aligned} m_{i}=Sd\rho_{\mathrm{solute}}\#\left( S3 \right) \end{aligned}$$

$$\begin{aligned} n_{i}=\frac{m_{i}}{M}\#\left( S4 \right) \end{aligned}$$

$$\begin{aligned} V_{i}=\frac{n_{i}}{C_{i}}\#\left( S5 \right) \end{aligned}$$

Combination of Equations S3, S4 and S5 results in

$$\begin{aligned} V_{i}=\frac{Sd\rho_{\mathrm{solute}}}{MC_{i}}\#\left( S6 \right) \end{aligned}$$

The relation between concentration and rotation time is given as

$$\begin{aligned} C=\frac{n_{i}}{V_{i}-V_{\mathrm{eva}}}=\frac{C_{i}}{1-\frac{V_{\mathrm{eva}}}{V_{i}}}\#\left( S7 \right) \end{aligned}$$

Combination with Equation S2 and S6 leads to

$$\begin{aligned} C=\frac{C_{i}}{1-\frac{Q_{\mathrm{spin}}MC_{i}}{\rho_{\mathrm{solvent}}\rho_{\mathrm{solute}}d}\Delta t^{s}}=\frac{C_{i}}{1-\frac{Q_{\mathrm{spin}}C_{i}}{\alpha}\Delta t^{s}}\#\left( S8 \right) \end{aligned}$$

where *α* is the concentration coefficient which is a constant value for the same initial concentration.

In the second step, the change of the wet film concentration during the annealing stage is described. In this stage the concentration change is mainly attributed to the evaporation of solvent which leads to a concentration increase and diffusion of solute which contributes to the concentration decrease. The concentration change is dominated by solvent evaporation and the change mechanism is similar to the spin-coating stage with the only difference in the evaporation rate. For the annealing stage, the areal evaporation flux during annealing is defined as *Q*_anneal_. With the same appraoch as before, the relation between the evaporation time and the concentration is defined as below

$$\begin{aligned} C=\frac{C_{\mathrm{ss}}}{1+\frac{Q_{\mathrm{anneal}}C_{\mathrm{ss}}}{\alpha}\Delta t^{a}}\#\left( S9 \right) \end{aligned}$$

It should be noted that Equation S8 is written in the forward direction from *C*_i_ to *C*_0_ for the spin-coating process, whereas Equation S9 is written in the reverse direction from *C*_ss_ back to *C*_0_ during annealing. Therefore, the evaporated solvent volume is subtracted in Equation S8 but added back in Equation S9. The schematic diagram of these two process is shown in Figure 2b. The initial concentration during induced nucleation is marked as *C*_0_. Combining Equations S8 and S9, the change of concentration during whole process including spin-coating and annealing is described as below

$$\begin{aligned} C=\left\{ \begin{aligned} \frac{C_{i}}{1-\frac{Q_{\mathrm{spin}}C_{i}}{\alpha}(t^{s}-t_{i}^{s})}, t_{i}^{s}\leq t^{s}\leq t_{0}^{s} (spin coating) \\ \frac{C_{\mathrm{ss}}}{1-\frac{Q_{\mathrm{anneal}}C_{\mathrm{ss}}}{\alpha}(t^{a}-t_{\mathrm{ss}}^{a})}, t_{0}^{a}\leq t^{a}\leq t_{\mathrm{ss}}^{a} (annealing) \end{aligned} \right.\#(S10)\# \end{aligned}$$

*C*_0_ can be expressed for both spin-coating and annealing stages as below

$$\begin{aligned} \frac{C_{i}}{1-\frac{Q_{\mathrm{spin}}C_{i}}{\alpha}\left( t_{0}^{s}-t_{i}^{s} \right)}=C_{0}=\frac{C_{\mathrm{ss}}}{1-\frac{Q_{\mathrm{anneal}}C_{\mathrm{ss}}}{\alpha}\left( t_{0}^{a}-t_{\mathrm{ss}}^{a} \right)} \#\left( S11 \right) \end{aligned}$$

It should be noted that Δ*t*^s^ = *t*s 0 - *t*s i and Δ*t*^a^ = ta ss - *t*a 0. Taking the reciprocal form of Equation S11 gives the relation between Δ*t*^s^ and Δ*t*^a^ as

$$\begin{aligned} \frac{1}{C_{i}}-\frac{Q_{\mathrm{spin}}}{\alpha}\Delta t^{s}=\frac{1}{C_{0}}=\frac{1}{C_{\mathrm{ss}}}+\frac{Q_{\mathrm{anneal}}}{\alpha}\Delta t^{a} \\ Q_{\mathrm{spin}}\Delta t^{s}+Q_{\mathrm{anneal}}\Delta t^{a}=\left( \frac{1}{C_{i}}-\frac{1}{C_{\mathrm{ss}}} \right)\alpha\#\left( S12 \right) \end{aligned}$$

From Equation S12, it is worth to note that Δ*t*s max and Δ*t*a max are obtained from two separate limiting boundary conditions, namely Δ*t*^a^ = 0 and Δ*t*^s^ = 0, respectively. The corresponding expression is

$$\begin{aligned} {\Delta t}_{\max}^{s}=\left( \frac{1}{C_{i}}-\frac{1}{C_{\mathrm{ss}}} \right)\frac{\alpha}{Q_{\mathrm{spin}}}\#\left( S13 \right) \end{aligned}$$

$$\begin{aligned} {\Delta t}_{\max}^{a}=\left( \frac{1}{C_{i}}-\frac{1}{C_{\mathrm{ss}}} \right)\frac{\alpha}{Q_{\mathrm{anneal}}}\#\left( S14 \right) \end{aligned}$$

This simple model describes the change of the concentration in the wet film during annealing without considering diffusion. For taking the diffusion into account, a revised concentration *C’* is introduced.

Figure M1. Schematic illustration of the geometries used for (a) solute diffusion and (b) mass transfer theory.

**Figure M1a** shows the geometry important for the solute diffusion. *L*^subs^ and *W* represent the length and width of substrate, while *L* is assigned to the length of UDs. It is defined that all solute diffuses to form the UDs. Therefore, the mole number of diffusion (*n*_diff_) is equal to the mole number of UD (*n*_UD_). The mass and volume of UDs is defined as *m*_UD_ and *V*_UD_, respectively leading to

$$\begin{aligned} V_{\mathrm{UD}}=2LdW\#\left( S15 \right) \end{aligned}$$

$$\begin{aligned} m_{\mathrm{UD}}={\rho_{\mathrm{solute}}V}_{\mathrm{UD}}\#\left( S16 \right) \end{aligned}$$

$$\begin{aligned} n_{\mathrm{diff}}=n_{\mathrm{UD}}=\frac{m_{\mathrm{UD}}}{M}=\frac{2LdW\rho_{\mathrm{solute}}}{M}\#\left( S17 \right) \end{aligned}$$

$$\begin{aligned} S=L^{\mathrm{subs}}W\#\left( S18 \right) \end{aligned}$$

When the diffusion is taken into account, the introduction of *C’* to the annealing process can be expressed as below

$$\begin{aligned} C'=\frac{n_{\mathrm{ss}}+n_{\mathrm{diff}}}{V_{\mathrm{ss}}+V_{\mathrm{eva}}\left( \Delta t^{a} \right)}\#\left( S19 \right) \end{aligned}$$

$$\begin{aligned} C'=\frac{C_{\mathrm{ss}}}{1+\frac{Q_{\mathrm{anneal}}C_{\mathrm{ss}}}{\alpha}\Delta t^{a}}(1+\frac{2L}{L^{\mathrm{subs}}})\#\left( S20 \right) \end{aligned}$$

To simplify this model, we assume that the substrate is infinite resulting in 2*L*/*L*^subs^ to be zero. Compared with Equation S9 and S20, we can obtain

$$\begin{aligned} C^{'}=C\#\left( S21 \right) \end{aligned}$$

It means that with the assumption of infinite substrate, the concentration change is dominated by evaporation instead of growth.

Based on the above concepts, the UD growth can be described in the following way. The mass transfer coefficient (*k*_c_) is introduced to quantify the mass transfer between phases and relates with the mole number transfer rate (*n*_A_), the mass transfer area (*A*), and the concentration change (Δ*C*_A_) as shown in **Figure M1b** and described by the following relation

$$\begin{aligned} k_{c}=\frac{n_{A}}{A{\Delta C}_{A}}\#\left( S22 \right) \end{aligned}$$

Here, the mass transfer coefficient *k*_c_ reflects the combined effects of diffusion, solvent evaporation, and concentration evolution during the crystallization process. We define d*n*_A_ (*mol*) as the mole number transferred in unit time (d*t*^a^), d*m* as the mass transfer in unit time, and d*L* as the length grown in unit time

$$\begin{aligned} n_{A}=\frac{dn}{dt^{a}}\#\left( S23 \right) \end{aligned}$$

$$\begin{aligned} dn=\frac{dm}{M}\#\left( S24 \right) \end{aligned}$$

$$\begin{aligned} dm=\rho_{\mathrm{solute}}AdL\#\left( S25 \right) \end{aligned}$$

The combination of Equations S22 to S25 results in

$$\begin{aligned} k_{c}=\frac{\rho_{\mathrm{solute}}AdL}{AM{\Delta C}_{A}dt^{a}}=\frac{\rho_{\mathrm{solute}}dL}{M{\Delta C}_{A}dt^{a}}\#\left( S26 \right) \end{aligned}$$

$$\begin{aligned} \frac{\rho_{\mathrm{solute}}}{Mk_{c}}dL={\Delta C}_{A}dt^{a}\#\left( S27 \right) \end{aligned}$$

In our case, Δ*C*_A_ is the concentration difference between the concentration at the surface of UD (*C*_s_) and the concentration of wet film (*C*)

$$\begin{aligned} {\Delta C}_{A}=C-C_{s}\#\left( S28 \right) \end{aligned}$$

The combination of Equation S10, S27 and S28 leads to the following expression

$$\begin{aligned} \frac{\rho_{\mathrm{solute}}}{Mk_{c}}dL=\left( C-C_{s} \right)dt^{a}, t_{0}^{a}\leq t^{a}\leq t_{\mathrm{ss}}^{a} \#\left( S29 \right) \end{aligned}$$

$$\begin{aligned} \frac{\rho_{\mathrm{solute}}}{Mk_{c}}dL=(\frac{C_{\mathrm{ss}}}{1-\frac{Q_{\mathrm{anneal}}C_{\mathrm{ss}}}{\alpha}(t^{a}-t_{\mathrm{ss}}^{a})}-C_{s})dt^{a}\#\left( S30 \right) \end{aligned}$$

This is the differential equation describing the UD growth by our model. To describe the relation between UD length and rotation time, the differential is transferred into the following integral

$$\begin{aligned} \int_{0}^{L^{\mathrm{final}}} \frac{\rho_{\mathrm{solute}}}{Mk_{c}}dL=\int_{t_{0}^{a}}^{t_{\mathrm{ss}}^{a}} (\frac{C_{\mathrm{ss}}}{1-\frac{Q_{\mathrm{anneal}}C_{\mathrm{ss}}}{\alpha}(t^{a}-t_{\mathrm{ss}}^{a})}-C_{s})dt^{a} \#\left( S31 \right) \end{aligned}$$

To describe the crystallization process, the equation is changed into the indefinite integral

$$\begin{aligned} \int_{0}^{L} \frac{\rho_{\mathrm{solute}}}{Mk_{c}}dL=\int_{t_{0}^{a}}^{t^{a}} (\frac{C_{\mathrm{ss}}}{1-\frac{Q_{\mathrm{anneal}}C_{\mathrm{ss}}}{\alpha}(t^{a}-t_{\mathrm{ss}}^{a})}-C_{s})dt^{a} \#\left( S32 \right) \end{aligned}$$

Equation S33 is obtained after the integration of Equation S31 as

$$\begin{aligned} \frac{Q_{a\mathrm{nneal}}}{{\rho_{\mathrm{solvent}}k}_{c}d}L^{\mathrm{final}}=ln(1+\frac{Q_{\mathrm{anneal}}C_{\mathrm{ss}}}{\alpha}\Delta t^{a})-\frac{Q_{\mathrm{anneal}}C_{s}}{\alpha}\Delta t^{a}\#\left( S33 \right) \end{aligned}$$

With the combination of Equation S12, the relation between *L*^final^ and Δ*t*^s^ is expressed as

$$\begin{aligned} \frac{Q_{\mathrm{anneal}}}{{\rho_{\mathrm{solvent}}k}_{c}d}L^{\mathrm{final}}=\ln\left( 1-\frac{Q_{\mathrm{spin}}C_{i}}{\alpha}\Delta t^{s} \right)-\left( 1-\frac{Q_{\mathrm{spin}}C_{i}}{\alpha}\Delta t^{s} \right)\frac{1}{S_{\mathrm{Si}}}+(\frac{C_{s}}{C_{\mathrm{ss}}}-ln\frac{C_{i}}{C_{\mathrm{ss}}})\#\left( S34 \right) \end{aligned}$$

*S*_Si_ is introduced as *C*_i_/*C*_s_ and from Supplementary Figure 3b, maximum *L*^final^ (*L*final max) can be obtained when Δ*t*^s^ is equal to zero resulting in

$$\begin{aligned} L_{\max}^{\mathrm{final}}=(-\frac{1}{S_{\mathrm{Si}}}+\frac{C_{s}}{C_{\mathrm{ss}}}-ln\frac{C_{i}}{C_{\mathrm{ss}}})\frac{{\rho_{\mathrm{solvent}}k}_{c}d}{Q_{\mathrm{anneal}}}\#\left( S35 \right) \end{aligned}$$

Combining Equations S13, S34 and S35, the relation between *L*final normal (*L*^final^/ *L*final max) and Δ*t*s normal (Δ*t*^s^/ Δ*t*s max) can be deduced as

$$\begin{aligned} (R-\ln(RS_{\mathrm{Si}})-\frac{1}{S_{\mathrm{Si}}})L_{\mathrm{normal}}^{\mathrm{final}}=\ln\left( 1-\left( 1-RS_{\mathrm{Si}} \right){\Delta t}_{\mathrm{normal}}^{s} \right)-\frac{1-\left( 1-RS_{\mathrm{Si}} \right){\Delta t}_{\mathrm{normal}}^{s}}{S_{\mathrm{Si}}}+R-\ln(RS_{\mathrm{Si}})\#\left( S36 \right) \end{aligned}$$

where relative driving force (*R*) and supersaturation index (*S*_Si_) are used to represent *C*_s_/*C*_ss_ and *C*_i_/*C*_s_, respectively. The fitting curves in Figure 2e and 2f are based on the Equation S36.

The crystallization process during annealing is expressed by Equation S37 obtained after the integration of Equation S32 as

$$\begin{aligned} \frac{Q_{\mathrm{anneal}}}{{\rho_{\mathrm{solvent}}k}_{c}d}L=-\ln\left( 1+\frac{\frac{Q_{\mathrm{anneal}}C_{\mathrm{ss}}}{\alpha}\left( t^{a}-t_{0}^{a} \right)}{1-\frac{Q_{\mathrm{anneal}}C_{\mathrm{ss}}}{\alpha}\left( t_{\mathrm{ss}}^{a}-t_{0}^{a} \right)} \right)-\frac{Q_{\mathrm{anneal}}C_{s}}{\alpha}(t^{a}-t_{0}^{a})\#\left( S37 \right) \end{aligned}$$

From Equation S11, we know that

$$\begin{aligned} \frac{Q_{\mathrm{anneal}}}{\alpha}=\frac{\frac{1}{C_{\mathrm{ss}}}-\frac{1}{C_{0}}}{t_{0}^{a}-t_{\mathrm{ss}}^{a}} \#\left( S38 \right) \end{aligned}$$

Combining Equations S37 and S38, Equation S39 is obtained as

$$\begin{aligned} \frac{Q_{\mathrm{anneal}}}{{\rho_{\mathrm{solvent}}k}_{c}d}L=-\ln\left( 1+\left( \frac{C_{0}}{C_{\mathrm{ss}}}-1 \right)\left( \frac{t^{a}-t_{0}^{a}}{t_{\mathrm{ss}}^{a}-t_{0}^{a}} \right) \right)+\frac{C_{s}}{C_{\mathrm{ss}}}(1-\frac{C_{\mathrm{ss}}}{C_{0}})\frac{t^{a}-t_{0}^{a}}{t_{\mathrm{ss}}^{a}-t_{0}^{a}}\#\left( S39 \right) \end{aligned}$$

We notice that *L*^final^ is reached when *t*^a^ is equal to *t*a ss as

$$\begin{aligned} L^{\mathrm{final}}=\frac{\frac{C_{s}}{C_{\mathrm{ss}}}-ln\frac{C_{0}}{C_{\mathrm{ss}}}-\frac{C_{s}}{C_{0}}}{\frac{Q_{\mathrm{anneal}}}{{\rho_{\mathrm{solvent}}k}_{c}d}}\#\left( S40 \right) \end{aligned}$$

Combining Equations S39 and S40, the following expression is obtained

$$\begin{aligned} (\frac{C_{s}}{C_{\mathrm{ss}}}-\ln\frac{C_{0}}{C_{\mathrm{ss}}}-\frac{C_{s}}{C_{\mathrm{ss}}}\frac{C_{\mathrm{ss}}}{C_{0}})\frac{L}{L^{\mathrm{final}}}=-\ln\left( 1+\left( \frac{C_{0}}{C_{\mathrm{ss}}}-1 \right)\left( \frac{t^{a}-t_{0}^{a}}{t_{\mathrm{ss}}^{a}-t_{0}^{a}} \right) \right)+\frac{C_{s}}{C_{\mathrm{ss}}}(1-\frac{C_{\mathrm{ss}}}{C_{0}})\frac{t^{a}-t_{0}^{a}}{t_{\mathrm{ss}}^{a}-t_{0}^{a}}\#\left( S41 \right) \end{aligned}$$

It should be also noted that Equation S41 is a general expression to describe the UD growth in any case. We use instantaneous supersaturation index (*S*_S0_) to replace *C*_0_/ *C*_s_, Δ*t*a normal to replace (*t*^a^ - *t*a 0)/ Δ*t*^a^ and *L*_normal_ to replace *L*/ *L*^final^ to transfer into Equation S41

$$\begin{aligned} \left( R-\ln(RS_{S0})-\frac{1}{S_{S0}} \right)L_{\mathrm{narmal}}=-\ln\left( 1-(1-RS_{S0}){\Delta t}_{\mathrm{normal}}^{a} \right)-(\frac{1}{S_{S0}}-R){\Delta t}_{\mathrm{normal}}^{a}\#\left( S42 \right) \end{aligned}$$

From Equation S11, *C*_0_ can be expressed when *t*^s^ is equal to *t*s 0 as following

$$\begin{aligned} C_{0}=\frac{C_{i}}{1-\frac{Q_{\mathrm{spin}}C_{i}}{\alpha}\left( t_{0}^{s}-t_{i}^{s} \right)}\#\left( S43 \right) \end{aligned}$$

When *t*s 0 is equal to *t*s ss, *C*_0_ matches to *C*_ss_ as shown in Supplementary **Figure 3a**. Hence, *C*_ss_ can be expressed as

$$\begin{aligned} C_{\mathrm{ss}}=\frac{C_{i}}{1-\frac{Q_{\mathrm{spin}}C_{i}}{\alpha}\left( t_{\mathrm{ss}}^{s}-t_{i}^{s} \right)}\#\left( S44 \right) \end{aligned}$$

Combining Equations S43 and S44, we obtain

$$\begin{aligned} \frac{1}{S_{S0}}=\frac{1}{S_{\mathrm{Si}}}-(\frac{1}{S_{\mathrm{Si}}}-R)\frac{t_{0}^{s}-t_{i}^{s}}{t_{\mathrm{ss}}^{s}-t_{i}^{s}}\#\left( S45 \right) \end{aligned}$$

To predict the UD crystallization when nucleation is induced under ideal onset conditions, the variable *C*_0_ should be fixed to *C*_i_. The Equation S42 is transferred into

$$\begin{aligned} \left( R-\ln(RS_{\mathrm{Si}})-\frac{1}{S_{\mathrm{Si}}} \right)L_{\mathrm{narmal}}=-ln\left( 1-(1-RS_{\mathrm{Si}}){\Delta t}_{\mathrm{normal}}^{a} \right)-(\frac{1}{S_{\mathrm{Si}}}-R){\Delta t}_{\mathrm{normal}}^{a}\#\left( S46 \right) \end{aligned}$$

With the *R* and *S*_Si_ parameters shown in **Table S1**, Equation S46 yields the theoretical curve in **Figure 3e** for the (PEA)_2_SnI_4_ perovskite film.

Figure M2. The relation between Δ*t*^a^ and *t*s 0 for UD films in Figure 3b to 3d.

To calculate the theoretical curve for UD films with initial concentration of 0.12 M and rotation times of 40 s, 44 s and 48 s, Equation S42 is used with

$$\begin{aligned} \frac{1}{S_{S01}}=\frac{1}{S_{\mathrm{Si}}}-(\frac{1}{S_{\mathrm{Si}}}-R)\frac{t_{01}^{s}-t_{i}^{s}}{t_{\mathrm{ss}}^{s}-t_{i}^{s}}\#\left( S47 \right) \end{aligned}$$

$$\begin{aligned} \frac{1}{S_{S03}}=\frac{1}{S_{\mathrm{Si}}}-(\frac{1}{S_{\mathrm{Si}}}-R)\frac{t_{02}^{s}-t_{i}^{s}}{t_{\mathrm{ss}}^{s}-t_{i}^{s}}\#\left( S48 \right) \end{aligned}$$

$$\begin{aligned} \frac{1}{S_{S03}}=\frac{1}{S_{\mathrm{Si}}}-(\frac{1}{S_{\mathrm{Si}}}-R)\frac{t_{03}^{s}-t_{i}^{s}}{t_{\mathrm{ss}}^{s}-t_{i}^{s}}\#\left( S49 \right) \end{aligned}$$

where *S*_Si_ and *R* are already known in **Table S1**. From Equation S12, we know that

$$\begin{aligned} t_{0}^{s}=-\frac{Q_{\mathrm{anneal}}}{Q_{\mathrm{spin}}}\Delta t^{a}+t_{i}^{s}+\left( \frac{1}{C_{i}}-\frac{1}{C_{\mathrm{ss}}} \right)\alpha\#\left( S50 \right) \end{aligned}$$

For the three wet films, which grow into the UD morphology, *t*s 01, *t*s 02 and *t*s 03 are 40 s, 44 s and 48 s, respectively, and Δ*t*a 1, Δ*t*a 2 and Δ*t*a 3 are 29 s, 17 s and 7 s. **Figure M2** shows a linear correlation between Δ*t*^a^ and *t*s 0 as described by Equation S50. From Supplementary **Figure 3a**, *t*s i is equal to *t*s ss when Δ*t*^a^ is zero. Hence, *t*s ss is equal to 50.4 s determined by the extrapolation of the *t*s 0 as shown by the intercept in **Figure M2**. Three theoretical curves in Figure 3f are obtained by combining Equation S42 with Equations S47 to S49, respectively. Figure M2 illustrates the experimentally observed trend and corresponding agreement with the model. Although only a limited number of experimental points are shown, the measured results consistently follow the predicted growth trend under different processing conditions, supporting the mechanistic validity of the model.

**Figures**

Supplementary Figure 1. Classical nucleation theory and the LaMer model. (a) Change in Gibbs free energy according to the classic nucleation theory. Δ*G*_s_ and Δ*G*_v_ are surface and volume Gibbs free energy changes, respectively. The critical radius *r*^*^ indicates the nucleation formation from a cluster. (b) Schematic illustration of the classic LaMer curve with the I, II and III stages.

In the classic nucleation theory, the change in Gibbs free energy (Δ*G*) of the system is determined by the contributions from the newly formed surface (Δ*G*_S_) and the cluster volume (Δ*G*_V_), expressed as:

$$\begin{aligned} \Delta G\left( r \right)={\Delta G}_{S}+{\Delta G}_{V}=4\pi r^{2} \gamma-\frac{4}{3}\pi r^{3}k_{b}T\frac{\ln S_{S}}{V_{m}},\#\left( S51 \right) \end{aligned}$$

where *r* is the radius of clusters, *γ* is the surface free energy per unit area of the cluster, *k*_b_ is the Boltzmann constant, *T* is the absolute temperature, *V*_m_ is the molar volume of the molecule in the cluster, and *S*_S_ is the supersaturation ratio defined as *S*_S_ = *C*/*C*_s_ where *C* is the solute concentration in the wet film. As cluster size increases, the surface area in contact with the surrounding solvent also grows, leading to an increase in Δ*G*_S_, as shown by the gray curve in Supplementary **Figure 1a**. At the same time, Δ*G*_V_ diminishes (blue curve in Supplementary **Figure 1a**) because the free energy of the clusters is lower than that of the solvent. Consequently, with increasing *r*, Δ*G* firstly rises and then drops at the critical radius *r*^*^, which is defined as the smallest cluster size for nucleation. Nucleation is initiated if ǀΔ*G*_V_ǀ > Δ*G*_S_ and the overall Δ*G* starts to decrease with increasing *r* as plotted by the red curve in Supplementary **Figure 1a**. The critical radius *r*^*^ can be obtained by setting the derivative ∂Δ*G*(*r*)/∂*r* to zero, giving:

$$\begin{aligned} r^{*}=\frac{2\gamma V_{m}}{k_{b}Tln\frac{C}{C_{s}}}\#\left( S52 \right) \end{aligned}$$

The relation between *r*^*^ and *C*, derived from Equation S52, is illustrated in **Figure 1d** (red solid curve) for the bulk case. For the interface and cusp cases, a lower *γ* leads to a smaller *r*^*^, as also shown in **Figure 1d**.

To further illustrate the nucleation and crystallization process, the LaMer plot in Supplementary **Figure 1b** describes the time evolution of nucleation and crystal growth in the solution. The LaMer curve consists of three stages: (I) initial increase of the concentration of solute at the defined time in the solution until supersaturation (*C*_ss_) is reached; (II) clusters in the wet film form initial nuclei accompanied by a concentration decrease due to solute consumption; (III) nucleation ends and the formed nuclei continue their growth leading to a decrease in concentration until the limit of solubility (*C*_s_). Notably, in our system, the initial concentration increase (Stage I) differs from the classical LaMer curve because the concentration increase in Stage I is driven by continuous solute supply in the classical LaMer model, whereas in our case, solvent evaporation causes the concentration to rise. This distinction results in a different early-stage profile while maintaining the same overall nucleation-growth sequence.

Supplementary Figure 2. Scanning electron microscopy images of (PEA)_2_SnI_4_ films with different morphologies. (a,d) large domain (LD), (b,e) unidirectional domain (UD) and (c,f) small domain (SD).

Supplementary Figure 3. Scanning electron microscopy images of (MeOPEA)_1.8_Cs_0.1_SnI_3.9_ films with different morphologies. (a,d) LD, (b,e) UD and (c,f) SD.

Supplementary Figure 4. Scanning electron microscopy images of MAPbI_3_ films with different morphologies. (a,d) LD, (b,e) UD and (c,f) SD.

Supplementary Figure 5. Scanning electron microscopy images of TIPS-pentacene films with different morphologies. (a,d) LD, (b,e) UD and (c,f) SD.

Supplementary Figure 6. Scanning electron microscopy images of NaH_2_PO_4_ films with different morphologies. (a,d) LD, (b,e) UD and (c,f) SD.

Supplementary Figure 7. Optical microscopy images of different films nucleated at different coating times, corresponding to Figure 1g. The scale bars are 400 µm for (MeOPEA)_1.8_Cs_0.1_SnI_3.9_, TIPS-pentacene and NaH_2_PO_4_, and 100 µm for MAPbI_3_.

Supplementary Figure 8. Schmatic illustration of two boundary conditions for the initiation of the UD growth (a) at the end of the processing window with maximum Δ*t*^s^ and (b) at the beginning of the processing window with maximum Δ*t*^a^.

To normalize both time intervals we define a maximum Δ*t*^s^ (Δ*t*s max) and Δ*t*^a^ (Δ*t*a max) representing two boundary conditions. In the first case depicted in Supplementary **Figure 8a** and defined by Equation S13, Δ*t*s max is obtained when *C*_0_ is equal to *C*_ss_ resulting in *t*s 0 reaching *t*s ss. The parameter Δ*t*s max expresses the longest spin-coating time interval after which the UD growth can be still initiated by induced nucleation. Hereby, Δ*t*^a^ equals to zero since *C*_ss_ is reached already during spin-coating so that the UD growth does not occur during annealing. On the other hand, Δ*t*a max is attained when *C*_0_ corresponds to *C*_i_ leading to *t*a 0 equaling *t*a i as illustrated in Supplementary **Figure 8b** and described by Equation S14. This case is related to the shortest Δ*t*^s^ after which UD growth can be initiated and requires the longest subsequent annealing time.

Supplementary Figure 9. Optical micrographs of scratched (PEA)_2_SnI_4_ films processed at different conditions (white scale bar at right bottom corresponds to 300 μm). The spin-coating time is varied from 32 s to 52 s and the concentration of precursor solution from 0.06 M to 0.12 M. The blue, red and yellow boxes correspond to morphologies LD, UD and SD, respectively.

In Supplementary Figure 9, the delayed onset of UD growth at higher precursor concentration may appear counterintuitive if only the initial concentration is considered. However, the onset of the induced nucleation is governed by the time required for the wet film to reach *C*_i_, which depends on the dynamic evolution of the concentration during solvent evaporation.

Spin-coating a solution with a lower precursor concentration typically yields a thinner wet film in which the solvent evaporation results in a larger relative reduction in volume. Consequently, the solute concentration increases more rapidly, allowing the wet film to reach *C*_i_ at an earlier time.^5^ In contrast, a solution with a higher precursor concentration forms a thicker wet film, leading to a slower relative increase in concentration and a delayed onset of the UD growth.

Supplementary Figure 10. Polarized optical micrographs of scratched TIPS-pentacene films processed at different spin-coating times and speeds at *C*^init^ of 10 mg/ml. The scale bar is 400 µm.

Supplementary Figure 11. Crystallization kinetics of (MeOPEA)_1.8_Cs_0.1_SnI_3.9_ films. (a) Optical micrographs of (MeOPEA)_1.8_Cs_0.1_SnI_3.9_ films at *C*^init^ of 0.1M processed at different spin-coating times varied from 50 s to 64 s. The scale bar is 400 µm. (b) Relation between *L*^final^ of UDs and *t*s 0. (c) Fitting curve based on the model for the relation between *L*final normal and Δ*t*s normal. Error bars represent the standard deviation of six measurements.

Supplementary Figure 12. Optical micrographs of (PEA)_2_SnI_4_ films at *C*^init^ of 0.1M deposited in a two-step spin-coating process: first 2000 rpm for 15 s and followed by 4000 rpm for different spin-coating times varied from 18 s to 28 s. The scale bar is 400 µm.

Supplementary Figure 13. Definition and experimental extraction of the characteristic lengths (a) *L*^final^ extracted from optical microscopy image and (b) *L* extracted from time-resolved movie frames during annealing.

Supplementary Figure 14. Schematic illustration of the model to describe the change of *L* during the annealing time, in contrast to Figure 2b which depicts the final length of UDs *L*^final^.

Supplementary Figure 15. Processing window for the UD growth of the (PEA)_2_SnI_4_ perovskite. The scale bar is 300 µm. (a) Movies S1 and S2 are captured when the nucleation is induced at the onset of the growth window for two films at *C*^init^ of 0.06 M and 0.12 M, respectively. (b) Movies S2 to S4 are captured when the nucleation is induced within the growth window at spin-coating time of 40 s, 44 s and 48 s, respectively.

Supplementary Figure 16. Time-dependent growth of UDs and LDs. (a) Length of the UDs of (PEA)_2_SnI_4_ as a function of annealing time during the crystallization shown in Figures 3a and 3b. (b) Area of the LDs in Figures 3d as a function of annealing time.

Supplementary Figure 17. Labeled crystallization movie frames corresponding to Figure 3a-d, with an identification of the UD, LD, and SD morphologies. Panels (a1-d1) show the UD regions highlighted by red dashed outlines. The dark areas outside the red regions correspond to LD morphologies formed through spontaneous nucleation at earlier annealing times. Panels (a2-d2) show the subsequent movie frames recorded 1 s later, where the newly emerged dense nucleation regions highlighted by yellow dashed outlines correspond to the SD morphology.

Supplementary Figure 18. Optical microscopy (left) and atomic force microscopy (right) images of (PEA)_2_SnI_4_ films with SD, LD and UD.

Supplementary Figure 19. A depiction of the experimental setup used for KPFM based on the FET setup descriped in the experimental section.

Supplementary Figure 20. Device geometry and scratch configuration for (a,d) UD, (b,e) LD and (c,f) SD (PEA)_2_SnI_4_ film morphologies. The channel is positioned adjacent to the scratch to preserve film continuity.

The channel is positioned adjacent to the scratch line to preserve film continuity while incorporating the domains into the active channel region. The scratch is located out of the channel and does not disturb the characterization of device.

Supplementary Figure 21. Photoconductivity dynamics under 3.10 eV excitations with incident pump fluence of 56 µJ cm^-2^ for (PEA)_2_SnI_4_ films with SD, LD and UD morphologies.

Supplementary Figure 22. Transient absorption spectroscopic characterization for (a) LD, (b) UD and (c) SD of PEA_2_SnI_4_ films. (d) Normalized transient absorption decay kinetics monitored at 615 nm for three films.

Supplementary Figure 23. Normalized time-resolved photoluminescence decay curves of the SD, LD, and UD films measured under identical excitation conditions.

Supplementary Figure 24. Output characteristics for FETs of (a) (PEA)_2_SnI_4_, (b) (MeOPEA)_1.8_Cs_0.1_SnI_3.9_ and (c) TIPS-pentacene based on SDs, LDs and UDs.

Supplementary Figure 25. Bias stability of FET devices based on LD, UD, and SD (PEA)_2_SnI_4_ morphologies. (a) Evolution of |*I*_ds_| under continuous bias stress at *V*_ds_ = *V*_g_ = −10 V. (b) Normalized |*I*_ds_| as a function of stress time.

The bias-stability measurements show that UD-based devices retain the highest drain current after 1000 s of continuous bias stress, followed by LD and SD devices. The current retention after 1000 s is approximately 40% for UD, 36% for LD, and 29% for SD, indicating that the UD morphology provides the highest operational stability among the three growth modes.

**Tables**

Table S1. Summary of the best-fit values of parameter pair (*R*, *S*_Si_) and coefficient of determination for different materials.

| Materials | *R* | *S*_Si_ | Coefficient of determination |
| --- | --- | --- | --- |
| (PEA)_2_SnI_4_ | 0.85 | 1.01 | 0.989 |
| TIPS-pentacene | 0.92 | 1.02 | 0.994 |
| (MeOPEA)_1.8_Cs_0.1_SnI_3.9_ | 0.81 | 1.04 | 0.995 |

Table S2. Summary of FET performance of (PEA)_2_SnI_4_ with SD, LD and UD morphologies. Error bars are obtained from ten independently fabricated devices.

| Device | *μ*_FET_  (cm^2^ V^−1^ s^−1^) | *V*_th_ (V) | *I*_on/off_ | SS (V dec^−1^) | Δ*V* (V)  \|*I*_ds_\| = 10^-6^ A |
| --- | --- | --- | --- | --- | --- |
| SD | 0.2 ± 0.06 | 10.9 ± 9.8 | (1.8 ± 0.9) ×10^4^ | 6.7 ± 2.2 | 19.5 ± 4.3 |
| LD | 0.6 ± 0.2 | 13.0 ± 5.6 | (4.0 ± 1.6) ×10^4^ | 8.1 ± 1.2 | 12.8 ± 4.9 |
| UD | 2.6 ± 0.7 | 6.1 ± 4.1 | (5.0 ± 2.8) ×10^5^ | 5.0 ± 1.4 | 10.2 ± 4.2 |

Table S3. Summary of FET performance of (MeOPEA)_1.8_Cs_0.1_SnI_3.9_ with SD, LD and UD morphologies. Error bars are obtained from ten independently fabricated devices*.*

| Device | *μ*_FET_  (cm^2^ V^−1^ s^−1^) | *V*_th_ (V) | *I*_on/off_ | SS (V dec^−1^) | Δ*V* (V)  \|*I*_ds_\| = 10^-6^ A |
| --- | --- | --- | --- | --- | --- |
| SD | 0.3 ± 0.1 | 4.5 ± 4.0 | (3.2 ± 2.9) ×10^4^ | 5.7 ± 1.0 | 15.3 ± 4.6 |
| LD | 0.8 ± 0.2 | -2.8 ± 2.2 | (9.3 ± 5.8) ×10^4^ | 5.0 ± 0.8 | 11.4 ± 2.9 |
| UD | 2.8 ± 0.2 | -2.4 ± 1.6 | (1.7 ± 0.8) ×10^5^ | 4.8 ± 0.9 | 5.8 ± 1.2 |

Table S4. Summary of FET performance of TIPS-pentacene with SD, LD and UD morphologies. Error bars are obtained from ten independently fabricated devices.

| Device | *μ*_FET_  (cm^2^ V^−1^ s^−1^) | *V*_th_ (V) | *I*_on/off_ | SS (V dec^−1^) | Δ*V* (V)  \|*I*_ds_\| = 10^-6^ A |
| --- | --- | --- | --- | --- | --- |
| SD | 0.2 ± 0.09 | -9.0 ± 8.2 | (3.5 ± 1.7) ×10^4^ | 10.4 ± 0.7 | 6.3 ± 0.8 |
| LD | 0.5 ± 0.05 | -7.5 ± 5.4 | (7.8 ± 4.3) ×10^4^ | 10.1 ± 0.5 | 7.1 ± 0.7 |
| UD | 0.7 ± 0.05 | -3.4 ± 2.7 | (1.3 ± 0.3) ×10^5^ | 9.5 ± 0.4 | 5.5 ± 0.4 |

**Supplementary references**

1.	Choi, H. H. *et al.* Critical assessment of charge mobility extraction in FETs. *Nat. Mater.* **17**, 2–7 (2017).

2.	Holzwarth, U. & Gibson, N. The Scherrer equation versus the ‘Debye-Scherrer equation’. *Nat. Nanotechnol.* **6**, 534 (2011).

3.	Richel, A., Johnson, N. P. & McComb, D. W. Observation of Bragg reflection in photonic crystals synthesized from air spheres in a titania matrix. *Appl. Phys. Lett.* **76**, 1816–1818 (2000).

4. Axt, A. *et al.* Know your full potential: Quantitative Kelvin probe force microscopy on nanoscale electrical devices. *Beilstein J. Nanotechnol.* **9**, 1809–1919 (2018).

5. Spies, M. *et al.* Solvated PbI2 Clusters Preceding the Crystallization of Lead Halide Perovskites–a UV/VIS In Situ Study. *Adv. Electron. Mater.***11**, 2500060 (2025).
