# Supplementary material for: Universal crystallization kinetics of solution-processed semiconductors
Source: Nat Commun. 2026 Oct 14;17:10785. doi: 10.1038/s41467-026-78354-7 (PMC13669315; doi:10.1038/s41467-026-78354-7)
Supplement: Supplementary file 3 [file 41467_2026_78354_MOESM2_ESM.docx]

Description of Additional Supplementary Files

**Supplementary Movie 1:**

Crystallization of a (PEA)2SnI4 wet film with an initial concentration of 0.06 M, scratched after a spin-coating time of 34 s.

**Supplementary Movie 2:**

Crystallization of a (PEA)2SnI4 wet film with an initial concentration of 0.12 M, scratched after a spin-coating time of 40 s.

**Supplementary Movie 3:**

Crystallization of a (PEA)2SnI4 wet film with an initial concentration of 0.12 M scratched after a spin-coating time of 44 s.

**Supplementary Movie 4:**

Crystallization of a (PEA)2SnI4 wet film with an initial concentration of 0.12 M, scratched after a spin-coating time of 48 s.
